# Projections of human kinship for all countries

**DOI:** 10.1073/pnas.2315722120

**Published:** 2023-12-19

**Authors:** Diego Alburez-Gutierrez, Iván Williams, Hal Caswell

**Affiliations:** ^a^Kinship Inequalities Research Group, Max Planck Institute for Demographic Research, Rostock 18057, Germany; ^b^Faculty of Economics, Actuarial Department, University of Buenos Aires, Buenos Aires C1120AAQ, Argentina; ^c^Theoretical and Computational Ecology Department, Institute for Biodiversity and Ecosystem Dynamics, University of Amsterdam, Amsterdam 1090 GE, The Netherlands

**Keywords:** kinship, projections, population aging, social support, mathematical models

## Abstract

Demographers have long attempted to project future changes in the size and composition of populations, but have ignored what these processes will mean for the size, composition, and age distribution of family networks. Kinship structures matter because family solidarity—a crucial source of informal care for millions of people around the world—is conditional on kin being alive. Here, we present innovative projections of biological kin for the 1950 to 2100 period and discuss what they imply for the availability of informal care. Overall, we project that the number of living kin for individuals will decline dramatically worldwide. While a 65-yo woman in 1950 could expect to have 41 living kin, a 65-yo woman in 2095 is projected to have just 25 [18.8 to 34.7] relatives (lower and upper 80% projection intervals). This represents a 38% [15 to 54] global decline. The composition of family networks is also expected to change, with the numbers of living grandparents and great-grandparents markedly increasing, and the numbers of cousins, nieces and nephews, and grandchildren declining. Family networks will age considerably, as we project a widening age gap between individuals and their kin due to lower and later fertility and longer lifespans. In Italy, for example, the average age of a grandmother of a 35-yo woman is expected to increase from 77.9 y in 1950 to 87.7 y [87.1 to 88.5] in 2095. The projected changes in kin supply will put pressure on the already stretched institutional systems of social support, as more individuals age with smaller and older family networks.

## Significance

Rapid demographic change is expected to transform the supply of kin worldwide. Changes in the size and composition of kinship networks matter because relatives provide important informal support by exchanging resources and time, even in settings with advanced welfare systems. But kin supply does not equal kin availability. For example, we project that great-grandparents will be more common in the future, but they may be too old and frail to provide support. Individuals in the future will face increasing demands for informal care from kin worldwide, albeit with significant regional variation. Our findings support the calls for more investment in childcare and old-age care to alleviate the burden of individuals aging with fewer kinship resources to rely on.

Demographic rates cast long shadows over populations and family structures. Consider the case of Focal, a girl born today. The likelihood that she has one or more living grandparents is affected by the ages at which her grandmother and her mother gave birth and by the survival rates experienced by her grandparents. The number of cousins Focal has depends on the fertility and survival of her aunts and uncles, who are the parents of those cousins. These aunts and uncles are, in turn, the offspring of the grandparental generation. Future demographic behavior also plays a role. The number of grandchildren who may attend Focal’s funeral, assuming she dies in old age, is conditioned by future rates of fertility (affecting the reproduction of Focal and of her children) and survival (affecting the survival of Focal’s children and grandchildren).

Family networks are expected to play an essential role in the context of global population aging, which will bring about higher dependency ratios and increased pressure on social security systems (1). This is because family solidarity—i.e., the exchange of time, emotional, and financial resources within and across generations—is conditioned by kin availability. Put simply, relatives must (at the very least) be alive in order to provide support. Despite great societal interest in the topic, e.g., ref. 2, we know surprisingly little about contemporary kinship structures around the world, and we know even less about how they may develop in the years to come.

The case of grandparents is a good example (3). When grandparents are involved with their grandchildren, they facilitate parental engagement with the labor market (2, 4). This grandparental support may be beneficial for parents (5), for grandchildren (6) and for grandparents themselves (3), even though caring for others can also take a toll on the well-being of grandparents (7). A large number of studies have studied the (mainly positive) effects of providing and receiving informal support for other types of kin as well (7–9).

Four main demographic trends are expected to shape future kinship structures: declines in mortality at very young and at old ages and lower and delayed fertility. Reductions in infant and child mortality lead to higher offspring availability, and, over time, to larger cohorts of siblings and cousins. Longer lives increase the prevalence of multi-generational families and the length of the periods during which the lives of grandparents and grandchildren overlap (10). However, later and lower fertility may decrease the likelihood of individuals becoming grandparents in the first place. This could result in more durable family bonds as the lives of relatives overlap for longer periods (7). Alternatively, it could mean that grandparents are too old and frail to provide support and instead become net consumers of informal care (9).

The existing historical (11, 12) and conceptual (8, 13) analyses of kinship are limited because they mostly pertain to low-fertility contexts in Europe and North America. This restricts their applicability to regions of the Global South. For example, it is unclear whether lower mortality and fertility will produce “beanpole” kinship networks dominated by vertical kin (14), in sub-Saharan Africa, where future population growth will be concentrated (15). Similarly, previous studies on the populations of the Global North often focused less on informal care based on the assumption that some form of institutional support (i.e., state-sponsored childcare, pensions, and old-age care; pensions or market-based solutions such as paid services) is available to individuals. Indeed, family solidarity may matter less in contexts where strong institutions provide individuals with reliable access to healthcare, childcare, and old-age support (16). However, most of the world population is heavily reliant on informal care from relatives.

The missing ingredient in these studies, required to resolve these uncertainties, is projections of kinship networks that are linked to, and comparable in detail to, the projections of population size and structure produced by statistical offices and international organizations. In this paper, we present such projections and use them to ask how demographic change will shape the supply of kin for future generations. We compute kinship structures for all the countries in the world, connecting historical dynamics from 1950 to the present, and projections of the future to the year 2100. We produce kinship projections by year, age of a female focal individual, and age and sex of kin for a range of biological relatives.

The formal demography of kinship began with the work of (17), who modeled a kinship network as a property of a focal individual. That model, limited in its flexibility, has been replaced by a matrix-based theory that incorporates age-classified, multi-state, two-sex, and, most importantly for our study, time-varying demographic rates (18–21). This framework treats kinship dynamics by projecting kin populations. We combine formal kinship models with the output of probabilistic demographic projections prepared by the U.N. (1, 22, 23). This creates a projection that includes both nuclear and extended kin and allows us to discuss changes in the availability of informal support in light of the projected global demographic changes.

## Results

We use two-sex matrix kinship models (20) to project each kin type, linked to its ancestors and descendants, in a time-variant framework (21) that is closed to migration. The model produces expected values, for a female member of the population of a specified age, of the age-sex distribution of each type of kin for each calendar year. Our mathematical implementation is more efficient than simulation-based kinship methods which could be used to produce equivalent results, e.g., ref. 13. For the 1950 to 2021 period, we obtained period female fertility rates and male and female survival probabilities from the 2022 Revision of the World Population Prospects (UNWPP) (1). The projections of future population produced by the U.N. are stochastic. We computed 1,000 kinship trajectories for each country by sampling from the posterior distribution of the Bayesian projections of fertility (22) and mortality (23) produced for the UNWPP.

We report country-level kinship structures for a randomly selected woman in the population, whom we call Focal. We focus on a female focal person because women provide the lion’s share of informal care in most countries and are more likely than men to experience kinlessness later in life (24). We conducted the analysis in 5-y intervals for computational efficiency (*SI Appendix*). Our estimates refer to the middle value of the interval; however, for convenience, in the text, we refer to single years rather than year intervals (e.g., “2095” refers to the 2095 to 2100 period and “65 yo” refers to a person aged 65 to 69). We provide full results by type of kin, country, year, and age of Focal in *SI Appendix*.

### Kinship Networks Are Shrinking around the Globe.

**Fig. 1.**
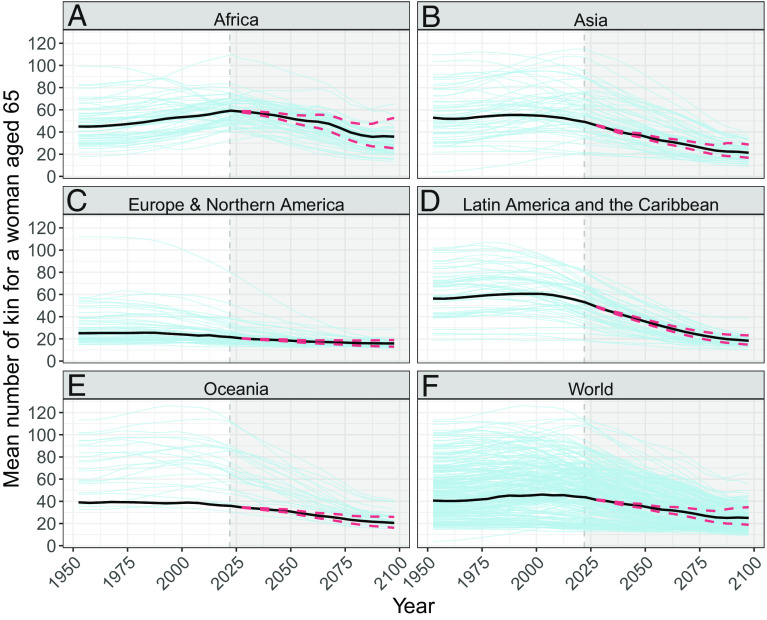
Total family size the sum of all living great-grandparents, grandparents, parents, children, grandchildren, great-grandchildren, aunts/uncles, nieces/nephews (i.e., niblings), siblings, and cousins for a 65-yo female Focal in different world regions (*PanelsA*–*E*) and globally (*PanelF*). The light-blue lines in the background show country-level values (median projection trajectory after 2021). The thick black lines indicate the regional values; after 2021, the black lines represent the projection median and the red lines represent the 80% projection intervals for each region (averages weighted by country-level population size at Focal’s age 65).

We start by presenting regional trends in total family size, defined as the totality of Focal’s living great-grandparents, grandparents, parents, children, grandchildren, great-grandchildren, aunts/uncles, nieces/nephews (i.e., niblings), siblings, and cousins. Fig. 1 shows the total family size for an individual aged 65, an age chosen as a proxy for retirement from formal employment, by world region (panels *A*–*E*) and for the entire world (panel *F*). Overall, we find large regional differences in total family size in the present, and we project that a sustained decline in family size will occur in the future, both globally and for each of the regions we consider (regional values come from country-level estimates weighted by population size at each age of Focal).

We expect the largest declines in family size to occur in Latin America and the Caribbean. In this region, a 65-yo Focal in 1950 could expect to have about 56 living relatives, whereas a 65 yo in 2095 is projected to have around 18.3 [14.7 to 23.1] (lower and upper 80% projection intervals) kin. This represents a 67% [59 to 74] decline in total family size.

We expect little change in family size to occur in regions where family structures are already relatively small. In Europe and North America, the family size for a 65 yo is projected to decline from 25 in 1950 to 15.9 [12.9 to 19] in 2095, which is a 37% [24 to 48] reduction. Fig. 1*F* shows the historical and projected trends for the entire world: Globally, total family size for a 65 yo will decline from 41 in 1950 to 25 [18.8 to 34.7] in 2095. We project a shrinking gap between the region with the smallest total family size and the region with the largest total family size in the period considered. The gap in 1950 is 31 relatives: In that year, an average 65-yo Focal in Latin America and the Caribbean could expect to have 56 living kin, whereas a woman of the same age in Europe and North America could expect to have just 25 relatives. By 2095, we project that the largest gap in family size between any two regions will shrink to 20 [12.4 to 33.75] relatives; i.e., between Africa with 35.9 [25.3 to 52.7] living kin in Africa and Europe and North America with 15.9 [12.9 to 19] living relatives.

**Fig. 2.**
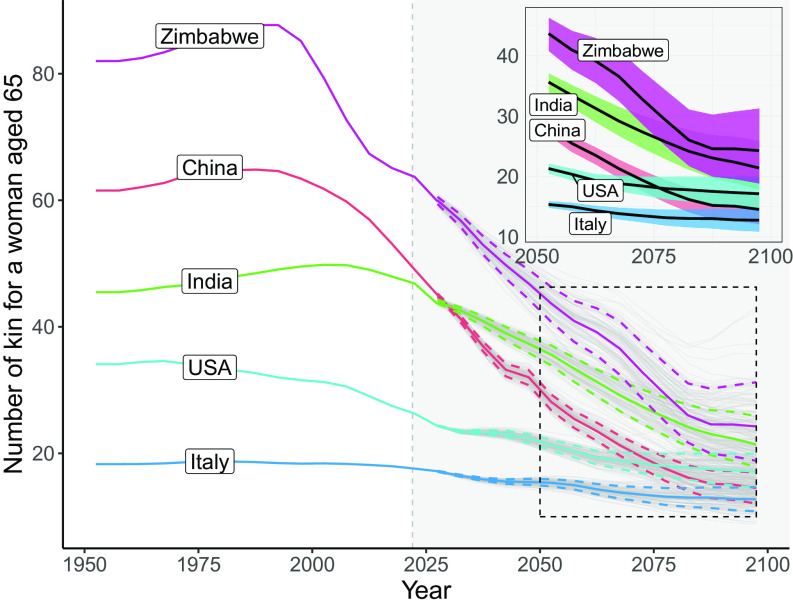
Average number of living kin for a Focal woman aged 65 in selected countries. The gray lines in the background after 2021 show 100 trajectories from the 2022 Revision of the United Nations World Population Prospects. The continuous lines show the median of these projections, and the dashed lines indicate the 80% projection intervals.

We now explore some of the within-region heterogeneity by focusing on the total family size of women aged 65 in five countries selected to represent a wide range of historical and projected demographic trajectories. Fig. 2 shows that while an average Zimbabwean woman approaching age 65 in 1950 could expect to have 82 living relatives, her counterpart in 2095 is projected to have just 24.1 [18.3 to 32] relatives, which represents a 71% [61 to 78] decline. In Italy, the country with the smallest family size in Fig. 2, an average 65-yo woman could expect to have 18 relatives in 1950 and is projected to have 12.7 [10.3 to 15.3] relatives in 2095, which is a decline of just 30% [16 to 44].

The projections of kin show a general convergence of total family size in the five selected countries. This is likely driven by the convergence of demographic components built into the UNWPP projections, even after accounting for the uncertainty derived from the probabilistic projections. For 1950, the estimated difference in the average number of living kin between the country with the largest family size and the country with the smallest family size is 63 relatives (Zimbabwe = 82; Italy = 18). As shown in Fig. 2, by 2095 this gap is projected to decline to just 11 relatives (Zimbabwe = 24.1 [18.3 to 32]; Italy = 12.7 [10.3 to 15.3]). This narrowing of differences clearly indicates that individuals living in different regions of the world can expect to have increasingly similar family sizes. However, as we will see next, the composition of kinship networks in terms of the types of relatives and the ages of kin will continue to vary significantly.

### The Composition of Kinship Networks Is Changing.

Kinship networks are expected to change not just in terms of their overall size, but also in terms of their kin configuration. For example, more “horizontal” families are dominated by lateral kin like siblings and cousins, whereas more “vertical” families have a higher proportion of ascendants and descendants like children and grandparents (14).

The impact of the “one-child policy” (1979 to 2015) and related restrictions on reproduction in China offers an example of how the composition of kinship networks can vary over time as a result of rapid demographic change. The current Chinese population is already experiencing an acute marriage squeeze, and it is expected that the thinning-out of kinship networks will put unprecedented pressure on informal and formal care providers in the near future. Below, we show the estimated number of kin for an average Chinese woman over time at two key stages of Focal’s life: at birth and at age 65.

**Fig. 3.**
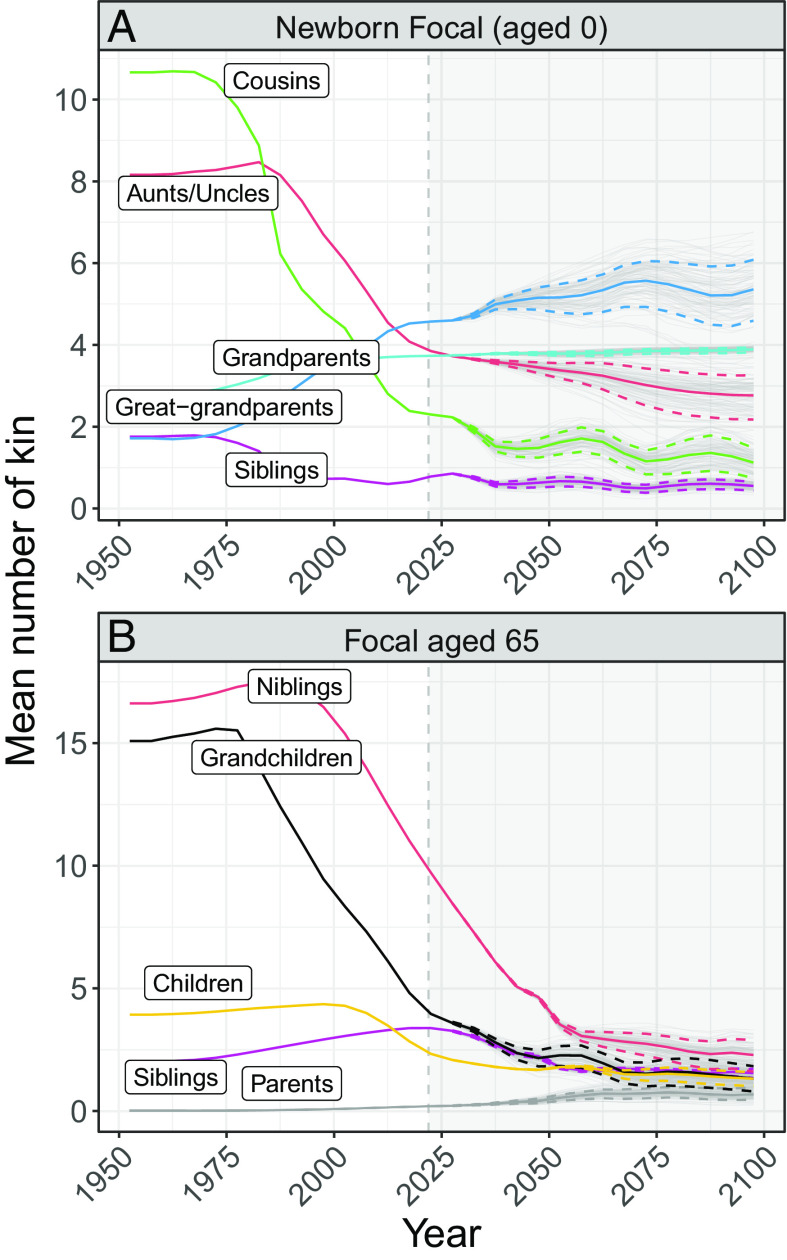
Expected number of selected kin for a new-born woman (*Panel A*) and for a Focal aged 65 (*Panel B*) in China. The gray lines in the background after 2021 show 100 trajectories estimated using probabilistic trajectories from the 2022 Revision of the United Nations World Population Prospects. The thick lines indicate the median estimates and the dashed lines show the 80% projection intervals.

Fig. 3*A* shows a radical reversal in the most predominant type of kin at the start of Focal’s life. The family network of a Chinese newborn in 1950 is largely dominated by cousins (about 11 cousins, representing 39% of her total family network). By contrast, in the year 2095, a Chinese newborn will have, on average, just 1.1 [0.7 to 1.5] cousins (7% [5.4 to 8.5] of her total family network). At the same time, however, this newborn’s number of living ancestors is projected to increase, as the probability that all four of her grandparents will be alive at her birth is expected to approach 100% in the near future. An even more remarkable finding is the projected growth in the number of great-grandparents. A newborn in 1950 could expect to have more living grandparents (2.8) than great-grandparents (1.7). A woman born in 2095, by contrast, can expect to have 5.3 [4.6 to 6.1] living great-grandparents, or 3.1 [2.7 to 3.6] times more than her counterpart born in 1950.

Fig. 3*B* shows a remarkable convergence over time in the numbers of different types of relatives older individuals are projected to have. In 1950, an average 65-yo Chinese woman could expect to have around 15 grandchildren, but was unlikely to have a living parent. In 2095, a 65-yo Focal is projected to have 0.7 [0.5 to 1] living parents, 30 [21 to 42] times more than in 1950, and just 1.3 [0.8 to 1.8] grandchildren, 11 [8 to 19] times less than in 1950. We observe a similar pattern for the other kin types depicted in the figure, with the number of kin of each type expected to hover between 0.7 [0.5 to 1] (parents) and 2.2 [1.6 to 2.9] (nieces/nephews, or niblings) in 2095. This is in stark contrast to the number of relatives of each type in 1950, which ranges from 0.02 (parents) to 16.6 (niblings).

### A Growing Age Gap between Individuals and Their Kin.

Kinship networks vary in terms of how old their members are. In demography, older populations have high ratios of older to younger individuals. We can think of kinship structures in a similar way, with an “older family network” being one with a higher ratio of older to younger kin (seen from the perspective of Focal). In this sense, the age composition of Focal’s kinship network changes over her life, and varies depending on the type of relative considered. For example, when Focal is born, her parents will on average be somewhere between 20 and 30 yo, and her siblings will be, by definition, older than Focal (since Focal cannot have younger siblings at the time of her birth).

**Fig. 4.**
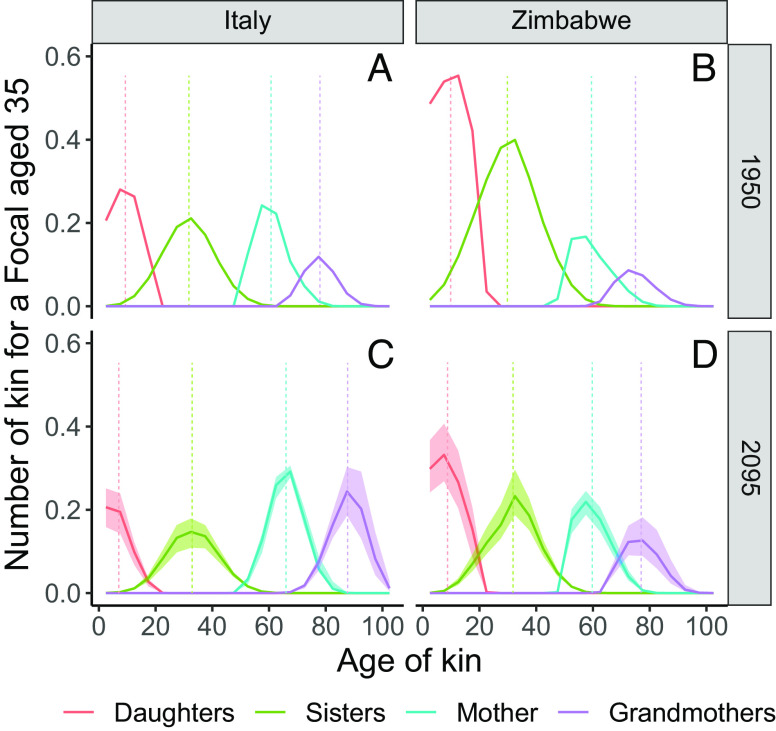
Age composition of selected female relatives for a Focal woman aged 35 in Zimbabwe and Italy (1950 and 2095) (*A*–*D*). The shaded areas for 2095 show the 80% projection intervals of 1,000 individual probabilistic trajectories. The vertical lines indicate the mean age for each relative type.

In this section, we consider changes in the age distribution of kin for a Focal aged 35. We have chosen this age because it represents a point in the life course in which Focal is likely to be “sandwiched” between generations, having both young children who require care and relatively old parents who may be sources of support or recipients of care (25). The plots in Fig. 4 should be interpreted as population age distributions, with each “population” being comprised exclusively of Focal’s relatives (e.g., the population of grandmothers, of cousins, etc.). The age distribution of each kin type integrates into the expected number of kin. The vertical lines show the average age of each kin type for our 35-yo Focal. In the projections (Fig. 4 *C* and *D*), the shaded areas indicate the lower and the upper 80% projection intervals around the median of the 1,000 country-level probabilistic trajectories. Note that these age distributions refer to female kin only.

Fig. 4 shows that for a Focal aged 35 in 1950 in Italy, her living grandmothers (*A*) are 77.9 yo, her mother is 60.8 yo, her sisters are 31.7 yo, and her children are 9.3 yo. Note that here, the age difference between the mothers and the grandmothers is just 17 y because we are only considering those grandmothers who survived to Focal’s age 35. For a Focal aged 35 in Zimbabwe in 1950 (*B*), the corresponding values are 74.9 yo for her grandmothers, 59.5 yo for her mother, 30 yo for her sisters, and 10 yo for her daughters. Fig. 4 *C* and *D* reveal a marked aging of kin over time: All kin types (except for daughters) are older in 2095 than they are in 1950 for a 35-yo Focal. In Italy, Focal’s grandmothers will have aged 9.8 y [9.2 to 10.7] between 1950 and 2095, her mother will have aged 5.2 y [5.5 to 5.7], and her sisters will have aged 1.3 y [1.2 to 1.7]. Focal’s daughters, by contrast, will be 2.3 y [2.2 to 2.6] younger (given delayed fertility). The equivalent values for Zimbabwe are 2 y [1.5 to 3] for Focal’s grandmothers, 0.5 y [0.4 to 0.8] for her mother, 1.9y [1.5 to 2.1] for her sisters, and 1.2 y [1 to 1.7] (younger) for her daughters.

## Discussion

This article examined the implications of global demographic change for the supply of kin over the lifecourse of individuals. Our analyses used schedules of birth and death to infer kinship structures following the mathematical principles outlined by Goodman et al. 17 and Caswell 20.

We projected three main trends in the global supply of biological kin. First, lower and later fertility will reduce the number of living kin considerably over the lifecourse of individuals. Globally, total family size will decline from 41 relatives in 1950 to 25 [18.8 to 34.7] kin in 2095 for women aged 65. In countries like Zimbabwe, the decline in total family size will be dramatic, decreasing 71% [61 to 78] decline in total family size during the observation period from 82 kin in 1950 to 24 [18 to 32] kin in 2095. Second, family networks will become more “vertical” over time. Kinship structures worldwide will be increasingly composed of (great)grandparents, parents, children, and (great)grandchildren. Horizontal kin will be in relatively low supply. For example, in China, cousins comprise 39% of the family network of a newborn in 1950, but they will make up just 7% [5.4 to 8.5] of a newborn’s family network in 2095. Third, kinship networks will age considerably, as evidenced by a growing age gap between individuals and their relatives. The aging of relatives will be more pronounced in countries that currently have relatively young age structures; although even in countries like Italy, which has one of the world’s oldest populations, grandparental age is projected to increase from 78y in 2020 to 87.7 [87 to 88.5] in 2095.

This study highlights the need to invest in robust systems of social support to ensure the well-being of individuals at all life stages. Projections of kin are crucial in the context of rapid population aging, as increasingly small birth cohorts are expected to look after older adults who have fewer siblings and cousins (26) or no kin at all (24, 27). Our results provide further evidence that the supply of kinship resources is dwindling worldwide. Thus, individuals in the future can expect to have smaller family networks from which to draw support at key stages of the lifecourse. It is tempting to think that the growing number of grandparents and great-grandparents may relieve parents of some childcare responsibilities. However, these (great)grandparents may be old and frail and in need of care themselves. As a result, the growing periods of generational overlap are likely to increase the burden of care for middle-aged individuals who must look after their own parents and grandparents in failing health.

Finally, the degree to which kin supply will translate into informal care or an increased demand for it will be influenced by the changing age distribution of morbidity, increasing union instability, growing geographic distance between kin, the rising retirement age, and changes in the cultural expectations around the provision of care (28). The relevance of kin availability for individuals also depends on institutional contexts. Informal care may be less relevant in settings with advanced welfare systems (16), and kin functions may weaken in some emerging economies as they develop institutional systems of care. Nevertheless, large swaths of the world’s population do not, at present, have access to sophisticated systems of social support, healthcare, and childcare at present. For them, family ties remain a crucial source of support and informal care (29), and this is likely to continue to be the case in the future.

It is worth noting that our results pertain to hypothetical populations subject to four main simplifying assumptions. First, male fertility schedules are not available for all the countries, over all the time periods, that we study here (30). Therefore, we approximated male fertility by setting it equal to female fertility. (20) showed that this “androgynous approximation” produces excellent results for total numbers of kin, even when the differences between male and female fertility schedules are large. In *SI Appendix*, we explore the approximation for the case of Sweden and show that the androgynous approximation only slightly overestimates the number of male ancestors (grandparents and great-grandparents). We decided that this was an acceptable trade-off for extending the geographic coverage of the study.

Second, the kinship projection, like any population projection, requires an initial condition as a starting point. As an initial (1950) condition, we used the kinship network that would have been produced by time-invariant rates operating before 1950. This may have affected our projections of kinship structures in populations that experienced period effects such as a post-WWII “baby boom.” Two recent multi-country simulation studies (25, 31) found that the resulting bias of this assumption is negligible and empirically undetectable after 2015, which implies that our projections of future kin should be unaffected by it. This is because the effects of our time boundary condition, the assumption that the 1950 rates have been operating for a long time (21), dissipate over time.

Third, our country-level study is based on age- and sex-specific demographic rates and thus has nothing to say about within-country heterogeneity due to other characteristics (see ref. 19 for an approach to including multistate rates). The only empirical study to have studied this topic found relatively low levels of socio-economic heterogeneity in kinship structures in Sweden (32). However, kinship heterogeneity may be higher in countries with higher levels of socio-economic inequality. In *SI Appendix*, we show that our model estimates are consistent with empirical kinship data.

Fourth, the matrix kinship model projects the kinship network as a population, all of whose members are subject to the same mortality and fertility schedules (age- and sex-specific in our case). This implies that the demographic rates are independent within and across generations. It is worth keeping in mind that these results come from demographic projections which, as noted by ref. 33, are “the numerical consequences of the assumptions chosen.”

There is considerable potential for follow-up studies on kin availability. Our focus on biological kin ignored socially fashioned kin ties, which are common around the world (34). Future work can extend our work to include spouses, in-laws, and other potential kin ties as well as kinship projections by socio-economic status or levels of educational attainment using multistate kinship models (19). Similarly, we did not consider factors such as the strength or cultural significance of kin ties, the inter-generational transmission of resources, propinquity, or the many other ways in which family relations may be enacted. Our analysis focused on a focal individual, but future population-level analyses should take common ancestry into account: The fact that, especially in places with high fertility, the same ancestors will be shared by many individuals. Future work can extend our models to incorporate these considerations into projections of informal care availability.

## Materials and Methods

Our estimates provide the kinship network of a randomly selected woman, alive at a specified age. We call this individual Focal. Here, we exemplify our modeling approach by showing how to compute the age distribution of Focal’s granddaughters. For the sake of simplicity, we consider a female matrilineal population (21). The full model specifications used in this paper are included in *SI Appendix*, section 1.

Let a(x,t) be a vector representing the population of Focal’s daughters at age x of Focal at time t, and let b(x,t) be the same, but for the granddaughters of Focal. Let us assume that we know a(x,t) and b(x,t) and want to calculate b(x+1,t+1). Below, $U_{t}$ is a transition matrix with age-specific survival probabilities on the subdiagonal and $F_{t}$ is a matrix with age-specific fertility rates in the first row. We project the kinship dynamics of Focal’s granddaughters when Focal is aged x+1 years at time t+1 as:

[1] $$\begin{array}{c} \underset{\begin{array}{c} \;\text{distribution of}\\ \;\text{granddaughters} \end{array}}{\underbrace{b(x+1,t+1)}}=\underset{\begin{array}{c} \;\text{surviving}\\ \;\text{granddaughters} \end{array}}{\underbrace{U_{t}\;b\left(x,t\right)}}+\underset{\begin{array}{c} \;\text{new}\\ \;\text{granddaughters} \end{array}}{\underbrace{F_{t}\;a\left(x,t\right)}}. \end{array}$$

Eq. **1** states that the expected number of granddaughters of Focal at age x+1 in time t+1 is the result of the survival of the existing granddaughters from time t and any additional granddaughters of Focal borne in the time interval t to t+1 by the daughters at time t and age x of Focal. Calculations for other relative types follow a similar logic, with adjustments to account for the different ways in which Focal acquires and loses kin (e.g., cousins are the result of the reproduction of living aunts and uncles). Specifications for all kin types are given in ref. 21.

Our main analysis uses a two-sex time-variant kinship model (20), meaning that rates vary at regular intervals and male and female kin can reproduce along both matrilineal and patrilineal lines. The model takes as input period male and female survival probabilities and female age-specific fertility rates, both of which use 5-y age intervals and vary on 5-y time intervals. Thus, our time unit is 5 y, so, e.g., the interval from t to t+1 is five calendar years. Our projections of kinship rely exclusively on survival probabilities and fertility rates and are independent of union formation dynamics (see *SI Appendix* for more details). All analyses were conducted in R using the DemoKin package (35).

Historical demographic rates for the analyses (for the 1950 to 2021 period) come from the 2022 Revision of the World Population Prospects (UNWPP).^*^ For rate projections (in the 2022 to 2100 period), we use 1,000 individual trajectories of mortality, fertility, and population size by age and sex projected for each country by the UNWPP using Bayesian methods (22, 23). In *SI Appendix*, we present the median of the 1,000 kinship models for all countries.

## Supplementary Material

Appendix 01 (PDF)

## Data Availability

The code to reproduce the results is available at (36). Country-level estimates of kin availability by type of kin, country, year, and age of Focal are available in *SI Appendix*. Full results, by type of kin, country, year, age of Focal, and age of kin, including median and lower and upper 80% projection intervals, are available at the Harvard Dataverse (37).

## Footnotes

^*^https://population.un.org/wpp/. Accessed 12 January 2023.

## References

[1] UN DESA, “World Population Prospects 2022: Summary of Results” (United Nations Department of Economic and Social Affairs, Population Division, Tech. Rep. UN DESA/POP/2022/TR/NO. 3, 2022).

[2] The Economist, The age of the grandparent has arrived. *Econ.*, 12 January 2023, pp. 53–55.

[3] M. Danielsbacka, L. Křenková, A. O. Tanskanen, Grandparenting, health, and well-being: A systematic literature review. Eur. J. Ageing **19**, 341–368 (2022). 36052183 10.1007/s10433-021-00674-y PMC9424377

[4] R. Margolis, A. M. Verdery, A cohort perspective on the demography of grandparenthood: Past, present, and future changes in race and sex disparities in the United States. Demography **56**, 1495–1518 (2019). 31270779 10.1007/s13524-019-00795-1 PMC6667684

[5] A. Backhaus, M. Barslund, The effect of grandchildren on grandparental labor supply: Evidence from Europe. Euro. Econ. Rev. **137**, 103817 (2021).

[6] R. Sear, D. Coall, How much does family matter? Cooperative breeding and the demographic transition Popul. Dev. Rev. **37**, 81–112 (2011). 21280366 10.1111/j.1728-4457.2011.00379.x

[7] G. O. Hagestad, “The aging society as a context for family life in aging and ethics” in *Contemporary Issues in Biomedicine, Ethics, and Society*, N. Jecker, Ed. (Humana Press, Totowa, NJ, 1992).

[8] K. W. Wachter, Kinship resources for the elderly. Philos. Trans. R. Soc. London. Ser. B: Biol. Sci. **352**, 1811–1817 (1997). 9460065 10.1098/rstb.1997.0166 PMC1692136

[9] D. Dukhovnov, J. M. Ryan, E. Zagheni, The impact of demographic change on transfers of care and associated well-being. Popul. Res. Policy Rev. **41**, 2419–2446 (2022). 10.1007/s11113-022-09736-0 PMC11981011 40206151

[10] X. Song, R. D. Mare, Shared lifetimes, multigenerational exposure, and educational mobility. Demography **56**, 891–916 (2019). 31098951 10.1007/s13524-019-00772-8 PMC6823084

[11] M. Murphy, Long-term effects of the demographic transition on family and Kinship networks in Britain. Popul. Dev. Rev. **37**, 55–80 (2011). 21280365 10.1111/j.1728-4457.2011.00378.x

[12] A. M. Verdery, Links between demographic and Kinship transitions. Popul. Dev. Rev. **41**, 465–484 (2015).

[13] E. A. Hammel, Demographic dynamics and Kinship in anthropological populations. Proc. Natl. Acad. Sci. U.S.A. **102**, 2248–2253 (2005). 15677714 10.1073/pnas.0409762102 PMC548587

[14] G. O. Hagestad, Demographic change and the life course: Some emerging trends in the family realm. Famil. Relat. **37**, 405 (1988).

[15] J. Bongaarts, Africa’s unique fertility transition: Africa’s unique fertility transition. Popul. Dev. Rev. **43**, 39–58 (2017).

[16] V. Bordone, B. Arpino, A. Aassve, Patterns of grandparental child care across Europe: The role of the policy context and working mothers’ need. Ageing Soc. **37**, 845–873 (2017).

[17] L. A. Goodman, N. Keyfitz, T. W. Pullum, Family formation and the frequency of various kinship relationships. Theor. Popul. Biol. **5**, 1–27 (1974). 4818405 10.1016/0040-5809(74)90049-5

[18] H. Caswell, The formal demography of Kinship: A matrix formulation. Demogr. Res. **41**, 679–712 (2019).

[19] H. Caswell, The formal demography of Kinship II: Multistate models, parity, and sibship. Demogr. Res. **42**, 1097–1146 (2020).

[20] H. Caswell, The formal demography of Kinship IV: Two-sex models and their approximations. Demogr. Res. **47**, 359–396 (2022).

[21] H. Caswell, X. Song, The formal demography of Kinship III: Kinship dynamics with time-varying demographic rates. Demogr. Res. **45**, 517–546 (2021).

[22] A. E. Raftery, J. L. Chunn, P. Gerland, H. Ševčíková, Bayesian probabilistic projections of life expectancy for all countries. Demography **50**, 777–801 (2013). 23494599 10.1007/s13524-012-0193-x PMC3904289

[23] L. Alkema , Probabilistic projections of the total fertility rate for all countries. Demography **48**, 815–839 (2011). 21748544 10.1007/s13524-011-0040-5 PMC3367999

[24] R. Margolis, A. M. Verdery, Older adults without close kin in the United States. J. Gerontol.: Ser. B **72**, 688–693 (2017). 10.1093/geronb/gbx068 PMC5927096 28575387

[25] D. Alburez-Gutierrez, C. Mason, E. Zagheni, The “sandwich generation’’ revisited: Global demographic drivers of care time demands. Popul. Dev. Rev. **47**, 997–1023 (2021).

[26] A. Verdery, C. Campbell, Social support in America: Stratification and trends in access over two decades. Soc. Forces **98**, 725–752 (2019). PMC12178593 40538460

[27] A. M. Verdery, R. Margolis, Projections of white and black older adults without living kin in the United States, 2015 to 2060. Proc. Natl. Acad. Sci. U.S.A. **114**, 11109–11114 (2017). 28973934 10.1073/pnas.1710341114 PMC5651770

[28] E. Agree, K. Glaser, “Demography of informal caregiving” in *International Handbook of Population Aging, International Handbooks of Population*, P. Uhlenberg, Ed. (Springer, Dordrecht, 2009), vol. 1.

[29] B. P. Urdinola, J. A. Tovar, Eds., Time Use and Transfers in the Americas: Producing, Consuming, and Sharing Time Across Generations and Genders (Springer International Publishing, Cham, 2019).

[30] B. Schoumaker, Male fertility around the world and over time: How different is it from female fertility? Popul. Dev. Rev. **45**, 459–487 (2019).

[31] M. Snyder, D. Alburez-Gutierrez, I. Williams, E. Zagheni, Estimates from 31 countries show the significant impact of COVID-19 excess mortality on the incidence of family bereavement. Proc. Natl. Acad. Sci. U.S.A. **119**, e2202686119 (2022). 35737829 10.1073/pnas.2202686119 PMC9245632

[32] L. Andersson, M. Kolk, Kinship and socioeconomic status: Social gradients in frequencies of kin across the life course in Sweden. *Pop Studies* (2023). 10.1080/00324728.2023.2266403 38018858

[33] N. Keyfitz, On future population. J. Am. Stat. Assoc. **67**, 347–363 (1972). 12309295 10.1080/01621459.1972.10482386

[34] F. F. Furstenberg, L. E. Harris, L. M. Pesando, M. N. Reed, Kinship practices among alternative family forms in western industrialized societies. J. Marria. Family **82**, 1403–1430 (2020). 10.1111/jomf.12712 PMC8294648 34305172

[35] I. Williams, D. Alburez-Gutierrez, H. Caswell, X. Song, DemoKin: 1.0.3 (2023). https://CRAN.R-project.org/package=DemoKin. 10.1073/pnas.2315722120 PMC10756196 38113253

[36] D. Alburez-Gutierrez, I. Williams, H. Caswell, Replication code for “projections of human kinship for all countries" (2023). GitHub: https://github.com/IvanWilli/kin_world_proj. 10.1073/pnas.2315722120 PMC10756196 38113253

[37] D. Alburez-Gutierrez, I. Williams, H. Caswell, Replication data for “projections of human kinship for all countries” (2023). Harvard Dataverse v.1. 10.7910/DVN/FKCRHW. PMC10756196 38113253

