# Supplementary material for: Projections of human kinship for all countries
Source: Proc Natl Acad Sci U S A. 2023 Dec 19;120(52):e2315722120. doi: 10.1073/pnas.2315722120 (PMC10756196; doi:10.1073/pnas.2315722120)
Supplement: Supplementary file 1 — Appendix 01 (PDF) [file pnas.2315722120.sapp.pdf]

Supporting Information for

Projections of human kinship for all countries

Diego Alburez-Gutierrez, Iván Williams, and Hal Caswell

Diego Alburez-Gutierrez
E-mail: alburezgutierrez@demogr.mpg.de

This PDF file includes:

Supporting text
Figs. S1 to S20
Tables S1 to S4
SI References

Supporting Information Text

The matrix kinship model

Our analysis is based on a kinship model that is described in great detail in a series of published papers. Each of these publications adds additional demographic processes. (1) presents the basic model: single-sex, age-structured, and time-invariant demographic rates. (2) incorporates multistate demography, with an application to age-parity classification. (3) incorporates time-varying demographic rates. (4) extends the model to include both sexes, with the ability to include male and female fertility and mortality schedules when available. (5) expands the analysis of the death of kin to include competing causes of death. All these papers are available in open access. An R package that implements the model is available (6).

Here we give only an overview of the model, first in its simplest form, and then in the two-sex time-varying version used in our analyses. We recommend that the interested reader consult the published literature for details.

The simple kinship model. In its simplest form, the model analyzes the kinship network shown in Figure S1. This form considers only female kin through female lines of descent, for age-classified populations, with time-invariant rates. The network, is defined relative to a focal individual, referred to as Focal, who is a member of a population with specified mortality and fertility schedules, which apply to all individuals. The survival matrix $\mathbf{U}$ contains survival probabilities on the subdiagonal and zeros elsewhere. The fertility matrix $\mathbf{F}$ contains age-specific fertilities in the first row and zeros elsewhere. The population is characterized by a distribution $\boldsymbol{\pi}$ of the ages of mothers at the birth of children.

The essential aspect of the model is to treat the kin, of any type, of Focal as a population and to project the age structure of that population. The 14 types of kin shown in the network are traditional, but the branches of the network can be extended as far as desired. The model incorporates only biological kin and (at present) does not accommodate step-kin or blended families.

The age structure vector of each type of kin is given by a unique letter (e.g., $\mathbf{a}(x)$ is the age distribution of the daughters at age x of Focal),

Let $\mathbf{k}$ be the age structure vector of an arbitrary kin type ($\mathbf{k}$ does not appear in Figure S1 so it can be used this way). The dynamics of kin are

$$\mathbf{k}(x+1) = \mathbf{U}\mathbf{k}(x) + \boldsymbol{\beta}(x) \quad [1]$$

$$\mathbf{k}(0) = \mathbf{k}_0 \quad [2]$$

The kin at age $x+1$ of Focal are the survivors of the kin at age x plus a vector of new recruits $\boldsymbol{\beta}(x)$. New recruits, if any, come from another kin type (e.g., new granddaughters are the offspring not of granddaughters, but of daughters). In that case

$$\mathbf{k}(x+1) = \mathbf{U}\mathbf{k}(x) + \mathbf{F}\,\mathbf{k}^*(x) \quad [3]$$

where $\mathbf{k}^*$ is the other kin type. Some kin receive no new recruits (e.g., Focal can gain no more older sisters after she is born). In that case

$$\mathbf{k}(x+1) = \mathbf{U}\mathbf{k}(x) + \mathbf{0} \quad [4]$$

Each type of kin requires an initial condition, giving the age structure of that type of kin at the birth of Focal. Some of these initial conditions are zero (e.g., Focal has no daughters at birth). Others are calculated using information from the age distribution of the mothers at the birth of offspring (age at maternity). For example, we know that Focal, at birth, has exactly one living mother. We do not know her age, but we know the distribution of that age, so for mothers ($\mathbf{d}(x)$) we write

$$\mathbf{d}_0 = \boldsymbol{\pi} \quad [5]$$

As is the case with all cohort component projections, this model projects expected values of the kinship network.

The time-varying two-sex kinship model. Our analysis uses the matrix kinship model extended to include both sexes and time varying demographic rates.* We define time-varying male and female rates and the sex ratio at birth

$$\mathbf{U}_{\rm f}(t),\, \mathbf{U}_{\rm m}(t) = \text{period female and male survival matrices at time } t \quad [6]$$

$$\mathbf{F}_{\rm f}(t),\, \mathbf{F}_{\rm m}(t) = \text{period female and male fertility matrices at time } t \quad [7]$$

$$\alpha = \text{proportion males among offspring} \quad [8]$$

$$\bar{\alpha} = 1-\alpha \quad [9]$$

The survival matrices contain age-specific survival probabilities on the subdiagonal and zeros elsewhere. Fertility matrices contain age-specific fertility rates on the first row and zeros elsewhere. Fertility includes both female and male children of female and male parents. We set the proportion of males at birth as $\alpha = 0.5$.

The age distribution of each type of kin is extended to include both males and females:

$$\tilde{\mathbf{k}}(x,t) = \left(\frac{\mathbf{k}_{\rm f}}{\mathbf{k}_{\rm m}}\right)(x,t). \quad [10]$$

*This section is modified from (3) and (4) under the terms of a CC-BY license.

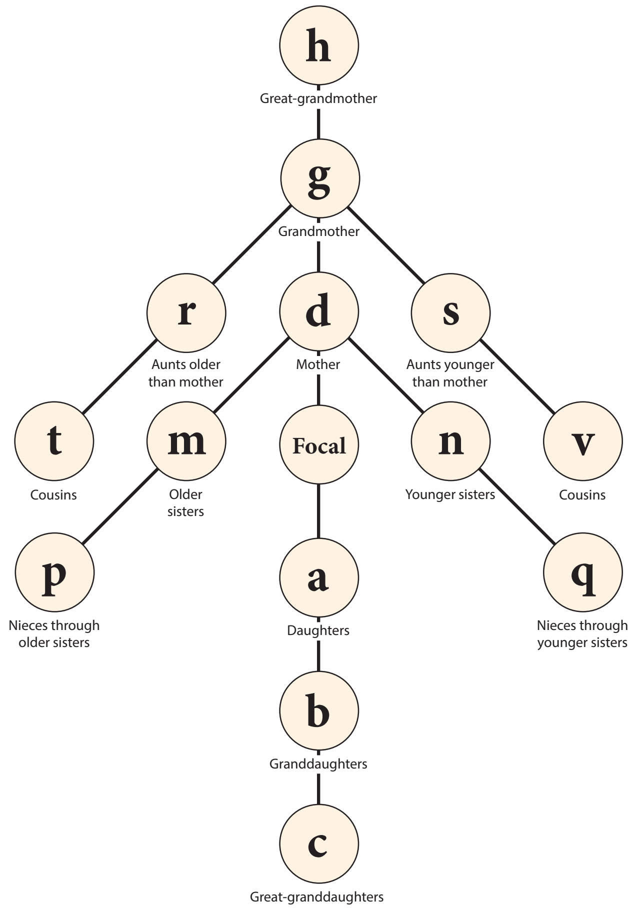

Fig. S1. The kinship network of a Focal individual. Each letter is the age distribution vector of a particular kind of kin. Reproduced from (1) under a CC-BY license.

The tilde denotes block-structured vectors and matrices composed of female and male parts.

The block-structured vector is projected by the time-varying block-structured matrices

$$\tilde{\mathbf{U}}_t = \left(\begin{array}{c|c} \mathbf{U}_\mathrm{f}(t) & \mathbf{0} \\ \hline \mathbf{0} & \mathbf{U}_\mathrm{m}(t) \end{array}\right) \qquad \tilde{\mathbf{F}}_t = \left(\begin{array}{c|c} \bar{\alpha}\mathbf{F}_\mathrm{f}(t) & \bar{\alpha}\mathbf{F}_\mathrm{m}(t) \\ \hline \alpha\mathbf{F}_\mathrm{f}(t) & \bar{\alpha}\mathbf{F}_\mathrm{m}(t) \end{array}\right) \qquad \tilde{\mathbf{F}}^*_t = \left(\begin{array}{c|c} \bar{\alpha}\mathbf{F}_\mathrm{f}(t) & \mathbf{0} \\ \hline \alpha\mathbf{F}_\mathrm{f}(t) & \mathbf{0} \end{array}\right) \tag{11}$$

The dynamics of $\tilde{\mathbf{k}}(x,t)$ are

$$\tilde{\mathbf{k}}(x+1,t+1) \quad = \quad \tilde{\mathbf{U}}_t\tilde{\mathbf{k}}(x,t) + \tilde{\boldsymbol{\beta}}(x,t). \tag{12}$$

The term $\tilde{\boldsymbol{\beta}}(x,t)$ is the recruitment of new kin. It depends on the nature of the kin that provide the new kin.

1. If the recruitment of new kin of type $\mathbf{k}$ is provided by reproduction of a direct ancestor of Focal (parents, grandparents, etc.) then

$$\left(\begin{array}{c} \boldsymbol{\beta}_\mathrm{f} \\ \hline \boldsymbol{\beta}_\mathrm{m} \end{array}\right)(x,t) \quad = \quad \left(\begin{array}{c|c} \bar{\alpha}\mathbf{F}_\mathrm{f}(t) & \mathbf{0} \\ \hline \alpha\mathbf{F}_\mathrm{f}(t) & \mathbf{0} \end{array}\right)\left(\begin{array}{c} \mathbf{k}^*_f \\ \hline \mathbf{k}^*_m \end{array}\right)(x,t) \tag{13}$$

$$\tilde{\boldsymbol{\beta}}(x,t) \quad = \quad \tilde{\mathbf{F}}^*\,\tilde{\mathbf{k}}(x,t) \tag{14}$$

where $\mathbf{k}^*$ denotes the source kin (e.g., parents of Focal are the source of younger siblings of Focal). The matrix $\tilde{\mathbf{F}}^*$ includes reproduction of both female and male offspring by females.

2. If the recruitment is provided by any other kin type, then

$$\left(\begin{array}{c} \boldsymbol{\beta}_f \\ \hline \boldsymbol{\beta}_m \end{array}\right) \quad = \quad \left(\begin{array}{c|c} \bar{\alpha}\mathbf{F}_\mathrm{f}(t) & \bar{\alpha}\mathbf{F}_\mathrm{m}(t) \\ \hline \alpha\mathbf{F}_\mathrm{f}(t) & \bar{\alpha}\mathbf{F}_\mathrm{m}(t) \end{array}\right)\left(\begin{array}{c} \mathbf{k}^*_f \\ \hline \mathbf{k}^*_m \end{array}\right) \tag{15}$$

$$\tilde{\boldsymbol{\beta}}(x,t) \quad = \quad \tilde{\mathbf{F}}\,\tilde{\mathbf{k}}(x,t) \tag{16}$$

where $\tilde{\mathbf{k}}^*(x,t)$ again denotes the source of the recruited kin (e.g, children of Focal are the source of new grandchildren).

3. If there is no recruitment of new kin of type $\mathbf{k}$, then $\tilde{\boldsymbol{\beta}} = \mathbf{0}$.

Our implementation of the kinship models. Our implementation of the kinship models is equivalent to that of (4), with some exceptions that we outline here. First, we assume time-invariant rates and a stable distribution of kin by age before 1950—i.e., the 'time boundary' in (3). This is the first year for which demographic rate data from the 2022 Revision of the World Population Prospects (UNWPP) (7) is available. Second, for computational efficiency, we group ages and years by 5 y groups instead of running single-year models.

Third, we modeled 'flow' births as opposed to 'birth-pulse' populations, meaning that births are distributed along a given age interval (8). We use survival relationships (S) instead of probabilities at exact ages, so that the age-specific fertility rates in ages $(x, x+5)$ prevailing during interval time $(t, t+5)$, become:

$$F_{t,t+5}(x,x+5) = \frac{f_{t,t+5}(x,x+5) + f_{t,t+5}(x+5,x+10)S_{t,t+5}(x,x+5)}{2}\,\frac{L_{t,t+5}(0,5)}{l_0 5} \tag{17}$$

Here, $L_{t,t+5}(0,5)$ are the person-years between birth and age 5 prevailing in the period between t and t+5, and l_0 is the radix of the life table (9). Note that the rates at each time interval $(t, t+5)$ take the value of the intermediate year.

The diagram in Fig. S2 illustrates how our models estimate living kin counts by sex and type of kin for a female Focal. Solid lines in the diagram show which kin is subsidized from another kin (see (1) for a discussion of subsidies in kinship models). As in (4), our model uses female fertility when accounting for sibling relations (i.e., Focal's siblings or Focal's aunts and uncles, who are Focal's parents siblings). This is indicated with a dotted line in Fig. S2.

Additional analyses related to the implementation of the kinship models

Comparing model and empirical kinship data. It has long been recognized that formal kinship models are not expected to reproduce the results of empirical or census measurements (1, 10) because the models describe the kinship network implied by a set of mortality and fertility schedules, whereas census measurements reflect not only mortality and fertility, but many other processes not included in the model. Hence it is useful to compare the model with empirical measurements to assess their differences.

We use registry-based measurements of kinship for Sweden to assess the accuracy of our model-derived estimates of kinship. (11) generated period kin counts for different types of biological relatives for Sweden in the year 2017. To the best of our knowledge this constitutes the only study to present a comprehensive account of kin availability for most of the relatives presented in this study.

In order to evaluate the performance of our kinship models, we compare three different model formulations to the empirical data. 'GKP factors' extrapolate from one-sex models to accounting for both sexes by simple multiplication (e.g., siblings =

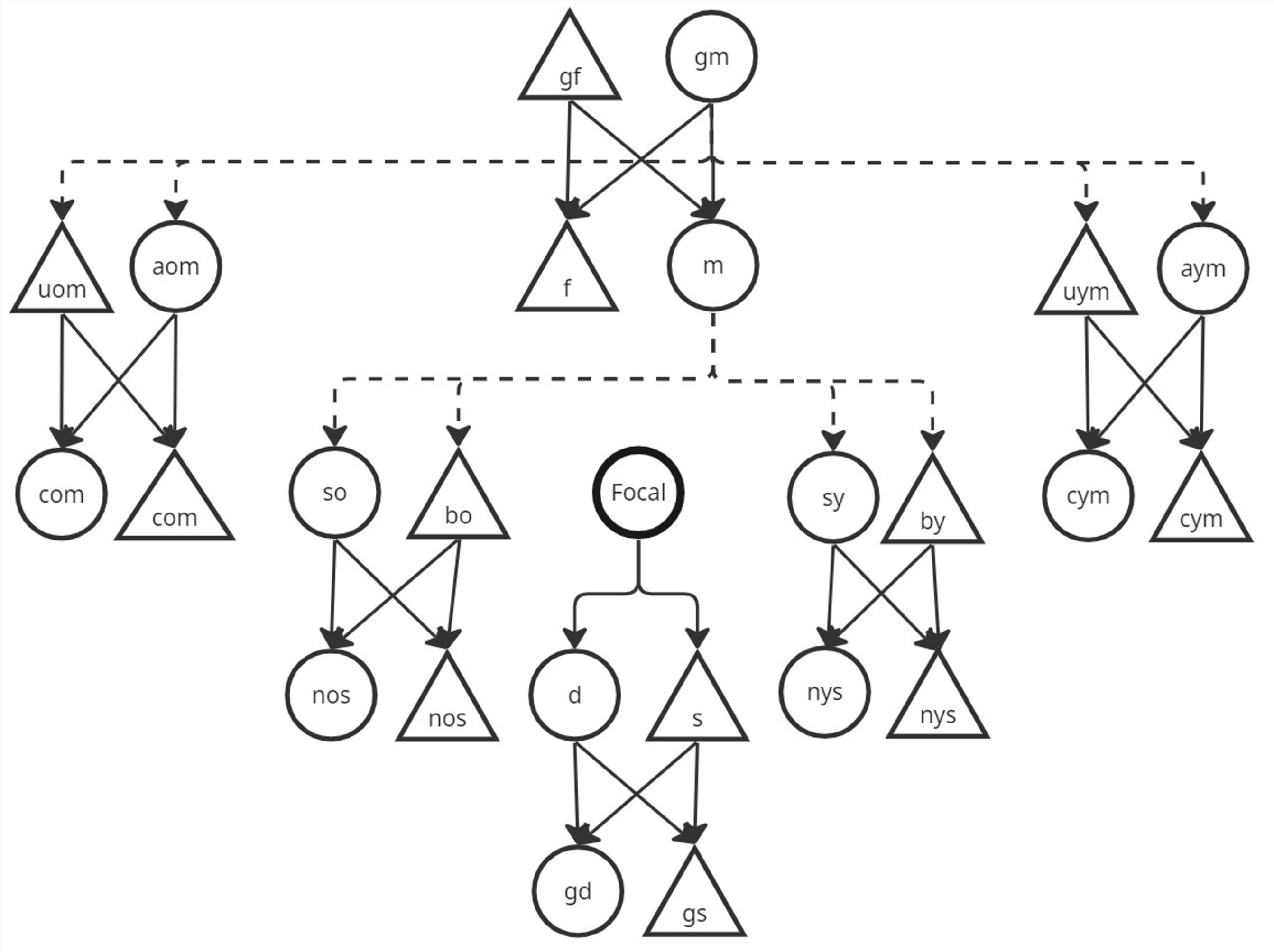

Fig. S2. Kinship diagram showing the different kin ties considered in the two-sex kinship models used for the study (4), excluding greatgrandparents and greatgrandchildren for convenience. Women are circles and men are triangles. Relatives are identified by a unique code: uom = uncles older than mother, uym = uncles younger than mother, com = cousins from uncles/aunts older than mother, ob =older brothers, d = daughters, nys = nieces/nephews (niblings) from younger sisters, gd = grand-daughters, etc. The dotted lines indicate that sibling relations are determined using female (i.e., maternal) fertility.

sisters ×2). 'Full two-sex' models use empirical male and female mortality and fertility data (available for Sweden from the Human Fertility Collection). 'Androgynous fertility' models (the ones we use for the analysis in this project) approximate male fertility by setting it equal to female (4). All models rely on a time-variant implementation of the kinship models, as explained above.

Fig. S3 shows the expected number of kin for the three models alongside the empirical data from (11) for Sweden. The plot shows that our androgynous fertility models were able to capture the expected kin count with a high level of precision for most relative types. Note that the large differences for cousins, grandchildren, niblings and uncles/aunts at older ages of Focal arise from limitations in the empirical data and not with our models. Registry-based estimates are underestimated for these type of kin for some birth cohorts as they information on kin ties for older periods is unavailable–i.e., information on grandparents is needed in order to identify cousins (11).

This exercise supports our assertion that the assumption of pre-1950 demographic stability in the kinship models does not affect our projections of kinship considerably for a country that experienced a post-WWII 'baby boom.' This echoes the findings of previous studies using related methods and data to estimate country-level kinship structure for a number of countries (12, 13).

Exploring the androgynous approximation. As we outlined in the main text, to fully incorporate vital rates of both sexes requires age-specific mortality and fertility schedules for both males and females for all the years of study. Male and female mortality are regularly reported. Male fertility is rarely reported, and is not available for the range of countries and years that we consider here. Thus an approximation to male fertility is needed. (4) discusses several such approximations. The approximation that we adopted uses male and female mortality schedules, and treats male fertility as equal to female fertility; this is called an "androgynous" approximation by (4). Under this scenario, the model provides both male and female kin, and

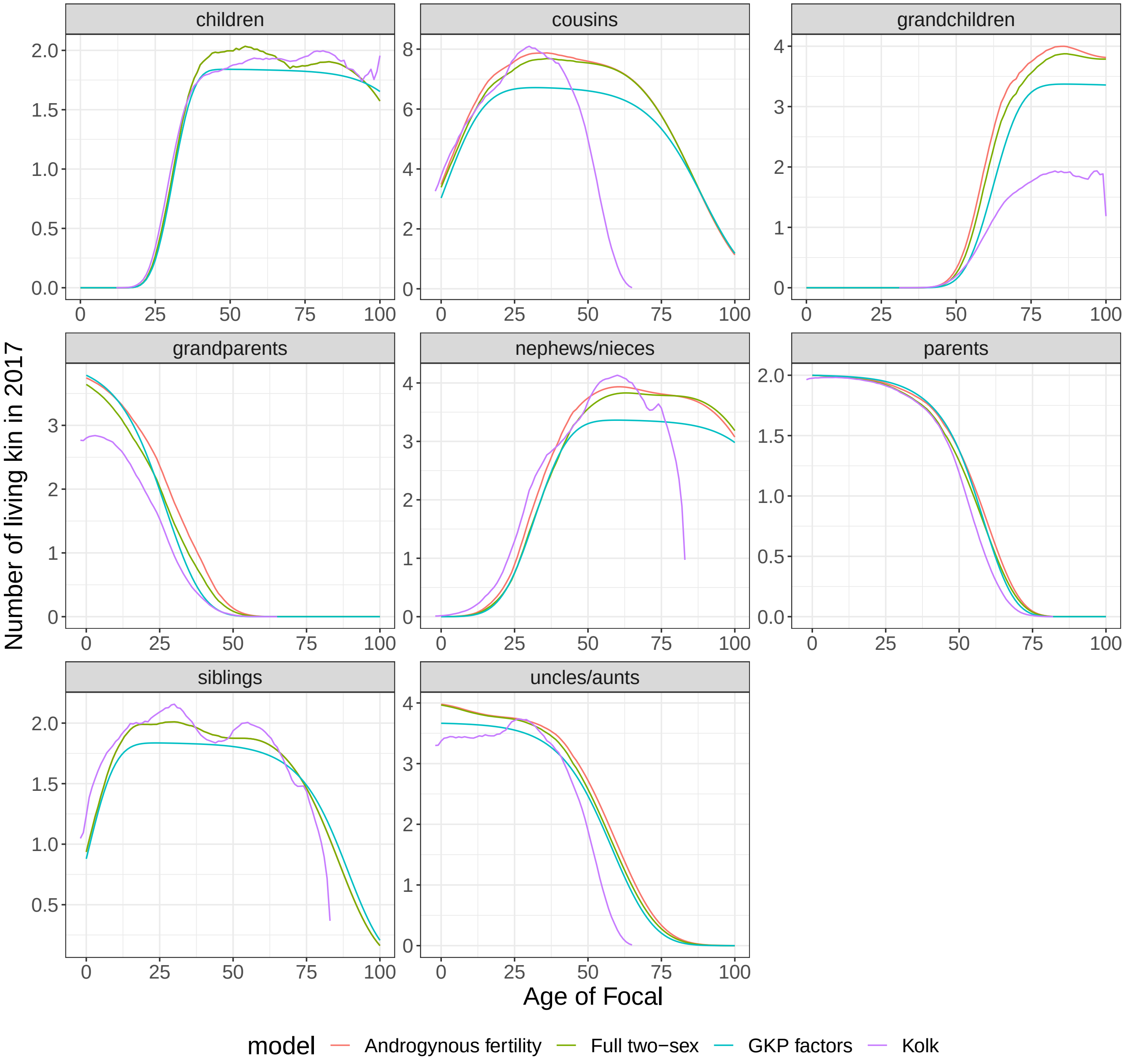

Fig. S3. Comparing kin counts from multiple sources for Sweden in 2017. Estimates labelled as 'Kolk' are registry-based calculations published by (11). The other three models are implemented using matrix kinship models that rely on three different approximations of male fertility, as explained in the text. The estimates in the main text rely on the 'Androgynous fertility' variant.

allows them to experience different survival schedules.

When both male and female fertility schedules are available, it is possible to compare the full two-sex model with the androgynous approximation. (4) compared the numbers of kin at different ages of Focal, between a full two-sex model and a completely androgynous model (female rates used for both mortality and fertility) and found very good agreement even when the actual male and female fertility schedules were dramatically different. Here, we perform a comparative analysis using the same model setup as in Section *Comparing model and empirical kinship data*. To make these results directly comparable to the analysis in the main text, we group survival probabilities, fertility rates, and kin age classes into 5-y groups. All analyses pertain to Sweden.

Overall, the analysis shows a high consistency between the androgynous and the full two-sex model. We start by considering the number of living kin irrespective of the age of kin. Fig. S4 shows the expected number of living kin in 2015 over the life course of Focal (i.e., the expected number of kin irrespective of the age of kin). The results show the both models produce highly comparable estimates of kin. Next, in order to evaluate the magnitude of the discrepancies, we compute the Mean

Absolute Difference (MAD) in the expected kin size by type of kin for 2015. To estimate this measure, we first obtained a vector of all the (absolute) differences between the androgynous and the full-two sex models (i.e., for all the kin types considered, all the ages of kin, and Focal's ages 0, 35, and 65). We then obtained the mean of these differences (in number of kin) and present them by type of kin, averaging over all ages of kin. Table S1 shows that the MAD is smaller than 0.037 relatives for all kin types considered (i.e., the largest mean difference between the benchmark full two-sex models and the approximation provided by the androgynous model is 0.037 relatives). We find the largest discrepancies for a Focal aged 0. This is expected as the age distribution of kin at this age reflects the initial conditions of the model.

This supplementary analysis provides reassuring evidence that the approximation of androgynous fertility provides empirically-plausible kinship distributions, as anticipated by (4). Whereas we find some differences in the expected number of kin between the androgynous and the full two-sex models, these are not large and mainly affect distant ancestors. We emphasize that in an ideal world we would conduct full two-sex models for all countries in the world but male fertility data are only available for a small subset of countries in the global North. Our androgynous approach is the best solution for providing a global picture given the data limitations we face.

Single-year versus abridged matrix kinship models. The kinship model proposed by (1) was implemented initially for single years. In this paper, we use the same model but conduct the analysis on a five-yearly basis (i.e., we use abridged matrix kinship models). We do this to reduce memory usage and computational time. Running single-year models for 230 countries, 101 ages of Focal, 101 ages of kin, 2 sexes, 14 kin types, 72 observed years, and 1000 trajectories for 78 projection years would produce a dataset with around 3.7×10^{14} rows. Here, we show that the abridged model produces results very similar to those of the single-year implementation.

Our abridged analysis is, by definition, less precise than the single-age one. This limitation is similar to the one affecting the classical five-year abridged cohort component method of population projection. We assume that fertility rates are constant within each age group and that cohort changes (i.e., survival) happen linearly. As a result, female exposures can be estimated by taking the average within each interval. Similarly, infant mortality is merged in the survival ratio for newborns, known as S_b in life table notation. The latter is more important for populations with high rates of infant mortality.

Next, we evaluate the degree to which single-year and abridged kinship models produce equivalent estimates. For this, we apply both model sets to China in a female time-variant framework and consider differences in kin counts by kin type at different ages of Focal. We use the published UNWPP median trajectory for each demographic component (7).

Figures S5, S6 and S7 show that, on the whole, both models are highly consistent. The larger inconsistencies, in relative terms, are present when kin counts are very small, indicating that a given relative type is fairly uncommon at a given age of Focal. For example, we find relatively large discrepancies for the number of great-grandmothers for a focal aged 65. This is expected given the very small counts for these kin-age combinations and is not a major reason for concern. Overall, the fit between the two implementations improves over time, as we can see for the case of cousins, sisters and nieces for a Focal aged 0. Great-grandmothers are an exception: we document a discrepancy between the models for great-grandmothers for a focal aged 0. This is partly derived from the fact that the model assumes an initial stable distribution of mothers, grandparents and great-grandparents (i.e., the 'age-boundary'), leading to a small over-estimation of these kin types relative to the single-year model.

Regional groupings

We group territories into regions as defined by the 2022 Revision of the World Population Prospects (UNWPP)[†]. These are:

Africa

Algeria; Cote d'Ivoire; Angola; Benin; Botswana; Burkina Faso; Reunion; Burundi; Cabo Verde; Cameroon; Central African Republic; Chad; Comoros; Congo; Democratic Republic of the Congo; Djibouti; Egypt; Equatorial Guinea; Eritrea; Eswatini; Ethiopia; Gabon; Gambia; Ghana; Guinea; Guinea-Bissau; Kenya; Lesotho; Liberia; Libya; Madagascar; Malawi; Mali; Mauritania; Mauritius; Mayotte; Morocco; Mozambique; Namibia; Niger; Nigeria; Rwanda; Saint Helena; Sao Tome and Principe; Senegal; Seychelles; Sierra Leone; Somalia; South Africa; South Sudan; Sudan; Togo; Tunisia; Uganda; United Republic of Tanzania; Western Sahara; Zambia; Zimbabwe.

Asia

Turkiye; Afghanistan; Armenia; Azerbaijan; Bahrain; Bangladesh; Bhutan; Brunei Darussalam; Cambodia; China; Hong Kong SAR; Macao SAR; Taiwan, China; Cyprus; Dem. People's Republic of Korea; Georgia; India; Indonesia; Iran (Islamic Republic of); Iraq; Israel; Japan; Jordan; Kazakhstan; Kuwait; Kyrgyzstan; Lao People's Democratic Republic; Lebanon; Malaysia; Maldives; Mongolia; Myanmar; Nepal; Oman; Pakistan; Philippines; Qatar; Republic of Korea; Saudi Arabia; Singapore; Sri Lanka; State of Palestine; Syrian Arab Republic; Tajikistan; Thailand; Timor-Leste; Turkmenistan; United Arab Emirates; Uzbekistan; Viet Nam; Yemen.

Europe

Albania; Andorra; Austria; Belarus; Belgium; Bosnia and Herzegovina; Bulgaria; Croatia; Czechia; Denmark; Estonia; Faroe Islands; Finland; France; Germany; Gibraltar; Greece; Guernsey; Holy See; Hungary; Iceland; Ireland; Isle of Man; Italy; Jersey; Kosovo (under UNSC res. 1244); Latvia; Liechtenstein; Lithuania; Luxembourg; Malta; Monaco; Montenegro; Netherlands; North Macedonia; Norway; Poland; Portugal; Republic of Moldova; Romania; Russian Federation; San Marino; Serbia; Slovakia; Slovenia; Spain; Sweden; Switzerland; Ukraine; United Kingdom.

[†] https://population.un.org/wpp/, accessed 12 Jan 2023

Table S1. Mean absolute difference (MAD) in the expected number of kin between the androgynous fertility and the full two-sex models for 2015 Sweden The MAD considers all differences between male and female kin by type of kin.

	Age of Focal		
Kin type	0	35	65
Aunts	0.009	0.008	0.003
Uncles	0.009	0.008	0.003
Daughters	0.000	0.000	0.000
Sons	0.000	0.000	0.000
Grandmothers	0.011	0.006	0.000
Grandfathers	0.033	0.013	0.000
Great-grandmothers	0.023	0.003	0.000
Great-grandfathers	0.037	0.003	0.000
Mother	0.000	0.000	0.000
Father	0.015	0.015	0.003
Sisters	0.000	0.000	0.000
Brothers	0.000	0.000	0.000

Latin America and the Caribbean

Anguilla; Saint Bartholemy; Antigua and Barbuda; Argentina; Aruba; Bahamas; Barbados; Belize; Bolivia (Plurinational State of); Bonaire, Sint Eustatius and Saba; Brazil; British Virgin Islands; Cayman Islands; Chile; Colombia; Costa Rica; Cuba; Dominica; Dominican Republic; Ecuador; El Salvador; Falkland Islands (Malvinas); French Guiana; Grenada; Guadeloupe; Guatemala; Guyana; Haiti; Honduras; Jamaica; Martinique; Mexico; Montserrat; Nicaragua; Panama; Paraguay; Peru; Puerto Rico; Saint Kitts and Nevis; Saint Lucia; Saint Martin (French part); Saint Vincent and the Grenadines; Sint Maarten (Dutch part); Suriname; Trinidad and Tobago; Turks and Caicos Islands; United States Virgin Islands; Uruguay; Venezuela (Bolivarian Republic of); Curacao.

Northern America

Bermuda; Canada; Greenland; Saint Pierre and Miquelon; United States of America.

Oceania

American Samoa; Australia; Cook Islands; Fiji; French Polynesia; Guam; Kiribati; Marshall Islands; Micronesia (Fed. States of); Nauru; New Caledonia; New Zealand; Niue; Northern Mariana Islands; Palau; Papua New Guinea; Samoa; Solomon Islands; Tokelau; Tonga; Tuvalu; Vanuatu; Wallis and Futuna Islands.

Excluded territories Given data limitations, we exclude the following territories from our analysis: Channel Islands, Holy See, Pitcairn, and Sark, Wallis, and Futuna Islands.

Extended data visualisations

In this section, we present additional visualisations to complement the graphics in the main manuscript. The results presented in this section pertain to:

- Regional estimates of total family size (Figs. S8-S9)
- Regional estimates of horizontal and vertical family size (Figs. S10-S12)
- Average number of kin in five selected countries (Figs. S13-S14)
- Kin composition of family networks (Figs. S15-S20)

Tables with country-level kin counts

In this section, we provide three tables with detailed country-level estimates of kinship structure. The tables are given for a Focal aged 0-4, 35-39, and 65-69, respectively. Countries are identified by a unique three-letter ISO code. Each table presents the estimates for selected type of kin for three years: 1950-1955, 2020-2025, and 2095-2100. Note that projected data only shows the median value of the 1000 country-level projections. Values in each table are expected kin counts, as in the main manuscript. Full results, by type of kin, country, year, age of Focal, and age of kin, including median and lower and upper 80% projection intervals, are available at the Harvard Dataverse (14).

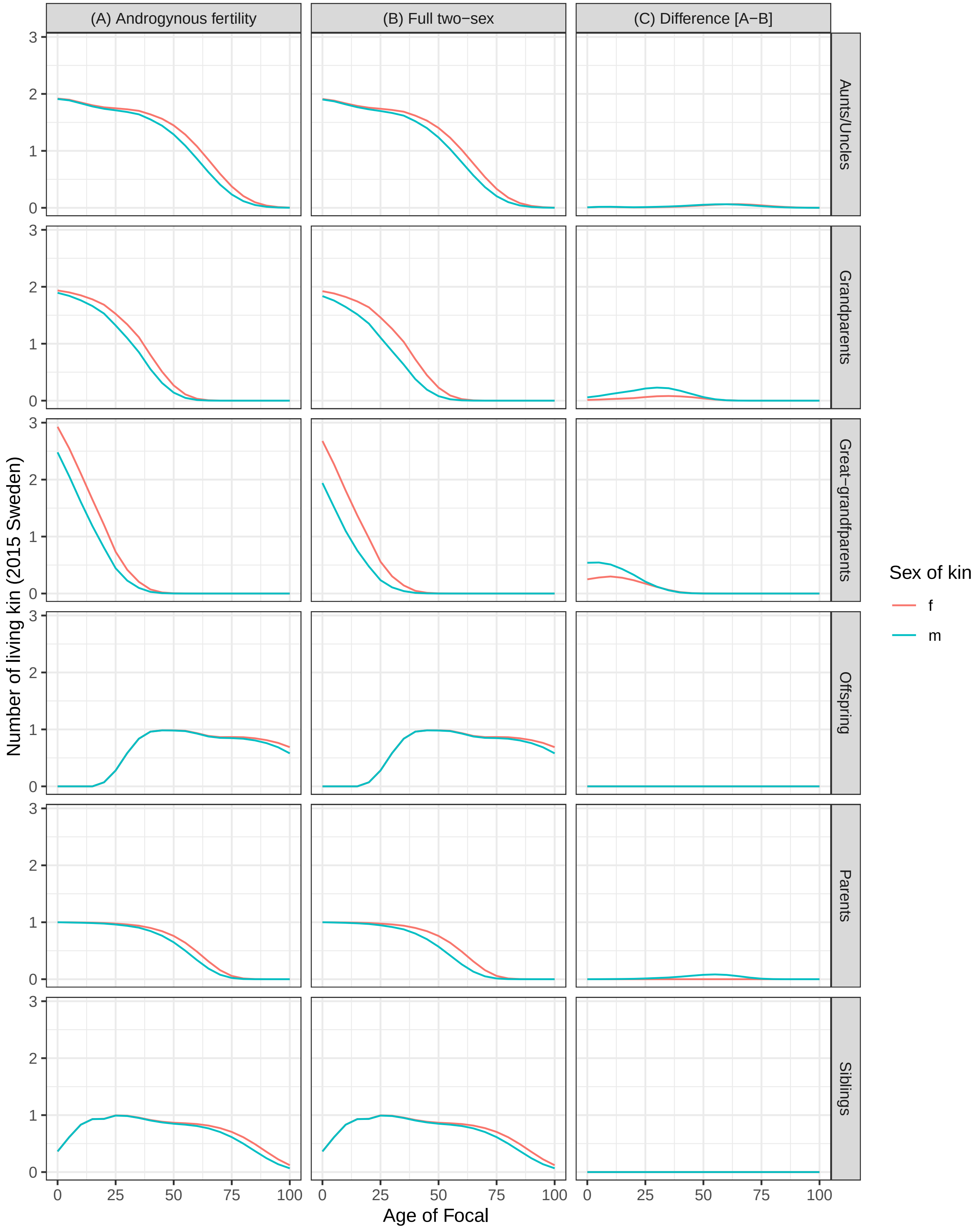

Fig. S4. Expected number of kin over the life course of Focal in 2015 Sweden by sex of kin (results presented by 5y groups, as in the main text). Column A: Models assume that male fertility is equivalent to female fertility (i.e., 'androgynous fertility'). Column B: Models use real male and female fertility ('full two-sex'). Column C: Absolute difference between expected number of living kin (Column A - Column B).

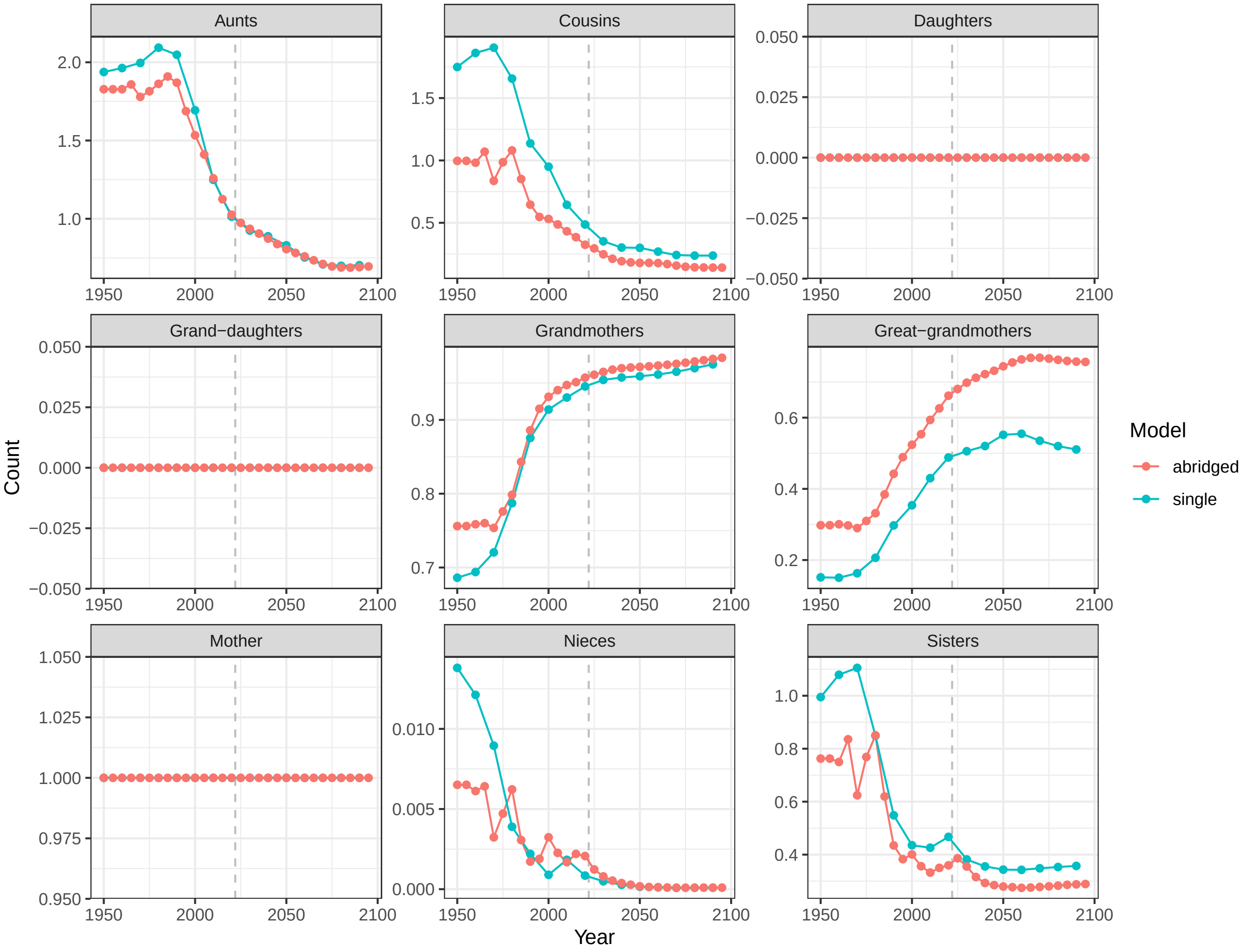

Fig. S5. Comparative number of female living kin for a Chinese Focal at age class 0 (i.e., age 0 for single-year models and age 0-4 for abridged models). Single-year estimates are shown every 10 years.

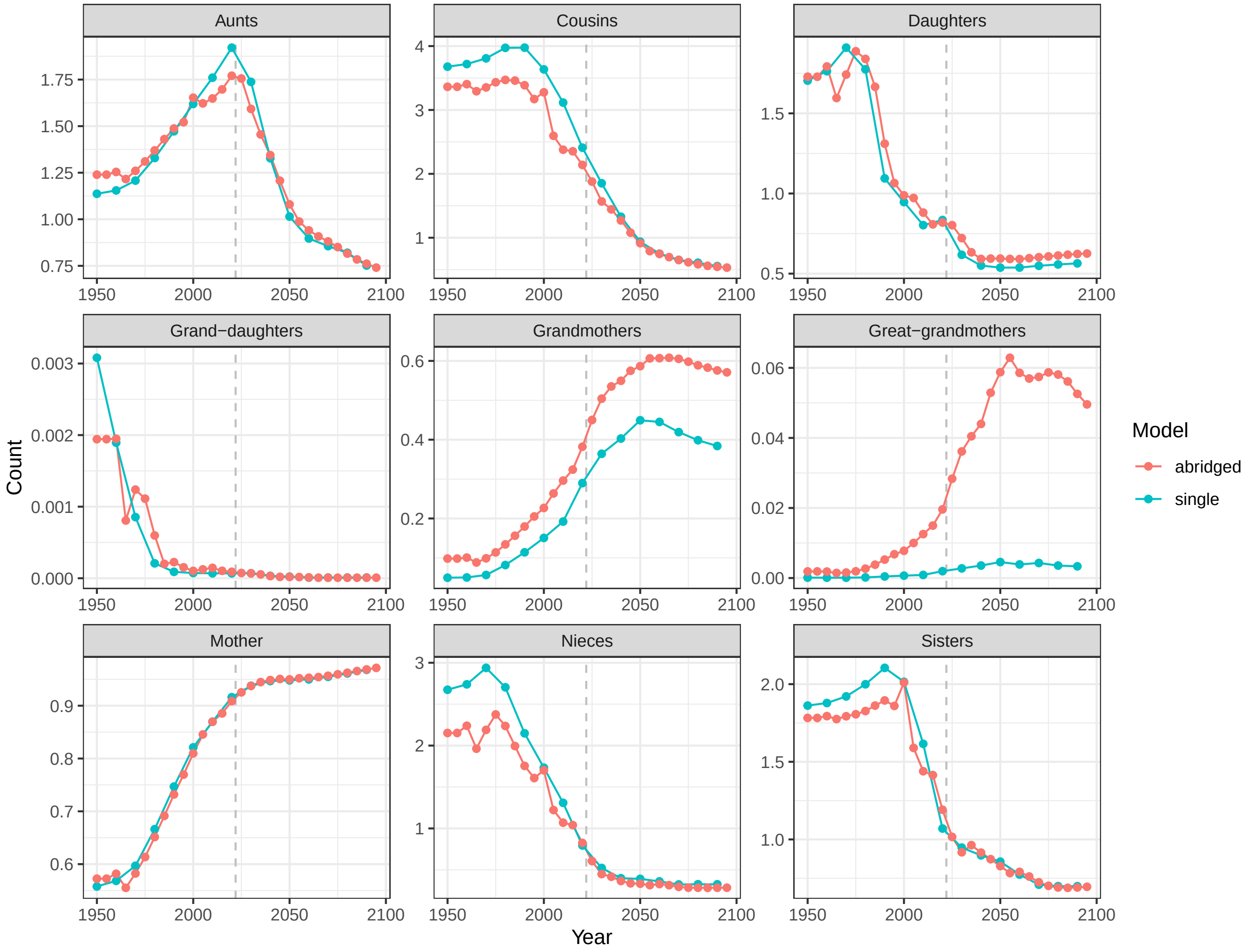

Fig. S6. Comparative number of female living kin for a Chinese Focal at age class 35 (i.e., age 35 for single-year models and age 35-39 for abridged models). Single-year estimates are shown every 10 years.

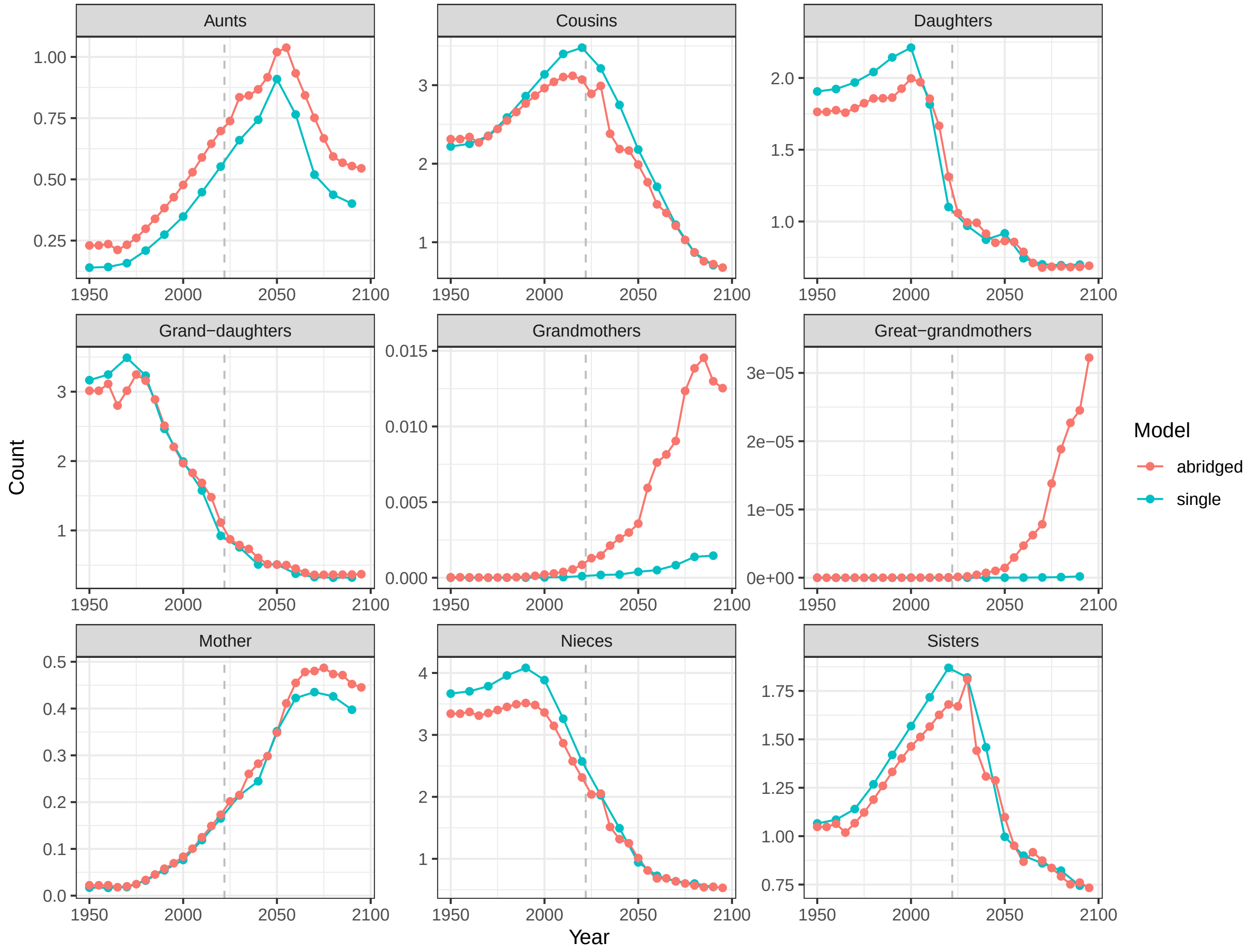

Fig. S7. Comparative number of female living kin for a Chinese Focal at age class 65 (i.e., age 65 for single-year models and age 65-69 for abridged models). Single-year estimates are shown every 10 years.

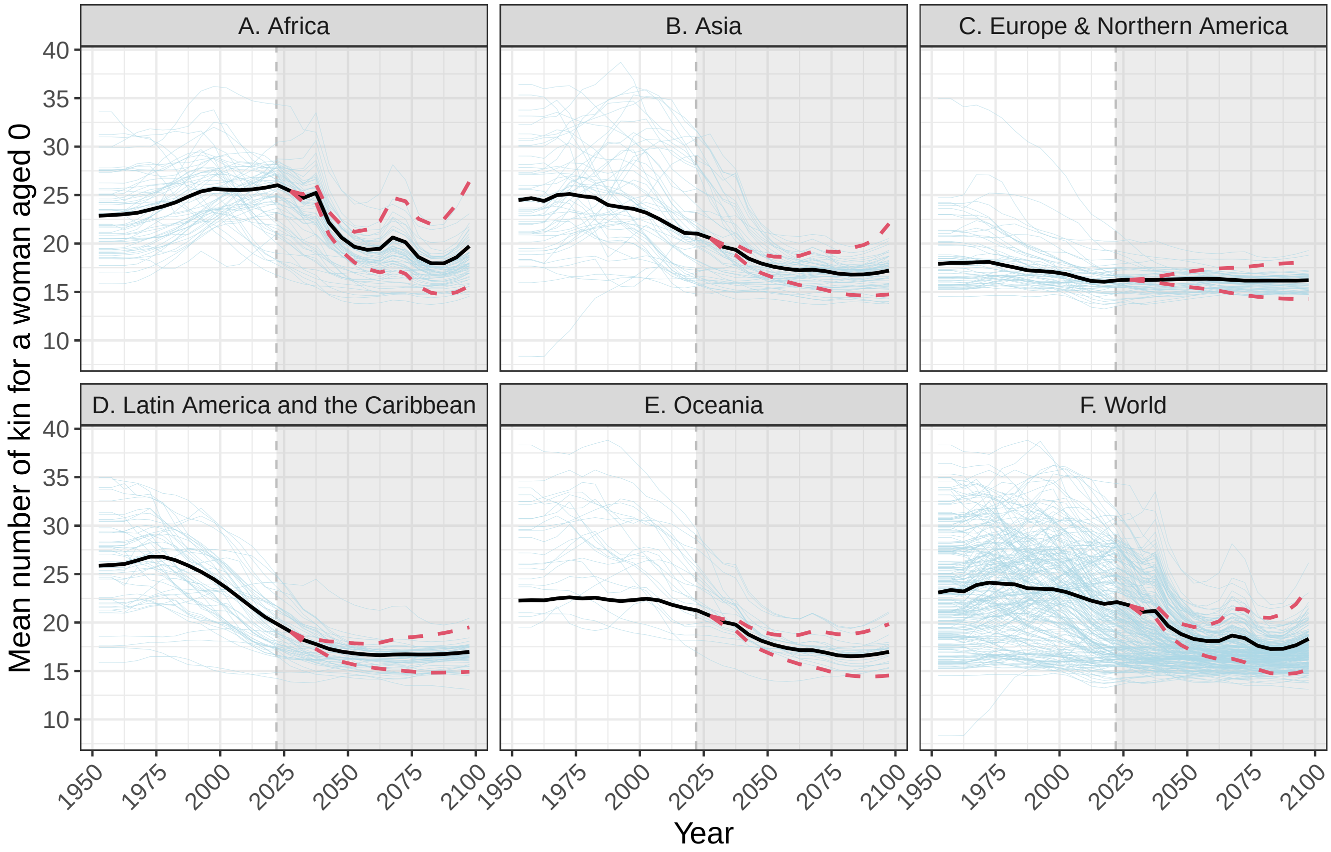

Fig. S8. Total family size (the sum of all living great-grandparents, grandparents, parents, children, grandchildren, great-grandchildren, aunts/uncles, niblings, siblings, and cousins) for a 0 yo female Focal in different world regions (Panels A-E) and globally (Panel F). Light-blue lines in the background show country-level values (median projection trajectory after 2021). The thick black line shows the regional values; after 2021, the black lines show the projection median and the red lines represent the 80% projection intervals for each region (averages weighted by country-level population size at Focal's age 0).

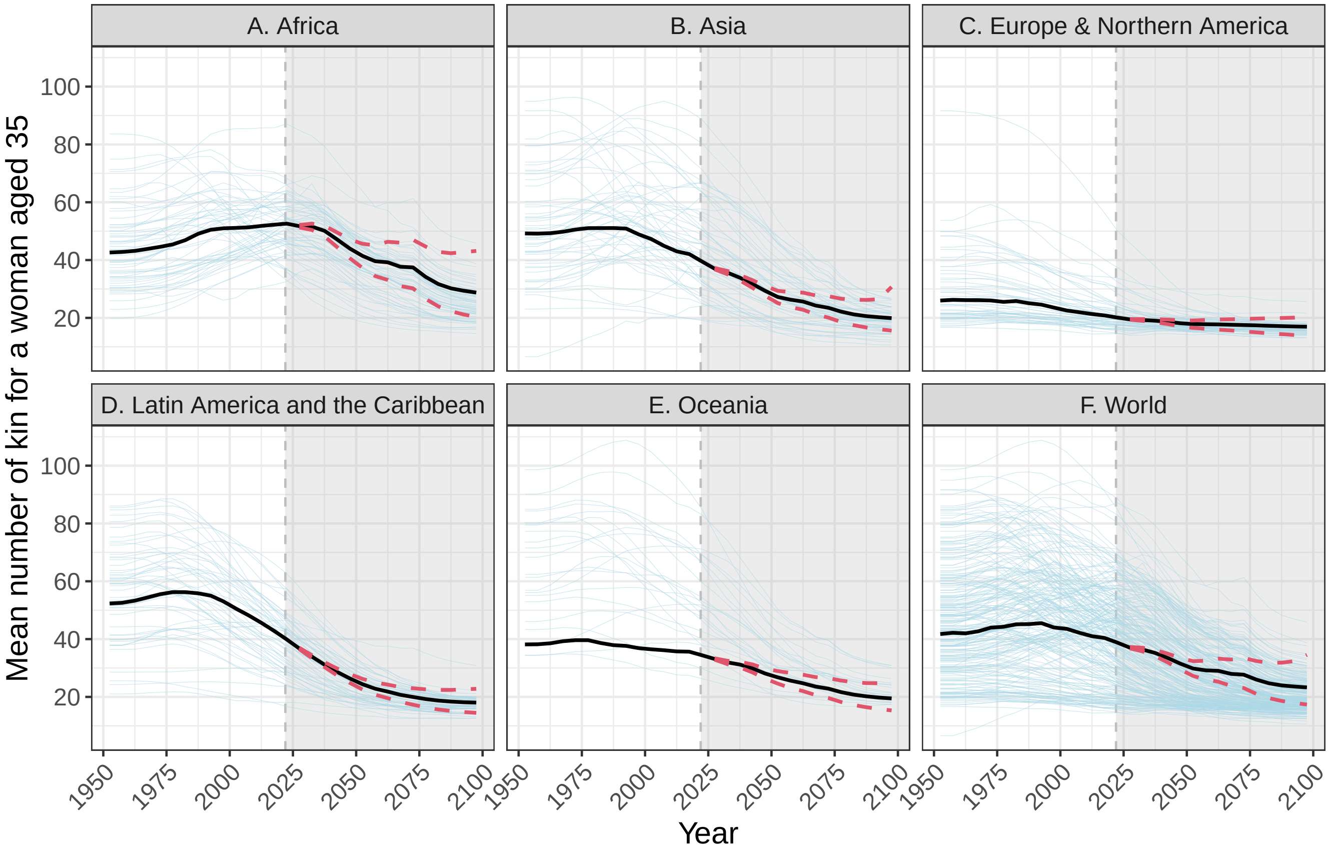

Fig. S9. Total family size (the sum of all living great-grandparents, grandparents, parents, children, grandchildren, great-grandchildren, aunts/uncles, niblings, siblings, and cousins) for a 35 yo female Focal in different world regions (Panels A-E) and globally (Panel F). Light-blue lines in the background show country-level values (median projection trajectory after 2021). The thick black line shows the regional values; after 2021, the black lines show the projection median and the red lines represent the 80% projection intervals for each region (averages weighted by country-level population size at Focal's age 35).

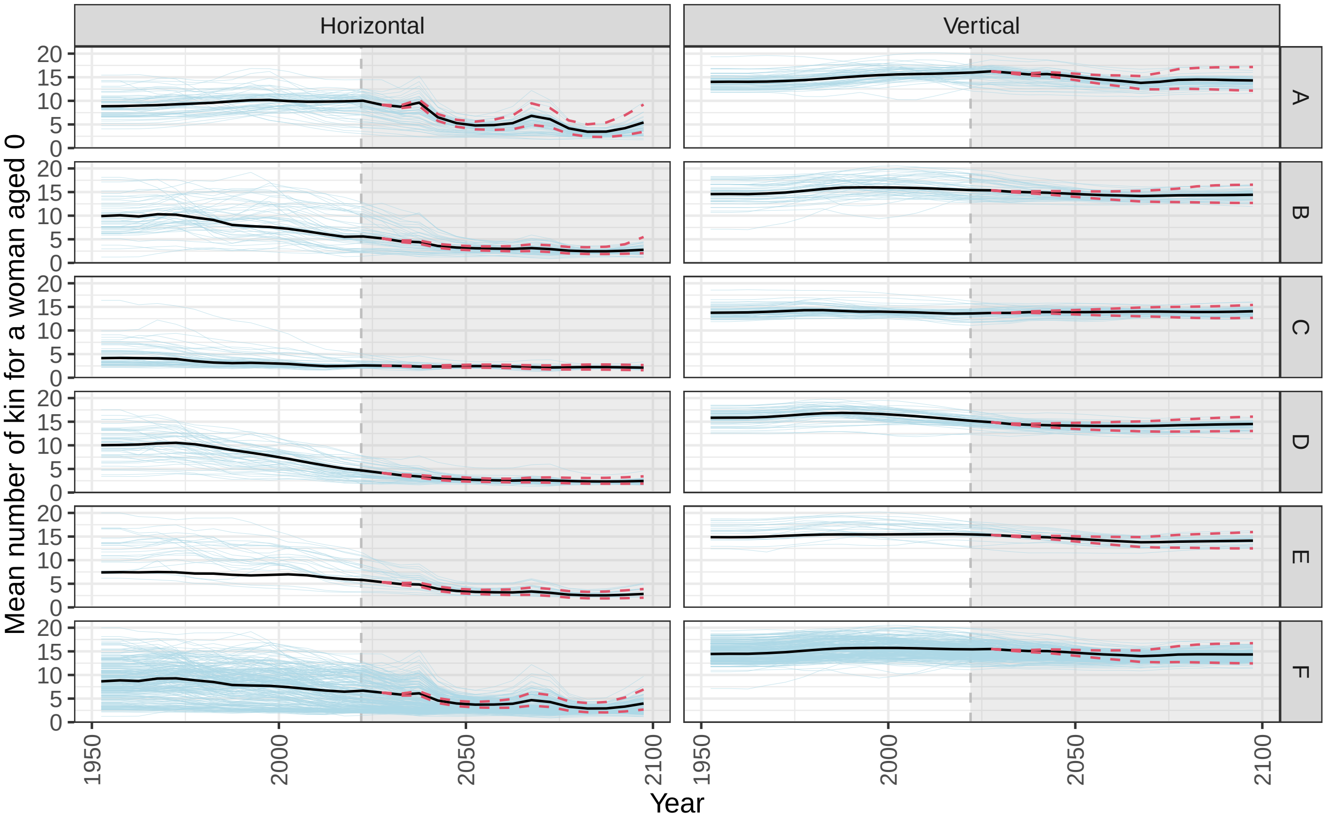

Fig. S10. Family size by horizontal (great-grandparents, grandparents, parents, children, grandchildren, and great-grandchildren) and vertical (aunts/uncles, niblings, siblings, and cousins) family type for a 0 yo female Focal. Regions are: A = Africa, B = Asia, C = Europe and Northern America, D = Latin America and the Caribbean, E = Oceania, F. World. Light-blue lines in the background show country-level values (median projection trajectory after 2021). The thick black line shows the regional values; after 2021, the black lines show the projection median and the red lines represent the 80% projection intervals for each region (averages weighted by country-level population size at Focal's age 0).

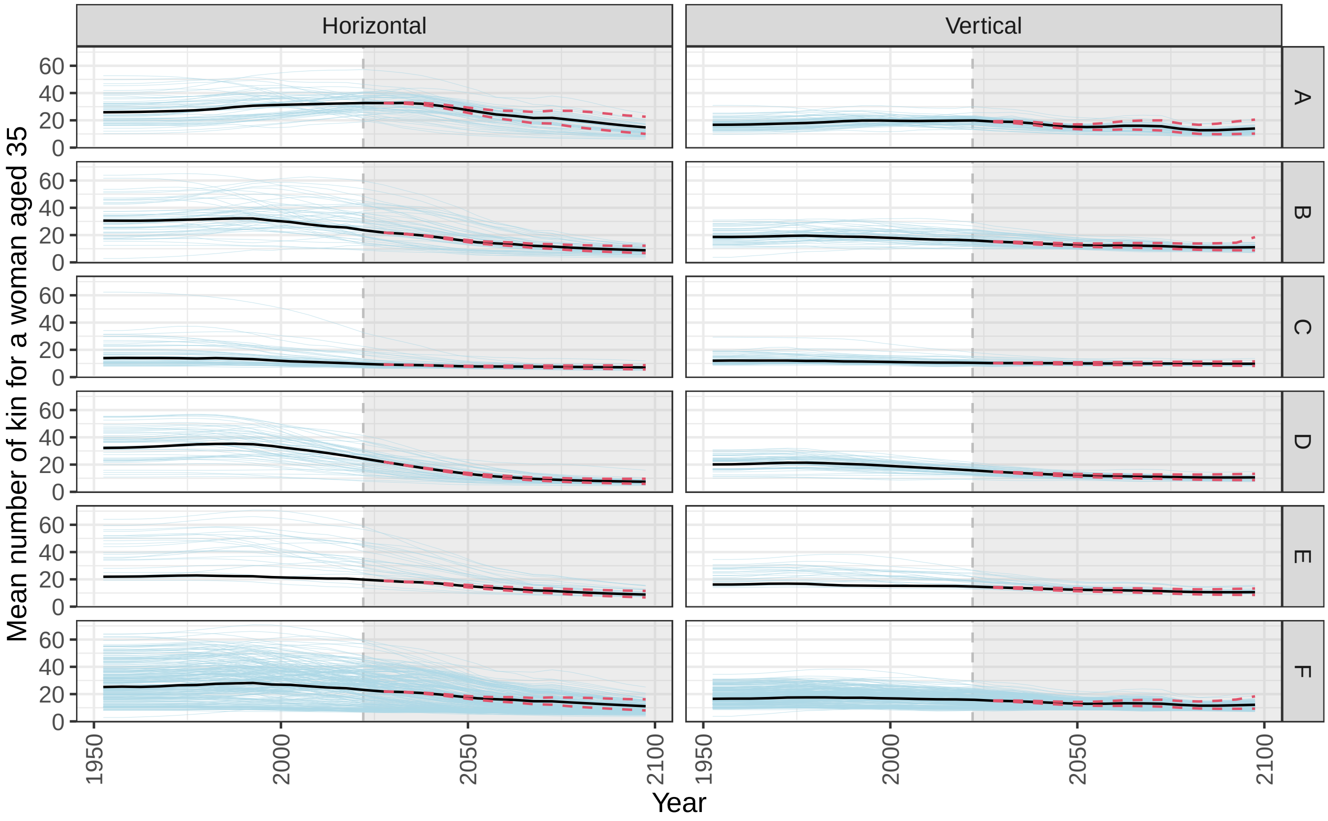

Fig. S11. Family size by horizontal (great-grandparents, grandparents, parents, children, grandchildren, and great-grandchildren) and vertical (aunts/uncles, niblings, siblings, and cousins) family type for a 35 yo female Focal. Regions are: A = Africa, B = Asia, C = Europe and Northern America, D = Latin America and the Caribbean, E = Oceania, F. World. Light-blue lines in the background show country-level values (median projection trajectory after 2021). The thick black line shows the regional values; after 2021, the black lines show the projection median and the red lines represent the 80% projection intervals for each region (averages weighted by country-level population size at Focal's age 35).

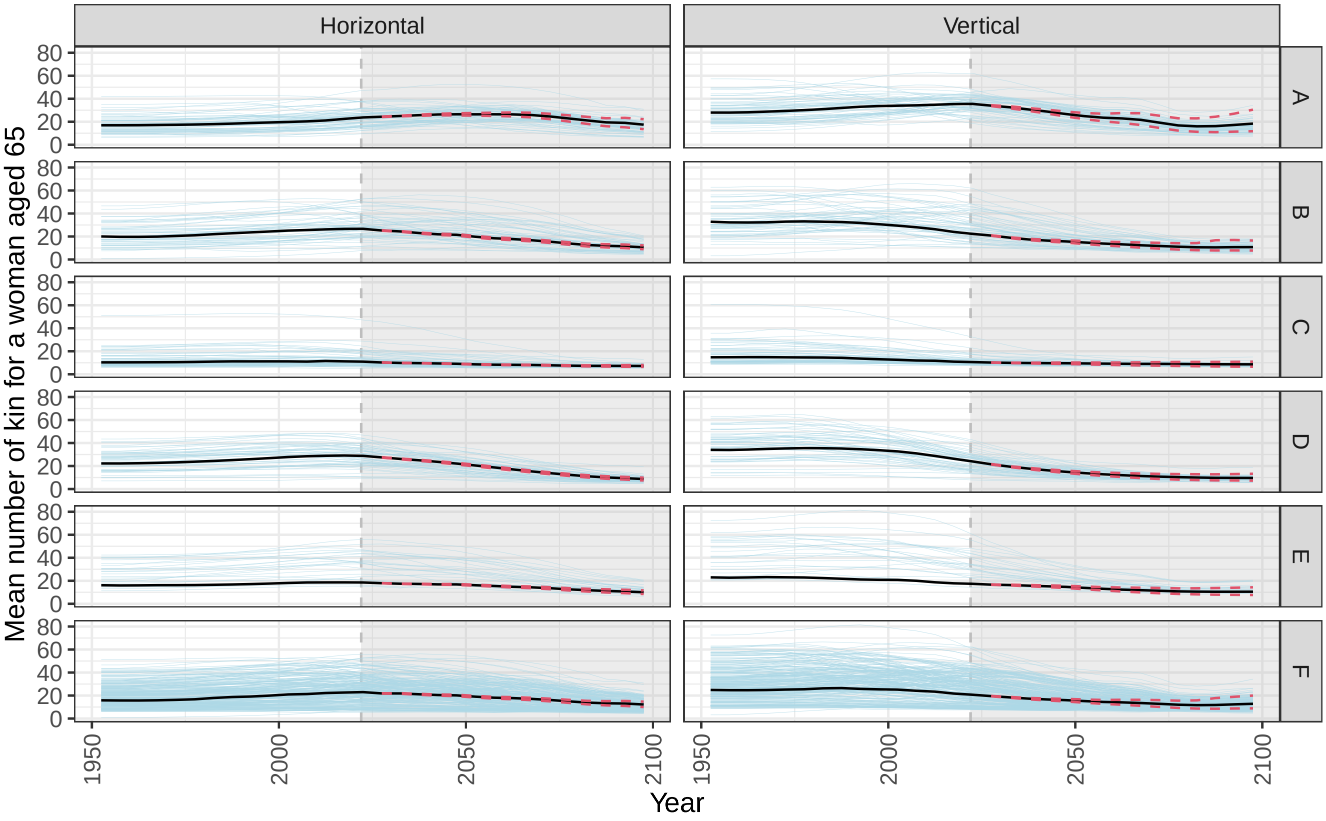

Fig. S12. Family size by horizontal (great-grandparents, grandparents, parents, children, grandchildren, and great-grandchildren) and vertical (aunts/uncles, niblings, siblings, and cousins) family type for a 65 yo female Focal. Regions are: A = Africa, B = Asia, C = Europe and Northern America, D = Latin America and the Caribbean, E = Oceania, F. World. Light-blue lines in the background show country-level values (median projection trajectory after 2021). The thick black line shows the regional values; after 2021, the black lines show the projection median and the red lines represent the 80% projection intervals for each region (averages weighted by country-level population size at Focal's age 65).

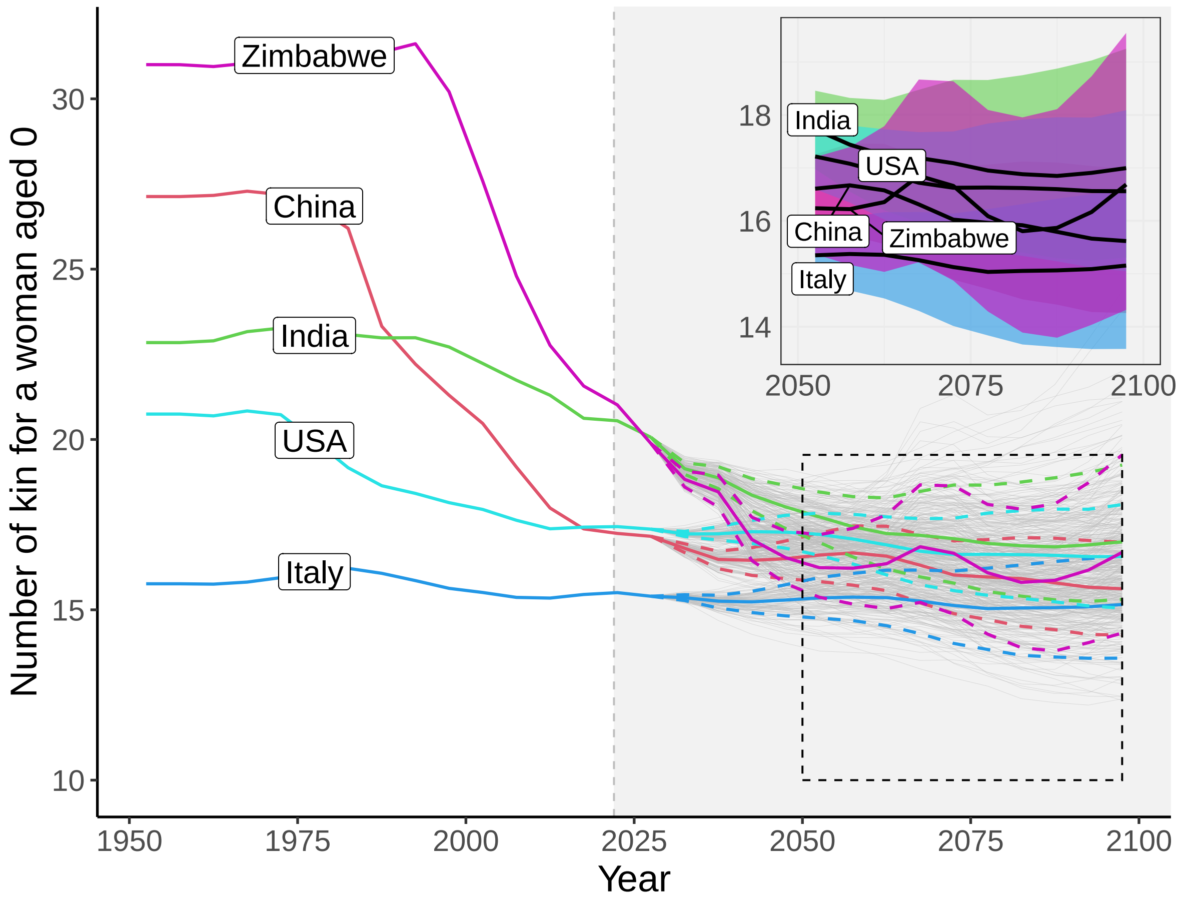

Fig. S13. Average number of living kin for a Focal woman aged 0 in selected countries. Grey lines in background after 2021 show 100 trajectories from the 2022 Revision of the United Nations World Population Prospects. The continuous lines show the median of these projections and the dashed lines indicate the 80% projection intervals.

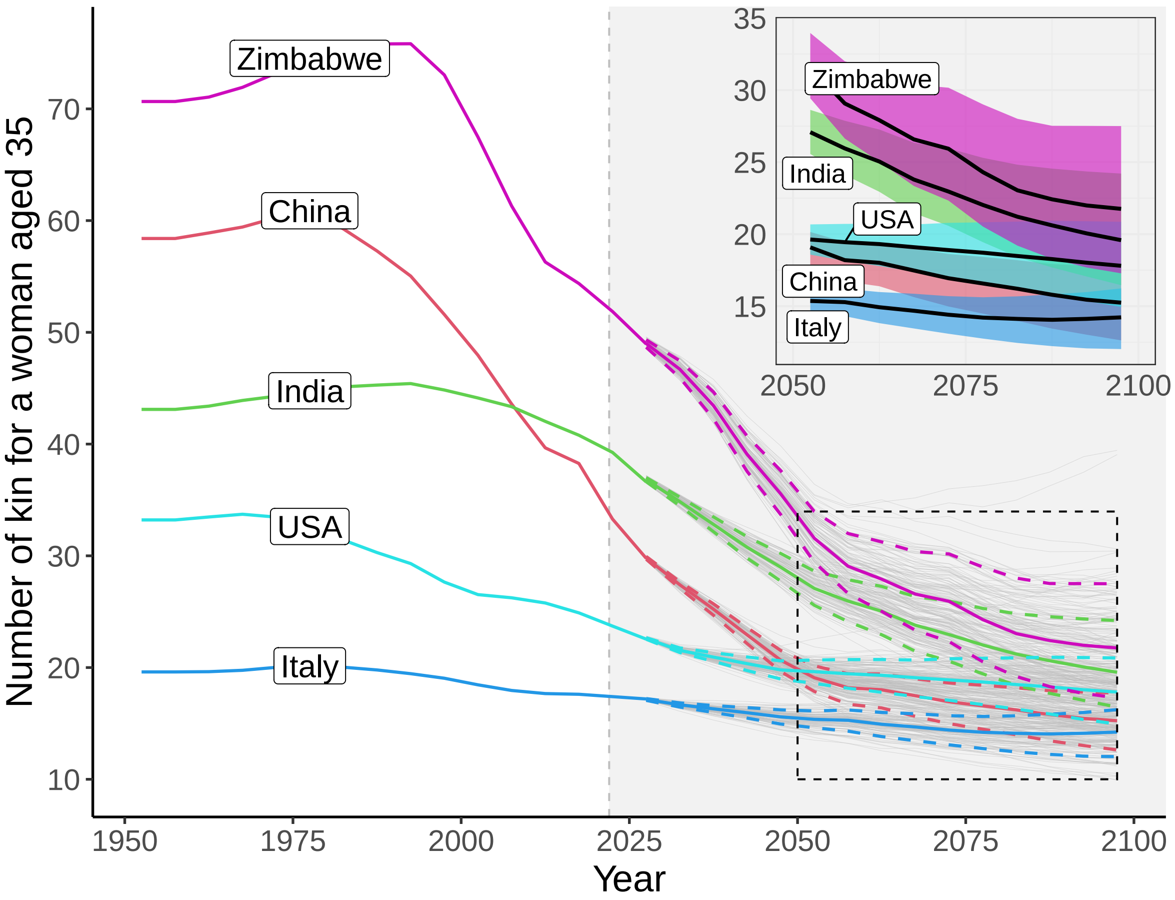

Fig. S14. Average number of living kin for a Focal woman aged 35 in selected countries. Grey lines in background after 2021 show 100 trajectories from the 2022 Revision of the United Nations World Population Prospects. The continuous lines show the median of these projections and the dashed lines indicate the 80% projection intervals.

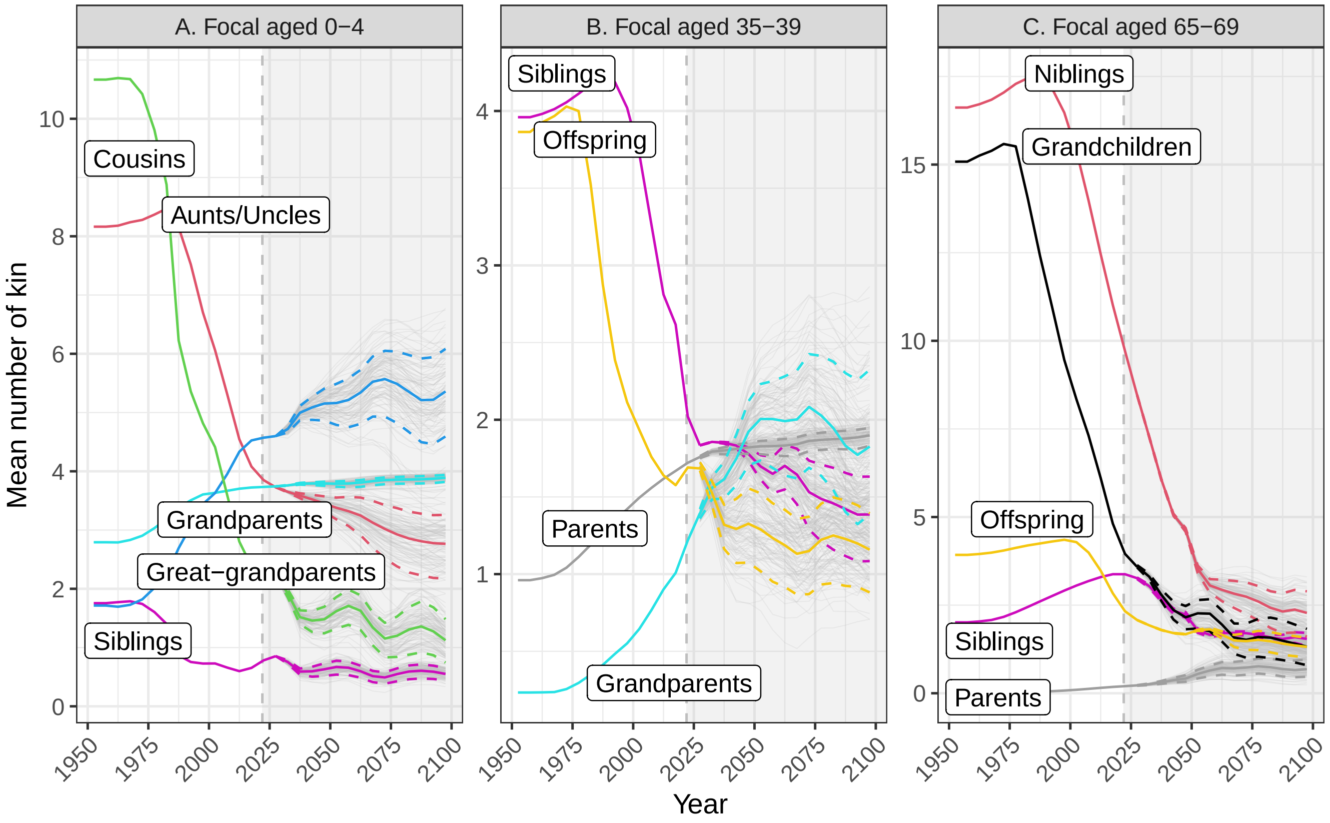

Fig. S15. Expected number of selected kin for a newborn woman in China (Panel A), a Focal aged 35 (Panel B), and a Focal aged 65 (Panel C). Grey lines in background after 2021 show 100 trajectories estimated using probabilistic trajectories from the 2022 Revision of the United Nations World Population Prospects. Thick lines indicate the median estimates and dashed lines show the 80% projection intervals.

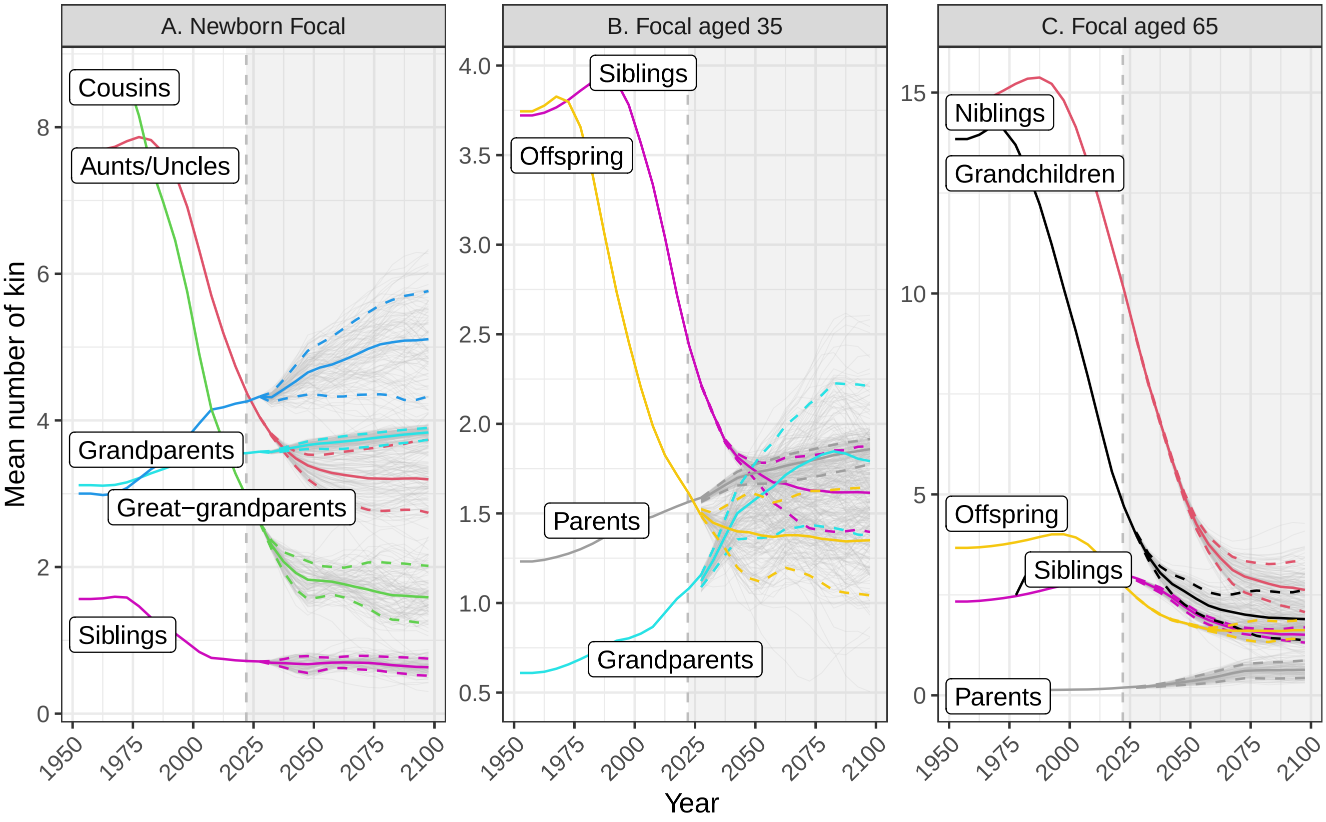

Fig. S16. Expected number of selected kin for a newborn woman in Brazil (Panel A), a Focal aged 35 (Panel B), and a Focal aged 65 (Panel C). Grey lines in background after 2021 show 100 trajectories estimated using probabilistic trajectories from the 2022 Revision of the United Nations World Population Prospects. Thick lines indicate the median estimates and dashed lines show the 80% projection intervals.

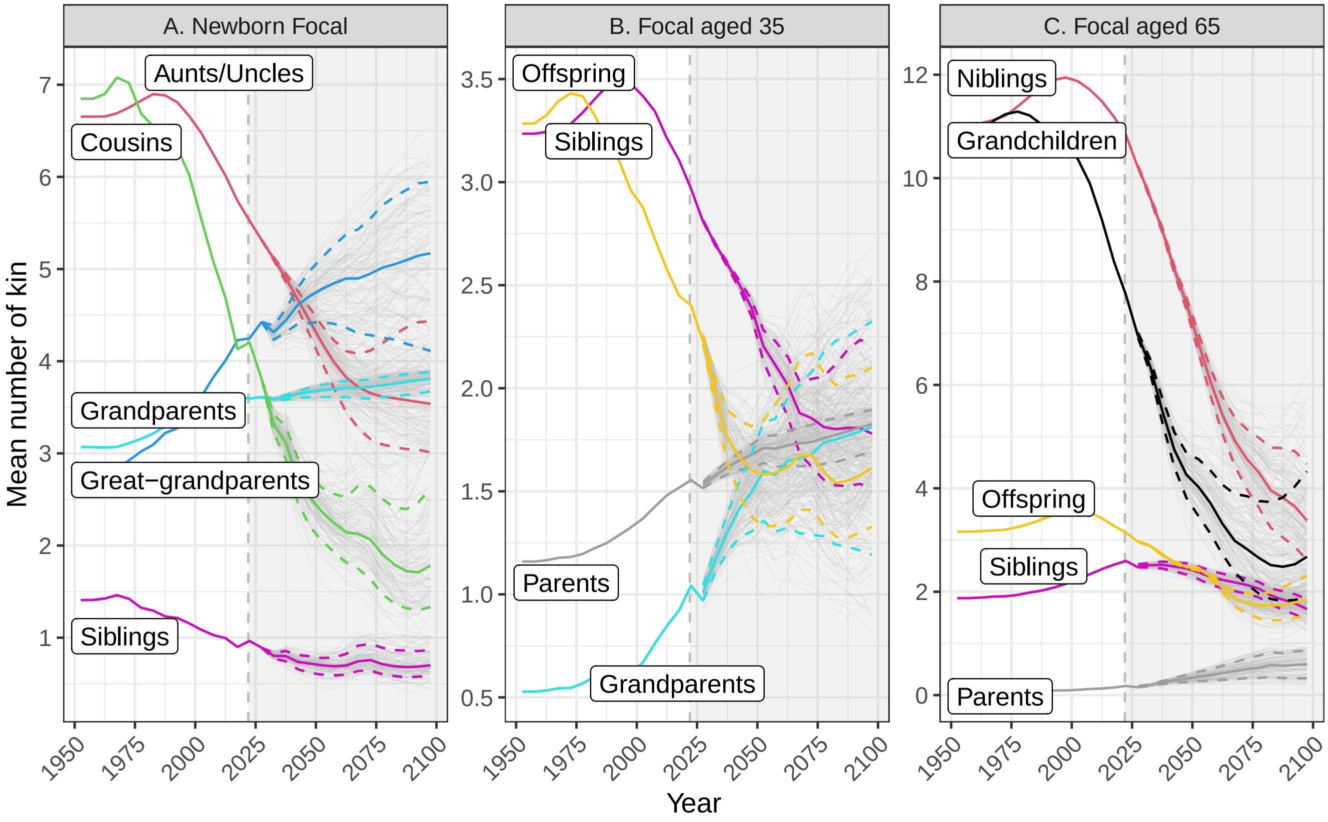

Fig. S17. Expected number of selected kin for a newborn woman in India (Panel A), a Focal aged 35 (Panel B), and a Focal aged 65 (Panel C). Grey lines in background after 2021 show 100 trajectories estimated using probabilistic trajectories from the 2022 Revision of the United Nations World Population Prospects. Thick lines indicate the median estimates and dashed lines show the 80% projection intervals.

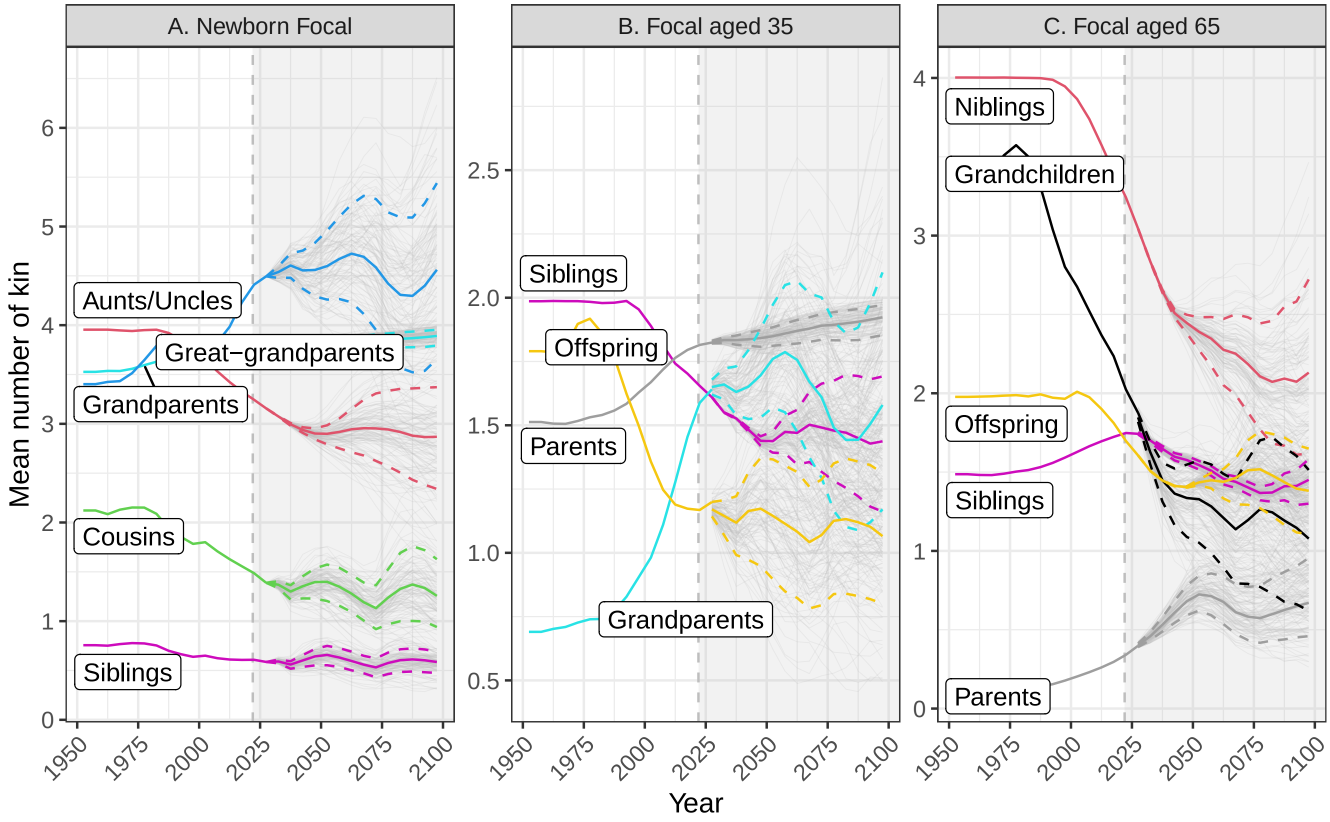

Fig. S18. Expected number of selected kin for a newborn woman in Italy (Panel A), a Focal aged 35 (Panel B), and a Focal aged 65 (Panel C). Grey lines in background after 2021 show 100 trajectories estimated using probabilistic trajectories from the 2022 Revision of the United Nations World Population Prospects. Thick lines indicate the median estimates and dashed lines show the 80% projection intervals.

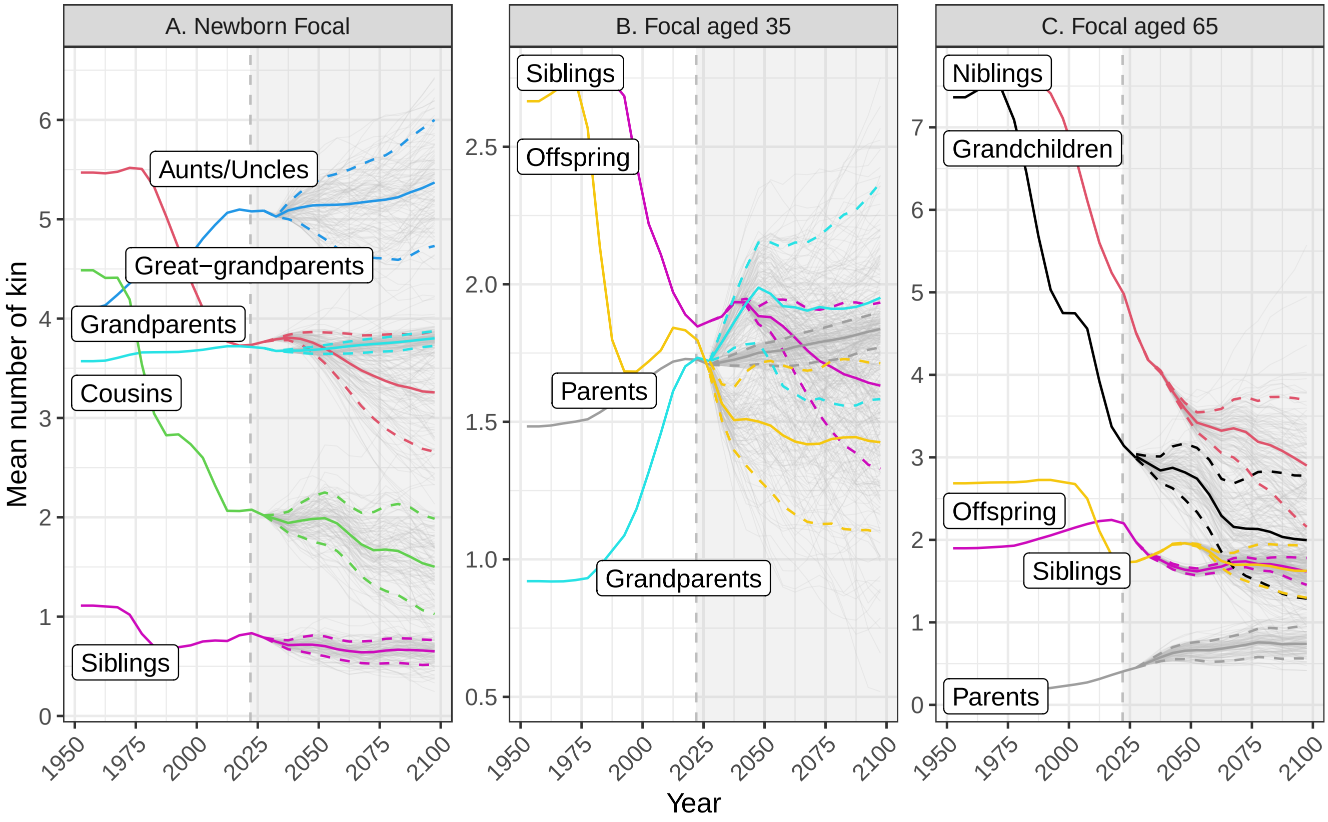

Fig. S19. Expected number of selected kin for a newborn woman in the United States of America (Panel A), a Focal aged 35 (Panel B), and a Focal aged 65 (Panel C). Grey lines in background after 2021 show 100 trajectories estimated using probabilistic trajectories from the 2022 Revision of the United Nations World Population Prospects. Thick lines indicate the median estimates and dashed lines show the 80% projection intervals.

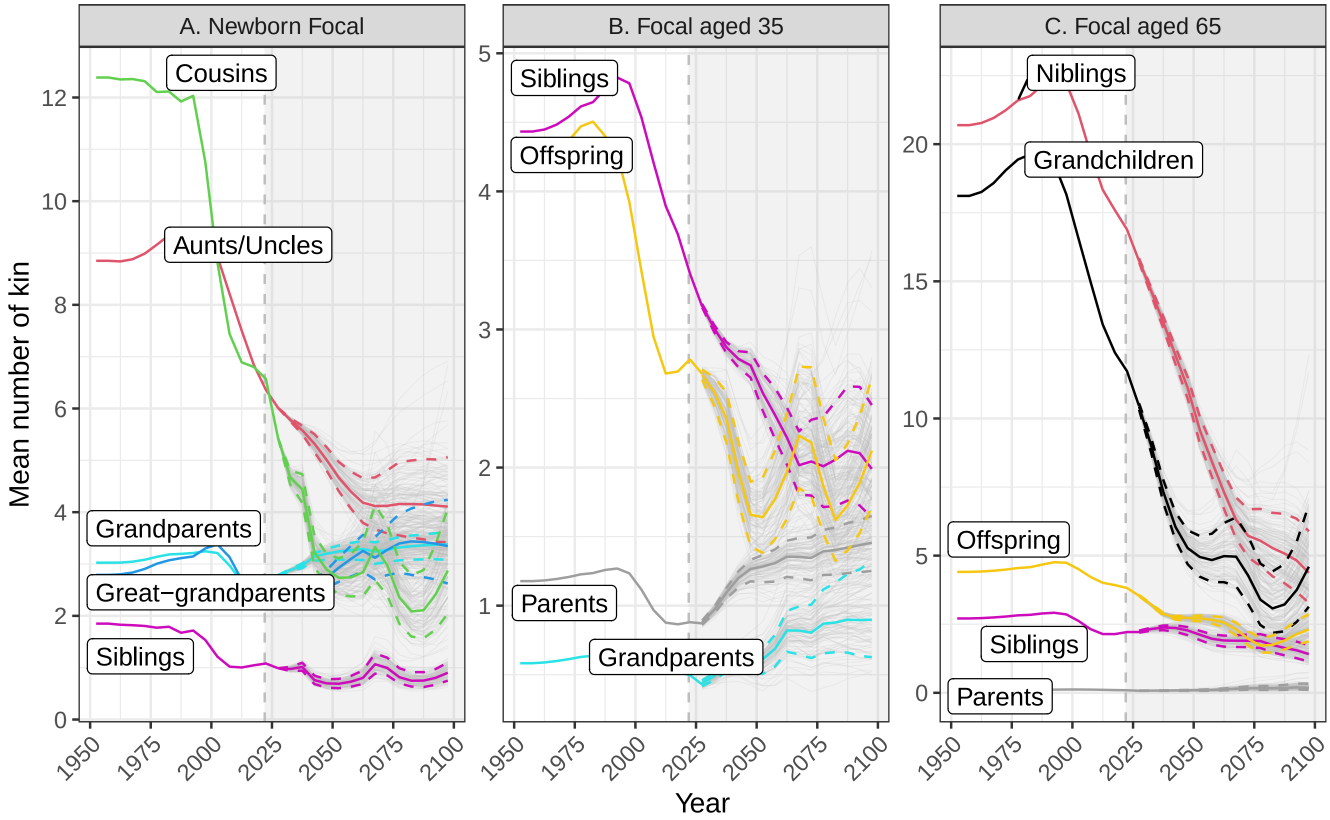

Fig. S20. Expected number of selected kin for a newborn woman in Zimbabwe (Panel A), a Focal aged 35 (Panel B), and a Focal aged 65 (Panel C). Grey lines in background after 2021 show 100 trajectories estimated using probabilistic trajectories from the 2022 Revision of the United Nations World Population Prospects. Thick lines indicate the median estimates and dashed lines show the 80% projection intervals.

Table S2. Living kin for a female Focal aged 0-4. Country-level averages for selected years (values for 2095-2100 refer to the median of the 1,000 country-level projections). Relative codes: ggd = great-grandchildren, gd = grandchildren, d = children, n = niblings, s = siblings, c = cousins, m = parents, a = aunts/uncles, gm = grandparents, ggm = great-grandparents.

ISO	Year	ggd	gd	d	n	s	c	m	a	gm	ggm
ABW	1950-1955	0	0	0	0.03	1.79	11.39	2	8.77	3.18	2.61
	2020-2025	0	0	0	0.00	0.77	2.49	2	3.99	3.69	4.21
	2095-2100	0	0	0	0.00	0.53	1.08	2	2.75	3.82	4.32
AFG	1950-1955	0	0	0	0.03	1.41	6.11	2	5.94	2.34	1.39
	2020-2025	0	0	0	0.04	1.66	10.21	2	9.29	3.23	2.68
	2095-2100	0	0	0	0.00	0.95	3.48	2	4.49	3.65	4.11
AGO	1950-1955	0	0	0	0.04	1.29	5.38	2	5.64	2.81	2.34
	2020-2025	0	0	0	0.05	1.66	9.45	2	7.99	3.01	2.49
	2095-2100	0	0	0	0.01	1.17	4.75	2	5.19	3.52	3.71
AIA	1950-1955	0	0	0	0.04	1.92	13.55	2	9.90	3.28	3.25
	2020-2025	0	0	0	0.01	0.72	2.93	2	4.47	3.75	4.61
	2095-2100	0	0	0	0.00	0.58	1.26	2	2.85	3.85	5.08
ALB	1950-1955	0	0	0	0.02	1.45	7.65	2	7.40	3.21	2.48
	2020-2025	0	0	0	0.00	0.66	2.75	2	4.91	3.80	4.88
	2095-2100	0	0	0	0.00	0.56	1.44	2	3.26	3.92	5.69
AND	1950-1955	0	0	0	0.00	0.80	2.33	2	4.05	3.42	2.77
	2020-2025	0	0	0	0.00	0.61	1.67	2	3.40	3.83	4.32
	2095-2100	0	0	0	0.00	0.55	1.08	2	2.72	3.91	4.80
ARE	1950-1955	0	0	0	0.05	1.46	7.58	2	7.30	3.01	2.98
	2020-2025	0	0	0	0.01	0.99	8.82	2	8.57	3.65	3.67
	2095-2100	0	0	0	0.00	0.62	1.35	2	3.01	3.90	4.38
ARG	1950-1955	0	0	0	0.02	1.03	3.86	2	5.09	3.42	3.16
	2020-2025	0	0	0	0.01	0.86	3.33	2	4.94	3.65	4.25
	2095-2100	0	0	0	0.00	0.70	1.79	2	3.41	3.83	4.94
ARM	1950-1955	0	0	0	0.02	1.13	5.37	2	7.40	3.47	3.74
	2020-2025	0	0	0	0.00	0.65	1.86	2	3.73	3.69	4.29
	2095-2100	0	0	0	0.00	0.64	1.63	2	3.24	3.79	4.59
ASM	1950-1955	0	0	0	0.03	1.79	11.94	2	9.84	3.36	3.52
	2020-2025	0	0	0	0.01	1.14	6.81	2	7.56	3.61	3.89
	2095-2100	0	0	0	0.00	0.74	2.12	2	3.62	3.66	3.62
ATG	1950-1955	0	0	0	0.04	1.37	7.30	2	7.20	3.31	3.36
	2020-2025	0	0	0	0.00	0.67	1.89	2	3.89	3.75	5.14
	2095-2100	0	0	0	0.00	0.64	1.50	2	3.19	3.89	5.67
AUS	1950-1955	0	0	0	0.01	1.19	4.99	2	5.69	3.56	3.57
	2020-2025	0	0	0	0.00	0.78	2.37	2	4.18	3.79	5.04
	2095-2100	0	0	0	0.00	0.66	1.54	2	3.26	3.90	5.43
AUT	1950-1955	0	0	0	0.00	0.69	1.80	2	3.52	3.56	3.71
	2020-2025	0	0	0	0.00	0.63	1.40	2	3.19	3.77	4.83
	2095-2100	0	0	0	0.00	0.62	1.37	2	3.07	3.88	4.89

Table S2. Living kin for a female Focal aged 0-4. Country-level averages for selected years (values for 2095-2100 refer to the median of the 1,000 country-level projections). Relative codes: ggd = great-grandchildren, gd = grandchildren, d = children, n = niblings, s = siblings, c = cousins, m = parents, a = aunts/uncles, gm = grandparents, ggm = great-grandparents. *(continued)*

ISO	Year	ggd	gd	d	n	s	c	m	a	gm	ggm
AZE	1950-1955	0	0	0	0.02	1.18	5.58	2	7.18	3.16	2.84
	2020-2025	0	0	0	0.00	0.71	2.37	2	4.13	3.64	3.80
	2095-2100	0	0	0	0.00	0.67	1.70	2	3.71	3.82	5.51
BDI	1950-1955	0	0	0	0.04	1.64	9.02	2	7.47	2.79	1.97
	2020-2025	0	0	0	0.03	1.90	10.64	2	8.83	2.93	2.07
	2095-2100	0	0	0	0.01	1.02	3.91	2	4.85	3.48	3.31
BEL	1950-1955	0	0	0	0.00	0.82	2.44	2	4.13	3.55	3.55
	2020-2025	0	0	0	0.00	0.76	1.75	2	3.58	3.76	4.73
	2095-2100	0	0	0	0.00	0.65	1.47	2	3.26	3.89	5.53
BEN	1950-1955	0	0	0	0.05	1.32	5.63	2	5.54	2.78	2.12
	2020-2025	0	0	0	0.04	1.53	9.13	2	8.29	3.31	3.22
	2095-2100	0	0	0	0.01	1.17	4.79	2	5.24	3.52	3.72
BES	1950-1955	0	0	0	0.04	1.73	11.81	2	9.04	3.43	3.33
	2020-2025	0	0	0	0.00	0.77	2.65	2	4.26	3.73	4.33
	2095-2100	0	0	0	0.00	0.65	1.55	2	3.23	3.88	5.01
BFA	1950-1955	0	0	0	0.03	1.24	4.84	2	5.30	2.81	2.19
	2020-2025	0	0	0	0.04	1.58	9.10	2	8.37	3.22	3.02
	2095-2100	0	0	0	0.00	1.00	3.57	2	4.51	3.55	3.77
BGD	1950-1955	0	0	0	0.05	1.35	6.34	2	6.49	3.31	3.38
	2020-2025	0	0	0	0.01	0.74	3.94	2	5.42	3.70	4.86
	2095-2100	0	0	0	0.00	0.70	1.62	2	3.40	3.91	6.50
BGR	1950-1955	0	0	0	0.01	0.73	1.89	2	3.78	3.61	4.60
	2020-2025	0	0	0	0.00	0.67	1.46	2	3.02	3.68	4.78
	2095-2100	0	0	0	0.00	0.68	1.82	2	3.31	3.72	4.20
BHR	1950-1955	0	0	0	0.05	1.55	7.87	2	7.03	3.05	2.95
	2020-2025	0	0	0	0.00	1.05	7.17	2	7.41	3.75	4.24
	2095-2100	0	0	0	0.00	0.75	1.94	2	3.38	3.93	5.43
BHS	1950-1955	0	0	0	0.02	1.46	7.84	2	7.30	3.17	3.01
	2020-2025	0	0	0	0.01	0.67	2.56	2	4.45	3.49	4.19
	2095-2100	0	0	0	0.00	0.59	1.27	2	2.89	3.69	4.56
BIH	1950-1955	0	0	0	0.02	1.13	5.04	2	6.75	3.33	3.11
	2020-2025	0	0	0	0.00	0.58	1.43	2	3.20	3.50	4.11
	2095-2100	0	0	0	0.00	0.57	1.25	2	2.97	3.87	4.84
BLR	1950-1955	0	0	0	0.00	0.70	1.94	2	4.25	3.42	3.36
	2020-2025	0	0	0	0.00	0.73	1.71	2	3.11	3.37	3.24
	2095-2100	0	0	0	0.00	0.63	1.48	2	3.25	3.72	4.33
BLZ	1950-1955	0	0	0	0.04	1.77	11.33	2	8.97	3.21	3.15
	2020-2025	0	0	0	0.01	0.90	5.37	2	6.82	3.60	4.86
	2095-2100	0	0	0	0.00	0.75	1.79	2	3.34	3.83	5.56

Table S2. Living kin for a female Focal aged 0-4. Country-level averages for selected years (values for 2095-2100 refer to the median of the 1,000 country-level projections). Relative codes: ggd = great-grandchildren, gd = grandchildren, d = children, n = niblings, s = siblings, c = cousins, m = parents, a = aunts/uncles, gm = grandparents, ggm = great-grandparents. ***(continued)***

ISO	Year	ggd	gd	d	n	s	c	m	a	gm	ggm
BMU	1950-1955	0	0	0	0.01	1.07	4.07	2	5.28	3.08	2.74
	2020-2025	0	0	0	0.00	0.91	2.36	2	3.62	3.63	4.08
	2095-2100	0	0	0	0.00	0.60	1.31	2	2.90	3.83	4.35
BOL	1950-1955	0	0	0	0.03	1.40	6.59	2	6.60	2.93	2.48
	2020-2025	0	0	0	0.02	1.05	5.41	2	6.44	3.44	3.26
	2095-2100	0	0	0	0.00	0.80	2.22	2	3.70	3.73	4.34
BRA	1950-1955	0	0	0	0.03	1.56	8.38	2	7.70	3.12	3.00
	2020-2025	0	0	0	0.01	0.72	2.92	2	4.35	3.55	4.26
	2095-2100	0	0	0	0.00	0.63	1.59	2	3.20	3.84	5.11
BRB	1950-1955	0	0	0	0.02	1.19	5.46	2	6.18	3.44	3.63
	2020-2025	0	0	0	0.00	0.63	1.71	2	3.39	3.70	4.96
	2095-2100	0	0	0	0.00	0.63	1.43	2	3.20	3.86	5.28
BRN	1950-1955	0	0	0	0.05	1.95	13.28	2	9.55	3.26	3.08
	2020-2025	0	0	0	0.00	0.85	5.02	2	6.39	3.63	3.78
	2095-2100	0	0	0	0.00	0.63	1.45	2	3.24	3.80	4.38
BTN	1950-1955	0	0	0	0.03	1.34	5.76	2	5.85	2.40	1.48
	2020-2025	0	0	0	0.01	0.88	5.64	2	6.62	3.47	3.12
	2095-2100	0	0	0	0.00	0.59	1.22	2	2.95	3.85	5.12
BWA	1950-1955	0	0	0	0.07	1.52	8.32	2	7.41	2.86	2.43
	2020-2025	0	0	0	0.01	1.06	6.14	2	6.27	2.79	2.43
	2095-2100	0	0	0	0.00	0.81	2.31	2	3.79	3.50	3.38
CAF	1950-1955	0	0	0	0.05	1.27	5.35	2	5.43	2.67	2.20
	2020-2025	0	0	0	0.05	1.33	7.60	2	7.41	2.89	2.79
	2095-2100	0	0	0	0.01	1.15	4.60	2	4.81	3.32	3.19
CAN	1950-1955	0	0	0	0.01	1.29	6.01	2	6.32	3.56	3.80
	2020-2025	0	0	0	0.00	0.69	1.73	2	3.53	3.79	4.92
	2095-2100	0	0	0	0.00	0.61	1.32	2	3.01	3.88	5.08
CHE	1950-1955	0	0	0	0.00	0.85	2.56	2	4.20	3.55	3.31
	2020-2025	0	0	0	0.00	0.66	1.59	2	3.41	3.76	4.59
	2095-2100	0	0	0	0.00	0.63	1.39	2	3.14	3.93	5.29
CHL	1950-1955	0	0	0	0.03	1.30	6.31	2	6.78	3.20	2.74
	2020-2025	0	0	0	0.01	0.76	2.66	2	4.36	3.72	4.59
	2095-2100	0	0	0	0.00	0.62	1.44	2	3.10	3.88	5.38
CHN	1950-1955	0	0	0	0.04	1.76	10.67	2	8.16	2.79	1.71
	2020-2025	0	0	0	0.01	0.78	2.30	2	3.85	3.73	4.57
	2095-2100	0	0	0	0.00	0.55	1.12	2	2.76	3.89	5.36
CIV	1950-1955	0	0	0	0.06	1.46	6.80	2	6.37	2.82	2.37
	2020-2025	0	0	0	0.04	1.46	10.31	2	8.39	3.04	2.80
	2095-2100	0	0	0	0.01	1.09	4.12	2	4.90	3.44	3.44

Table S2. Living kin for a female Focal aged 0-4. Country-level averages for selected years (values for 2095-2100 refer to the median of the 1,000 country-level projections). Relative codes: ggd = great-grandchildren, gd = grandchildren, d = children, n = niblings, s = siblings, c = cousins, m = parents, a = aunts/uncles, gm = grandparents, ggm = great-grandparents. ***(continued)***

ISO	Year	ggd	gd	d	n	s	c	m	a	gm	ggm
CMR	1950-1955	0	0	0	0.04	1.13	4.12	2	4.87	2.90	2.69
	2020-2025	0	0	0	0.03	1.46	8.37	2	8.01	3.21	3.35
	2095-2100	0	0	0	0.00	1.07	3.94	2	4.82	3.51	3.74
COD	1950-1955	0	0	0	0.06	1.27	5.63	2	5.91	2.93	2.69
	2020-2025	0	0	0	0.05	1.78	10.17	2	8.59	3.15	2.99
	2095-2100	0	0	0	0.01	1.35	6.28	2	5.78	3.45	3.26
COG	1950-1955	0	0	0	0.04	1.45	6.97	2	6.46	2.75	2.24
	2020-2025	0	0	0	0.04	1.46	8.22	2	7.37	3.04	2.84
	2095-2100	0	0	0	0.01	1.05	3.92	2	4.80	3.48	3.48
COK	1950-1955	0	0	0	0.04	1.63	9.26	2	8.02	2.61	2.06
	2020-2025	0	0	0	0.03	0.97	5.07	2	6.26	3.55	4.12
	2095-2100	0	0	0	0.00	0.77	2.07	2	3.58	3.79	5.22
COL	1950-1955	0	0	0	0.04	1.65	9.80	2	8.33	3.09	3.02
	2020-2025	0	0	0	0.01	0.72	3.12	2	4.64	3.58	4.83
	2095-2100	0	0	0	0.00	0.68	1.73	2	3.36	3.88	5.85
COM	1950-1955	0	0	0	0.04	1.37	6.07	2	5.92	2.74	2.12
	2020-2025	0	0	0	0.05	1.46	9.97	2	8.56	3.23	2.63
	2095-2100	0	0	0	0.01	1.02	3.75	2	4.64	3.51	2.94
CPV	1950-1955	0	0	0	0.03	1.70	10.30	2	8.37	2.87	2.11
	2020-2025	0	0	0	0.01	0.88	4.80	2	6.61	3.55	4.06
	2095-2100	0	0	0	0.00	0.73	1.81	2	3.42	3.86	5.63
CRI	1950-1955	0	0	0	0.04	1.77	11.46	2	9.18	3.32	3.40
	2020-2025	0	0	0	0.01	0.76	3.35	2	4.92	3.77	5.41
	2095-2100	0	0	0	0.00	0.61	1.53	2	3.18	3.90	5.76
CUB	1950-1955	0	0	0	0.03	1.08	4.50	2	5.66	3.63	4.77
	2020-2025	0	0	0	0.00	0.57	1.39	2	3.17	3.80	5.68
	2095-2100	0	0	0	0.00	0.61	1.38	2	3.09	3.86	5.50
CYM	1950-1955	0	0	0	0.02	1.17	5.23	2	6.19	3.27	3.41
	2020-2025	0	0	0	0.00	0.88	2.90	2	3.91	3.59	3.79
	2095-2100	0	0	0	0.00	0.56	1.17	2	2.76	3.72	2.89
CYP	1950-1955	0	0	0	0.01	1.06	4.08	2	5.37	3.22	2.54
	2020-2025	0	0	0	0.00	0.71	2.12	2	4.40	3.76	4.47
	2095-2100	0	0	0	0.00	0.58	1.28	2	2.87	3.84	3.78
CZE	1950-1955	0	0	0	0.01	0.94	3.15	2	4.66	3.61	4.10
	2020-2025	0	0	0	0.00	0.66	1.59	2	3.49	3.72	4.78
	2095-2100	0	0	0	0.00	0.68	1.64	2	3.34	3.87	5.07
DEU	1950-1955	0	0	0	0.00	0.64	1.65	2	3.61	3.64	4.21
	2020-2025	0	0	0	0.00	0.61	1.28	2	3.07	3.75	4.77
	2095-2100	0	0	0	0.00	0.63	1.40	2	3.10	3.84	4.72

Table S2. **Living kin for a female Focal aged 0-4. Country-level averages for selected years (values for 2095-2100 refer to the median of the 1,000 country-level projections). Relative codes: ggd = great-grandchildren, gd = grandchildren, d = children, n = niblings, s = siblings, c = cousins, m = parents, a = aunts/uncles, gm = grandparents, ggm = great-grandparents.** ***(continued)***

ISO	Year	ggd	gd	d	n	s	c	m	a	gm	ggm
DJI	1950-1955	0	0	0	0.03	1.57	8.14	2	7.45	2.81	2.00
	2020-2025	0	0	0	0.01	1.14	7.45	2	7.81	3.03	2.27
	2095-2100	0	0	0	0.00	0.77	2.05	2	3.66	3.52	3.06
DMA	1950-1955	0	0	0	0.03	1.33	6.05	2	6.15	3.02	2.71
	2020-2025	0	0	0	0.00	0.69	2.58	2	4.78	3.66	4.32
	2095-2100	0	0	0	0.00	0.67	1.62	2	3.23	3.82	4.88
DNK	1950-1955	0	0	0	0.01	0.91	3.02	2	4.46	3.69	4.39
	2020-2025	0	0	0	0.00	0.68	1.70	2	3.75	3.75	4.77
	2095-2100	0	0	0	0.00	0.66	1.52	2	3.41	3.91	5.37
DOM	1950-1955	0	0	0	0.04	1.75	10.56	2	8.85	3.13	3.01
	2020-2025	0	0	0	0.01	0.82	3.41	2	4.94	3.62	4.84
	2095-2100	0	0	0	0.00	0.74	1.91	2	3.55	3.82	5.48
DZA	1950-1955	0	0	0	0.04	1.71	10.07	2	8.24	2.96	2.53
	2020-2025	0	0	0	0.01	1.05	7.14	2	7.88	3.56	3.12
	2095-2100	0	0	0	0.00	0.86	2.55	2	3.89	3.90	5.44
ECU	1950-1955	0	0	0	0.04	1.73	10.77	2	8.68	3.18	2.95
	2020-2025	0	0	0	0.01	0.90	4.69	2	5.64	3.64	4.75
	2095-2100	0	0	0	0.00	0.72	1.82	2	3.36	3.87	5.79
EGY	1950-1955	0	0	0	0.04	1.42	6.88	2	6.69	3.19	2.91
	2020-2025	0	0	0	0.01	1.11	5.91	2	6.71	3.60	3.84
	2095-2100	0	0	0	0.01	0.86	2.71	2	4.10	3.84	5.27
ERI	1950-1955	0	0	0	0.03	1.30	5.43	2	5.83	2.63	1.85
	2020-2025	0	0	0	0.02	1.29	7.75	2	7.74	3.25	2.83
	2095-2100	0	0	0	0.00	0.90	2.95	2	4.23	3.73	4.82
ESH	1950-1955	0	0	0	0.04	1.48	7.01	2	6.42	2.55	1.66
	2020-2025	0	0	0	0.03	1.23	6.64	2	6.65	3.20	2.12
	2095-2100	0	0	0	0.00	0.77	2.03	2	3.53	3.78	4.15
ESP	1950-1955	0	0	0	0.00	0.81	2.41	2	4.33	3.43	2.97
	2020-2025	0	0	0	0.00	0.62	1.74	2	3.53	3.67	4.03
	2095-2100	0	0	0	0.00	0.59	1.22	2	2.85	3.87	4.76
EST	1950-1955	0	0	0	0.00	0.70	1.95	2	4.05	3.44	3.45
	2020-2025	0	0	0	0.00	0.77	1.84	2	3.38	3.43	3.42
	2095-2100	0	0	0	0.00	0.66	1.53	2	3.26	3.78	4.53
ETH	1950-1955	0	0	0	0.05	1.56	7.97	2	6.91	2.66	1.79
	2020-2025	0	0	0	0.04	1.50	8.72	2	8.25	3.16	2.79
	2095-2100	0	0	0	0.00	0.94	3.35	2	4.46	3.68	4.39
FIN	1950-1955	0	0	0	0.01	1.06	4.25	2	5.34	3.34	2.83
	2020-2025	0	0	0	0.00	0.76	1.93	2	3.64	3.68	4.21
	2095-2100	0	0	0	0.00	0.61	1.32	2	3.01	3.88	5.28

Table S2. Living kin for a female Focal aged 0-4. Country-level averages for selected years (values for 2095-2100 refer to the median of the 1,000 country-level projections). Relative codes: ggd = great-grandchildren, gd = grandchildren, d = children, n = niblings, s = siblings, c = cousins, m = parents, a = aunts/uncles, gm = grandparents, ggm = great-grandparents. ***(continued)***

ISO	Year	ggd	gd	d	n	s	c	m	a	gm	ggm
FJI	1950-1955	0	0	0	0.05	1.78	11.83	2	9.52	3.15	2.40
	2020-2025	0	0	0	0.01	1.05	4.64	2	6.10	3.50	3.56
	2095-2100	0	0	0	0.00	0.81	2.27	2	3.80	3.57	3.40
FLK	1950-1955	0	0	0	0.00	0.87	2.53	2	4.24	3.17	3.07
	2020-2025	0	0	0	0.00	0.68	1.60	2	3.19	3.61	3.77
	2095-2100	0	0	0	0.00	0.64	1.50	2	3.17	3.92	5.37
FRA	1950-1955	0	0	0	0.01	0.95	3.28	2	4.91	3.54	3.77
	2020-2025	0	0	0	0.00	0.81	2.29	2	4.00	3.71	4.75
	2095-2100	0	0	0	0.00	0.67	1.64	2	3.45	3.85	5.52
FRO	1950-1955	0	0	0	0.01	1.30	5.96	2	6.19	3.26	2.57
	2020-2025	0	0	0	0.00	0.97	3.59	2	5.22	3.80	4.65
	2095-2100	0	0	0	0.00	0.85	2.51	2	4.31	3.93	5.68
FSM	1950-1955	0	0	0	0.04	1.79	11.36	2	8.84	3.01	2.47
	2020-2025	0	0	0	0.01	1.06	7.54	2	8.07	3.59	3.65
	2095-2100	0	0	0	0.00	0.76	2.11	2	3.79	3.76	4.25
GAB	1950-1955	0	0	0	0.02	0.97	3.09	2	4.08	2.97	2.72
	2020-2025	0	0	0	0.03	1.39	7.39	2	7.29	3.35	3.48
	2095-2100	0	0	0	0.00	0.90	2.90	2	4.18	3.58	3.95
GBR	1950-1955	0	0	0	0.00	0.84	2.51	2	4.00	3.58	3.47
	2020-2025	0	0	0	0.00	0.78	1.99	2	3.70	3.78	4.92
	2095-2100	0	0	0	0.00	0.65	1.52	2	3.23	3.89	5.39
GEO	1950-1955	0	0	0	0.01	0.75	2.21	2	4.47	3.29	3.23
	2020-2025	0	0	0	0.01	0.75	1.92	2	3.46	3.55	3.94
	2095-2100	0	0	0	0.00	0.75	2.19	2	3.75	3.65	3.81
GGY	1950-1955	0	0	0	0.00	0.99	3.18	2	4.12	3.58	3.35
	2020-2025	0	0	0	0.00	0.65	1.48	2	3.23	3.86	5.06
	2095-2100	0	0	0	0.00	0.61	1.36	2	3.08	3.92	5.33
GHA	1950-1955	0	0	0	0.05	1.59	8.28	2	7.56	2.98	2.65
	2020-2025	0	0	0	0.02	1.36	8.04	2	7.58	3.26	3.04
	2095-2100	0	0	0	0.00	0.92	2.94	2	4.24	3.56	3.74
GIN	1950-1955	0	0	0	0.04	1.28	5.26	2	5.43	2.85	2.35
	2020-2025	0	0	0	0.04	1.36	7.05	2	7.47	3.29	3.29
	2095-2100	0	0	0	0.01	0.96	3.33	2	4.34	3.53	3.79
GLP	1950-1955	0	0	0	0.03	1.68	10.01	2	8.06	2.70	1.65
	2020-2025	0	0	0	0.01	0.76	3.11	2	4.76	3.64	4.32
	2095-2100	0	0	0	0.00	0.68	1.65	2	3.55	3.85	5.35
GMB	1950-1955	0	0	0	0.05	1.20	4.53	2	5.25	2.93	2.79
	2020-2025	0	0	0	0.05	1.71	9.18	2	8.37	3.26	3.34
	2095-2100	0	0	0	0.00	0.95	3.38	2	4.62	3.51	3.29

Table S2. Living kin for a female Focal aged 0-4. Country-level averages for selected years (values for 2095-2100 refer to the median of the 1,000 country-level projections). Relative codes: ggd = great-grandchildren, gd = grandchildren, d = children, n = niblings, s = siblings, c = cousins, m = parents, a = aunts/uncles, gm = grandparents, ggm = great-grandparents. ***(continued)***

ISO	Year	ggd	gd	d	n	s	c	m	a	gm	ggm
GNB	1950-1955	0	0	0	0.03	1.38	5.75	2	5.51	2.74	1.96
	2020-2025	0	0	0	0.04	1.46	7.27	2	7.36	3.04	2.49
	2095-2100	0	0	0	0.01	0.91	2.99	2	4.14	3.47	3.42
GNQ	1950-1955	0	0	0	0.05	1.33	5.91	2	5.80	2.90	2.47
	2020-2025	0	0	0	0.06	1.66	8.35	2	7.41	3.20	2.90
	2095-2100	0	0	0	0.01	0.90	2.94	2	4.01	3.53	3.77
GRC	1950-1955	0	0	0	0.00	0.75	2.15	2	4.21	3.54	3.52
	2020-2025	0	0	0	0.00	0.66	1.70	2	3.52	3.72	4.61
	2095-2100	0	0	0	0.00	0.59	1.27	2	2.88	3.81	4.18
GRD	1950-1955	0	0	0	0.03	1.36	7.59	2	7.96	3.28	3.47
	2020-2025	0	0	0	0.01	0.87	3.75	2	5.67	3.61	4.46
	2095-2100	0	0	0	0.00	0.71	1.80	2	3.43	3.81	4.99
GRL	1950-1955	0	0	0	0.02	1.50	8.40	2	7.73	2.82	2.26
	2020-2025	0	0	0	0.00	0.86	2.68	2	4.17	3.54	3.57
	2095-2100	0	0	0	0.00	0.72	1.84	2	3.47	3.72	4.41
GTM	1950-1955	0	0	0	0.04	1.58	8.38	2	7.64	3.01	2.74
	2020-2025	0	0	0	0.02	1.05	6.74	2	7.44	3.41	3.98
	2095-2100	0	0	0	0.00	0.76	1.91	2	3.48	3.81	5.59
GUF	1950-1955	0	0	0	0.03	1.50	7.47	2	6.41	2.89	2.26
	2020-2025	0	0	0	0.02	1.33	6.39	2	6.58	3.68	4.09
	2095-2100	0	0	0	0.00	0.98	3.57	2	4.97	3.86	5.07
GUM	1950-1955	0	0	0	0.02	1.79	11.72	2	9.31	3.04	1.71
	2020-2025	0	0	0	0.01	1.11	4.73	2	5.68	3.63	4.71
	2095-2100	0	0	0	0.00	0.79	2.27	2	3.98	3.79	5.12
GUY	1950-1955	0	0	0	0.03	1.65	9.97	2	8.50	3.09	2.60
	2020-2025	0	0	0	0.01	0.83	3.45	2	5.07	3.45	3.97
	2095-2100	0	0	0	0.00	0.79	2.12	2	3.65	3.63	4.32
HKG	1950-1955	0	0	0	0.01	1.44	7.34	2	7.25	3.15	2.10
	2020-2025	0	0	0	0.00	0.49	1.61	2	3.53	3.78	4.33
	2095-2100	0	0	0	0.00	0.44	0.72	2	2.16	3.88	4.57
HND	1950-1955	0	0	0	0.04	1.73	10.32	2	8.45	3.01	2.76
	2020-2025	0	0	0	0.01	0.99	6.62	2	7.30	3.65	4.21
	2095-2100	0	0	0	0.00	0.78	1.99	2	3.52	3.85	5.54
HRV	1950-1955	0	0	0	0.01	0.77	2.29	2	4.32	3.47	3.64
	2020-2025	0	0	0	0.00	0.71	1.65	2	3.44	3.66	4.25
	2095-2100	0	0	0	0.00	0.60	1.33	2	3.03	3.81	4.06
HTI	1950-1955	0	0	0	0.04	1.38	6.59	2	6.31	2.78	1.91
	2020-2025	0	0	0	0.02	1.11	5.86	2	6.81	3.22	2.64
	2095-2100	0	0	0	0.00	0.80	2.24	2	3.70	3.57	3.35

Table S2. Living kin for a female Focal aged 0-4. Country-level averages for selected years (values for 2095-2100 refer to the median of the 1,000 country-level projections). Relative codes: ggd = great-grandchildren, gd = grandchildren, d = children, n = niblings, s = siblings, c = cousins, m = parents, a = aunts/uncles, gm = grandparents, ggm = great-grandparents. *(continued)*

ISO	Year	ggd	gd	d	n	s	c	m	a	gm	ggm
HUN	1950-1955	0	0	0	0.01	0.79	2.32	2	4.09	3.58	4.14
	2020-2025	0	0	0	0.00	0.60	1.43	2	3.34	3.52	3.93
	2095-2100	0	0	0	0.00	0.65	1.58	2	3.15	3.77	4.25
IDN	1950-1955	0	0	0	0.02	1.18	4.69	2	5.64	3.03	2.92
	2020-2025	0	0	0	0.01	0.91	4.05	2	5.30	3.56	3.78
	2095-2100	0	0	0	0.00	0.74	1.84	2	3.54	3.76	4.66
IMN	1950-1955	0	0	0	0.00	0.76	2.02	2	3.60	3.41	3.69
	2020-2025	0	0	0	0.00	0.72	2.02	2	3.59	3.71	4.52
	2095-2100	0	0	0	0.00	0.62	1.41	2	3.15	3.90	5.73
IND	1950-1955	0	0	0	0.04	1.41	6.85	2	6.65	3.07	2.83
	2020-2025	0	0	0	0.01	0.96	4.20	2	5.52	3.59	4.25
	2095-2100	0	0	0	0.00	0.70	1.78	2	3.54	3.81	5.17
IRL	1950-1955	0	0	0	0.00	1.26	5.73	2	6.28	3.32	2.20
	2020-2025	0	0	0	0.00	0.88	3.47	2	4.93	3.70	3.40
	2095-2100	0	0	0	0.00	0.67	1.60	2	3.33	3.91	4.99
IRN	1950-1955	0	0	0	0.04	1.61	8.75	2	7.74	3.04	2.54
	2020-2025	0	0	0	0.01	0.77	5.18	2	6.35	3.54	3.36
	2095-2100	0	0	0	0.00	0.65	1.56	2	3.29	3.88	5.21
IRQ	1950-1955	0	0	0	0.04	1.67	9.32	2	7.47	2.89	2.18
	2020-2025	0	0	0	0.02	1.33	9.53	2	8.58	3.34	3.19
	2095-2100	0	0	0	0.00	0.94	3.24	2	4.44	3.80	4.89
ISL	1950-1955	0	0	0	0.01	1.31	6.31	2	6.60	3.60	4.31
	2020-2025	0	0	0	0.00	0.84	2.73	2	4.52	3.84	5.30
	2095-2100	0	0	0	0.00	0.64	1.59	2	3.29	3.93	5.21
ISR	1950-1955	0	0	0	0.03	1.56	8.99	2	7.82	3.62	4.14
	2020-2025	0	0	0	0.00	1.17	5.12	2	6.24	3.80	5.05
	2095-2100	0	0	0	0.00	0.96	3.30	2	4.71	3.90	5.65
ITA	1950-1955	0	0	0	0.00	0.76	2.12	2	3.95	3.53	3.40
	2020-2025	0	0	0	0.00	0.61	1.49	2	3.24	3.75	4.41
	2095-2100	0	0	0	0.00	0.59	1.26	2	2.87	3.89	4.56
JAM	1950-1955	0	0	0	0.02	1.15	5.07	2	5.98	3.42	3.93
	2020-2025	0	0	0	0.01	0.67	2.90	2	4.88	3.70	4.81
	2095-2100	0	0	0	0.00	0.57	1.23	2	2.90	3.75	4.43
JEY	1950-1955	0	0	0	0.00	0.76	2.06	2	3.71	3.64	4.05
	2020-2025	0	0	0	0.00	0.73	1.59	2	3.19	3.83	4.82
	2095-2100	0	0	0	0.00	0.63	1.36	2	3.04	3.93	5.27
JOR	1950-1955	0	0	0	0.04	1.68	10.17	2	8.68	2.90	2.38
	2020-2025	0	0	0	0.01	1.29	10.02	2	9.18	3.69	4.03
	2095-2100	0	0	0	0.00	0.81	2.46	2	4.07	3.90	5.47

Table S2. Living kin for a female Focal aged 0-4. Country-level averages for selected years (values for 2095-2100 refer to the median of the 1,000 country-level projections). Relative codes: ggd = great-grandchildren, gd = grandchildren, d = children, n = niblings, s = siblings, c = cousins, m = parents, a = aunts/uncles, gm = grandparents, ggm = great-grandparents. *(continued)*

ISO	Year	ggd	gd	d	n	s	c	m	a	gm	ggm
JPN	1950-1955	0	0	0	0.00	0.88	2.88	2	5.08	3.46	3.39
	2020-2025	0	0	0	0.00	0.62	1.58	2	3.51	3.79	4.89
	2095-2100	0	0	0	0.00	0.60	1.33	2	2.92	3.86	4.63
KAZ	1950-1955	0	0	0	0.01	1.14	5.06	2	6.61	3.22	3.18
	2020-2025	0	0	0	0.00	0.98	3.31	2	4.47	3.28	2.99
	2095-2100	0	0	0	0.00	0.98	3.51	2	4.66	3.62	3.89
KEN	1950-1955	0	0	0	0.07	1.65	8.55	2	7.34	2.86	2.61
	2020-2025	0	0	0	0.04	1.31	8.85	2	8.28	3.12	3.33
	2095-2100	0	0	0	0.00	0.89	2.77	2	4.10	3.54	3.93
KGZ	1950-1955	0	0	0	0.01	1.13	5.01	2	6.65	3.17	3.00
	2020-2025	0	0	0	0.01	1.02	4.58	2	5.83	3.44	3.43
	2095-2100	0	0	0	0.00	0.86	2.70	2	4.12	3.78	5.22
KHM	1950-1955	0	0	0	0.03	1.58	8.08	2	7.23	2.73	2.02
	2020-2025	0	0	0	0.01	0.93	4.29	2	6.30	3.41	2.70
	2095-2100	0	0	0	0.00	0.75	1.87	2	3.57	3.81	5.78
KIR	1950-1955	0	0	0	0.03	1.65	9.32	2	7.84	2.92	2.29
	2020-2025	0	0	0	0.01	1.20	6.61	2	7.27	3.45	3.17
	2095-2100	0	0	0	0.00	0.92	2.97	2	4.44	3.68	3.77
KNA	1950-1955	0	0	0	0.07	2.11	15.44	2	9.38	3.15	2.84
	2020-2025	0	0	0	0.01	0.68	2.65	2	4.52	3.59	4.12
	2095-2100	0	0	0	0.00	0.64	1.47	2	3.09	3.71	4.52
KOR	1950-1955	0	0	0	0.02	1.46	6.22	2	5.71	2.00	0.95
	2020-2025	0	0	0	0.00	0.61	1.73	2	3.99	3.69	3.78
	2095-2100	0	0	0	0.00	0.45	0.72	2	2.44	3.89	4.52
KWT	1950-1955	0	0	0	0.02	2.05	12.67	2	9.06	3.27	3.20
	2020-2025	0	0	0	0.01	1.20	9.28	2	7.87	3.74	4.05
	2095-2100	0	0	0	0.00	0.68	1.82	2	3.97	3.96	6.67
LAO	1950-1955	0	0	0	0.03	1.47	7.25	2	6.70	2.78	2.03
	2020-2025	0	0	0	0.01	0.96	5.53	2	6.83	3.45	3.32
	2095-2100	0	0	0	0.00	0.77	2.01	2	3.71	3.83	5.52
LBN	1950-1955	0	0	0	0.05	1.70	10.96	2	9.04	3.44	3.62
	2020-2025	0	0	0	0.01	0.81	3.79	2	5.52	3.70	3.92
	2095-2100	0	0	0	0.00	0.71	1.82	2	3.51	3.92	5.98
LBR	1950-1955	0	0	0	0.04	1.35	5.98	2	6.06	2.91	2.52
	2020-2025	0	0	0	0.05	1.39	7.75	2	7.35	3.23	3.08
	2095-2100	0	0	0	0.01	0.96	3.24	2	4.27	3.57	3.97
LBY	1950-1955	0	0	0	0.03	1.46	6.58	2	6.34	2.69	1.91
	2020-2025	0	0	0	0.01	1.00	8.53	2	9.24	3.45	2.74
	2095-2100	0	0	0	0.00	0.72	1.84	2	3.56	3.78	4.61

Table S2. Living kin for a female Focal aged 0-4. Country-level averages for selected years (values for 2095-2100 refer to the median of the 1,000 country-level projections). Relative codes: ggd = great-grandchildren, gd = grandchildren, d = children, n = niblings, s = siblings, c = cousins, m = parents, a = aunts/uncles, gm = grandparents, ggm = great-grandparents. ***(continued)***

ISO	Year	ggd	gd	d	n	s	c	m	a	gm	ggm
LCA	1950-1955	0	0	0	0.03	1.49	7.91	2	7.47	3.22	3.20
	2020-2025	0	0	0	0.01	0.60	3.10	2	5.34	3.67	4.43
	2095-2100	0	0	0	0.00	0.61	1.36	2	2.98	3.80	4.71
LIE	1950-1955	0	0	0	0.00	0.91	2.71	2	4.13	3.19	2.54
	2020-2025	0	0	0	0.00	0.64	1.63	2	3.39	3.79	4.20
	2095-2100	0	0	0	0.00	0.61	1.29	2	3.06	3.94	5.29
LKA	1950-1955	0	0	0	0.02	1.62	9.04	2	8.11	3.31	3.22
	2020-2025	0	0	0	0.00	0.84	3.47	2	4.92	3.48	3.53
	2095-2100	0	0	0	0.00	0.68	1.64	2	3.47	3.87	5.45
LSO	1950-1955	0	0	0	0.05	1.39	7.07	2	6.61	2.77	2.09
	2020-2025	0	0	0	0.02	0.97	5.16	2	5.78	2.33	1.93
	2095-2100	0	0	0	0.00	0.80	2.18	2	3.53	3.20	2.83
LTU	1950-1955	0	0	0	0.01	0.84	2.77	2	4.85	3.41	3.45
	2020-2025	0	0	0	0.00	0.68	1.62	2	3.26	3.37	3.33
	2095-2100	0	0	0	0.00	0.66	1.54	2	3.28	3.66	3.78
LUX	1950-1955	0	0	0	0.00	0.73	2.01	2	3.86	3.51	3.58
	2020-2025	0	0	0	0.00	0.77	1.73	2	3.44	3.72	4.26
	2095-2100	0	0	0	0.00	0.61	1.33	2	3.01	3.86	4.63
LVA	1950-1955	0	0	0	0.00	0.62	1.54	2	3.53	3.43	3.40
	2020-2025	0	0	0	0.00	0.68	1.55	2	3.14	3.34	3.18
	2095-2100	0	0	0	0.00	0.64	1.47	2	3.14	3.70	4.15
MAC	1950-1955	0	0	0	0.01	1.85	12.13	2	9.10	3.23	2.29
	2020-2025	0	0	0	0.00	0.43	0.99	2	2.98	3.82	4.56
	2095-2100	0	0	0	0.00	0.57	1.19	2	2.86	3.93	5.80
MAF	1950-1955	0	0	0	0.03	1.99	14.35	2	9.89	3.41	3.15
	2020-2025	0	0	0	0.01	1.05	6.15	2	6.35	3.74	4.00
	2095-2100	0	0	0	0.00	0.76	2.15	2	3.90	3.89	5.03
MAR	1950-1955	0	0	0	0.06	1.42	7.05	2	7.00	2.88	2.54
	2020-2025	0	0	0	0.01	0.95	6.06	2	6.67	3.47	3.21
	2095-2100	0	0	0	0.00	0.74	1.96	2	3.56	3.88	5.15
MCO	1950-1955	0	0	0	0.01	1.15	4.57	2	5.32	3.49	3.24
	2020-2025	0	0	0	0.00	0.84	2.53	2	3.95	3.84	4.69
	2095-2100	0	0	0	0.00	0.75	1.97	2	3.86	3.96	6.26
MDA	1950-1955	0	0	0	0.01	1.03	3.90	2	5.24	3.13	2.55
	2020-2025	0	0	0	0.00	0.70	1.92	2	3.56	3.41	3.30
	2095-2100	0	0	0	0.00	0.68	1.69	2	3.41	3.63	3.47
MDG	1950-1955	0	0	0	0.06	1.84	10.60	2	8.07	2.80	2.28
	2020-2025	0	0	0	0.04	1.32	8.28	2	7.84	3.39	3.63
	2095-2100	0	0	0	0.01	0.98	3.36	2	4.40	3.70	4.62

Table S2. Living kin for a female Focal aged 0-4. Country-level averages for selected years (values for 2095-2100 refer to the median of the 1,000 country-level projections). Relative codes: ggd = great-grandchildren, gd = grandchildren, d = children, n = niblings, s = siblings, c = cousins, m = parents, a = aunts/uncles, gm = grandparents, ggm = great-grandparents. ***(continued)***

ISO	Year	ggd	gd	d	n	s	c	m	a	gm	ggm
MDV	1950-1955	0	0	0	0.02	1.25	5.08	2	6.05	3.25	3.48
	2020-2025	0	0	0	0.01	1.03	6.71	2	7.75	3.76	4.40
	2095-2100	0	0	0	0.00	0.66	1.59	2	3.25	3.94	5.66
MEX	1950-1955	0	0	0	0.04	1.66	9.63	2	8.22	3.00	2.78
	2020-2025	0	0	0	0.01	0.79	4.03	2	5.45	3.64	4.66
	2095-2100	0	0	0	0.00	0.69	1.59	2	3.27	3.84	5.78
MHL	1950-1955	0	0	0	0.05	2.22	17.77	2	10.76	2.97	2.55
	2020-2025	0	0	0	0.02	1.33	10.17	2	8.98	3.41	3.56
	2095-2100	0	0	0	0.00	0.75	1.98	2	3.57	3.52	3.44
MKD	1950-1955	0	0	0	0.02	1.32	6.34	2	7.14	3.46	3.35
	2020-2025	0	0	0	0.00	0.67	1.89	2	3.88	3.75	4.50
	2095-2100	0	0	0	0.00	0.60	1.37	2	3.01	3.87	4.79
MLI	1950-1955	0	0	0	0.05	1.34	5.40	2	5.26	2.65	1.87
	2020-2025	0	0	0	0.06	1.72	9.37	2	8.47	3.30	3.11
	2095-2100	0	0	0	0.01	1.24	5.27	2	5.26	3.54	3.70
MLT	1950-1955	0	0	0	0.01	1.41	7.15	2	7.16	3.44	2.78
	2020-2025	0	0	0	0.00	0.65	1.84	2	3.77	3.79	4.42
	2095-2100	0	0	0	0.00	0.61	1.39	2	2.98	3.88	4.42
MMR	1950-1955	0	0	0	0.02	1.38	6.23	2	6.18	2.65	1.68
	2020-2025	0	0	0	0.01	0.81	3.96	2	5.30	3.23	2.61
	2095-2100	0	0	0	0.00	0.75	1.84	2	3.41	3.70	4.17
MNE	1950-1955	0	0	0	0.02	1.18	5.80	2	7.27	3.41	3.33
	2020-2025	0	0	0	0.00	0.78	2.15	2	3.88	3.68	4.19
	2095-2100	0	0	0	0.00	0.62	1.44	2	3.18	3.83	4.56
MNG	1950-1955	0	0	0	0.02	1.44	7.05	2	6.59	2.65	1.25
	2020-2025	0	0	0	0.01	1.00	5.37	2	5.89	3.27	2.74
	2095-2100	0	0	0	0.00	0.76	2.23	2	3.80	3.80	5.20
MNP	1950-1955	0	0	0	0.04	2.07	14.53	2	9.62	3.12	2.53
	2020-2025	0	0	0	0.01	0.96	5.52	2	5.52	3.76	4.55
	2095-2100	0	0	0	0.00	0.73	1.85	2	3.64	3.89	5.65
MOZ	1950-1955	0	0	0	0.05	1.41	6.69	2	5.96	2.80	2.10
	2020-2025	0	0	0	0.05	1.39	7.09	2	7.04	2.99	2.58
	2095-2100	0	0	0	0.01	1.03	3.71	2	4.39	3.58	3.86
MRT	1950-1955	0	0	0	0.05	1.43	6.80	2	6.45	2.78	2.18
	2020-2025	0	0	0	0.04	1.45	9.50	2	8.57	3.41	3.15
	2095-2100	0	0	0	0.01	1.06	4.12	2	4.92	3.67	4.00
MSR	1950-1955	0	0	0	0.03	1.34	6.46	2	6.42	3.09	3.05
	2020-2025	0	0	0	0.00	0.48	1.71	2	3.56	3.75	5.10
	2095-2100	0	0	0	0.00	0.66	1.66	2	3.34	3.86	5.35

Table S2. Living kin for a female Focal aged 0-4. Country-level averages for selected years (values for 2095-2100 refer to the median of the 1,000 country-level projections). Relative codes: ggd = great-grandchildren, gd = grandchildren, d = children, n = niblings, s = siblings, c = cousins, m = parents, a = aunts/uncles, gm = grandparents, ggm = great-grandparents. ***(continued)***

ISO	Year	ggd	gd	d	n	s	c	m	a	gm	ggm
MTQ	1950-1955	0	0	0	0.03	1.81	11.90	2	8.94	2.84	1.74
	2020-2025	0	0	0	0.00	0.69	2.42	2	4.29	3.72	4.59
	2095-2100	0	0	0	0.00	0.65	1.61	2	3.53	3.87	5.39
MUS	1950-1955	0	0	0	0.03	1.70	10.34	2	8.58	2.96	1.76
	2020-2025	0	0	0	0.00	0.70	2.31	2	4.05	3.60	4.04
	2095-2100	0	0	0	0.00	0.56	1.18	2	2.91	3.77	4.34
MWI	1950-1955	0	0	0	0.05	1.31	5.45	2	5.78	2.88	2.48
	2020-2025	0	0	0	0.04	1.38	7.92	2	7.41	2.86	2.62
	2095-2100	0	0	0	0.00	0.93	3.00	2	4.15	3.60	4.33
MYS	1950-1955	0	0	0	0.04	1.81	11.50	2	8.84	2.90	1.94
	2020-2025	0	0	0	0.00	0.87	4.72	2	6.60	3.56	3.68
	2095-2100	0	0	0	0.00	0.64	1.51	2	3.26	3.81	4.56
MYT	1950-1955	0	0	0	0.09	1.92	12.07	2	8.41	2.96	2.43
	2020-2025	0	0	0	0.04	1.61	12.87	2	9.98	3.67	4.17
	2095-2100	0	0	0	0.00	1.13	4.71	2	5.65	3.87	5.72
NAM	1950-1955	0	0	0	0.04	1.42	6.82	2	6.43	2.85	2.24
	2020-2025	0	0	0	0.02	1.12	6.69	2	6.70	2.64	2.31
	2095-2100	0	0	0	0.00	0.88	2.85	2	4.12	3.39	3.05
NCL	1950-1955	0	0	0	0.03	1.53	8.05	2	6.99	2.96	2.34
	2020-2025	0	0	0	0.01	0.87	4.07	2	5.63	3.64	4.34
	2095-2100	0	0	0	0.00	0.75	1.97	2	3.50	3.84	5.26
NER	1950-1955	0	0	0	0.07	1.56	8.15	2	7.19	2.85	2.42
	2020-2025	0	0	0	0.08	1.87	10.62	2	9.16	3.36	3.37
	2095-2100	0	0	0	0.02	1.55	8.15	2	6.44	3.67	4.34
NGA	1950-1955	0	0	0	0.04	1.38	6.19	2	6.12	2.75	2.05
	2020-2025	0	0	0	0.05	1.58	8.63	2	7.70	3.03	2.71
	2095-2100	0	0	0	0.00	1.06	3.98	2	4.62	3.41	3.35
NIC	1950-1955	0	0	0	0.04	1.61	9.06	2	8.04	2.94	2.62
	2020-2025	0	0	0	0.01	0.84	4.64	2	5.94	3.57	4.31
	2095-2100	0	0	0	0.00	0.78	2.02	2	3.59	3.85	5.82
NIU	1950-1955	0	0	0	0.03	1.65	10.57	2	9.43	3.36	3.65
	2020-2025	0	0	0	0.01	1.11	6.97	2	7.54	3.39	3.14
	2095-2100	0	0	0	0.00	0.75	1.94	2	3.63	3.72	3.95
NLD	1950-1955	0	0	0	0.00	1.12	4.57	2	5.72	3.59	3.23
	2020-2025	0	0	0	0.00	0.68	1.68	2	3.66	3.80	4.33
	2095-2100	0	0	0	0.00	0.63	1.40	2	3.26	3.92	5.43
NOR	1950-1955	0	0	0	0.01	1.01	3.62	2	4.65	3.61	3.73
	2020-2025	0	0	0	0.00	0.79	2.10	2	3.98	3.80	4.81
	2095-2100	0	0	0	0.00	0.62	1.37	2	3.19	3.93	5.59

Table S2. **Living kin for a female Focal aged 0-4. Country-level averages for selected years (values for 2095-2100 refer to the median of the 1,000 country-level projections). Relative codes: ggd = great-grandchildren, gd = grandchildren, d = children, n = niblings, s = siblings, c = cousins, m = parents, a = aunts/uncles, gm = grandparents, ggm = great-grandparents.** ***(continued)***

ISO	Year	ggd	gd	d	n	s	c	m	a	gm	ggm
	1950-1955	0	0	0	0.03	1.31	5.90	2	6.20	2.94	2.51
NPL	2020-2025	0	0	0	0.01	0.67	3.74	2	5.87	3.61	4.11
	2095-2100	0	0	0	0.00	0.71	1.57	2	3.39	3.90	6.37
	1950-1955	0	0	0	0.01	1.55	8.27	2	7.77	2.99	1.91
NRU	2020-2025	0	0	0	0.01	1.24	5.48	2	5.77	3.00	2.12
	2095-2100	0	0	0	0.00	0.90	2.96	2	4.41	3.53	3.85
	1950-1955	0	0	0	0.01	1.33	6.34	2	6.53	3.60	3.87
NZL	2020-2025	0	0	0	0.00	0.81	2.55	2	4.39	3.79	5.18
	2095-2100	0	0	0	0.00	0.65	1.53	2	3.22	3.89	5.56
	1950-1955	0	0	0	0.07	1.25	5.00	2	5.25	2.88	2.77
OMN	2020-2025	0	0	0	0.02	1.40	10.58	2	9.73	3.61	3.56
	2095-2100	0	0	0	0.00	0.81	2.29	2	3.74	3.91	4.89
	1950-1955	0	0	0	0.04	1.47	7.32	2	6.94	3.06	2.72
PAK	2020-2025	0	0	0	0.02	1.27	8.97	2	8.54	3.54	3.73
	2095-2100	0	0	0	0.00	0.89	2.82	2	4.26	3.73	4.44
	1950-1955	0	0	0	0.03	1.56	8.95	2	8.14	3.37	3.77
PAN	2020-2025	0	0	0	0.01	0.90	3.84	2	5.18	3.71	5.39
	2095-2100	0	0	0	0.00	0.78	2.06	2	3.63	3.87	6.09
	1950-1955	0	0	0	0.04	1.66	9.68	2	8.22	3.30	3.32
PER	2020-2025	0	0	0	0.01	0.91	4.93	2	5.86	3.60	4.29
	2095-2100	0	0	0	0.00	0.74	1.85	2	3.44	3.88	5.73
	1950-1955	0	0	0	0.04	2.10	16.03	2	10.73	3.08	2.45
PHL	2020-2025	0	0	0	0.01	1.09	6.39	2	7.06	3.62	3.73
	2095-2100	0	0	0	0.00	0.82	2.39	2	3.84	3.78	4.81
	1950-1955	0	0	0	0.04	2.06	14.79	2	9.46	2.80	2.04
PLW	2020-2025	0	0	0	0.01	0.77	3.53	2	4.78	3.22	2.60
	2095-2100	0	0	0	0.00	0.77	1.97	2	3.67	3.53	3.42
	1950-1955	0	0	0	0.02	1.40	6.30	2	6.33	2.64	1.94
PNG	2020-2025	0	0	0	0.03	1.23	7.72	2	7.54	3.29	2.78
	2095-2100	0	0	0	0.00	0.85	2.62	2	4.04	3.58	3.46
	1950-1955	0	0	0	0.01	1.09	4.42	2	5.85	3.41	3.34
POL	2020-2025	0	0	0	0.00	0.62	1.73	2	3.58	3.58	3.98
	2095-2100	0	0	0	0.00	0.62	1.40	2	3.10	3.79	4.59
	1950-1955	0	0	0	0.03	1.49	8.44	2	7.85	3.40	3.86
PRI	2020-2025	0	0	0	0.00	0.60	1.88	2	3.84	3.68	5.23
	2095-2100	0	0	0	0.00	0.62	1.37	2	3.06	3.81	5.68
	1950-1955	0	0	0	0.00	0.49	0.75	2	2.20	1.78	1.15
PRK	2020-2025	0	0	0	0.00	0.62	1.84	2	4.18	3.62	3.66
	2095-2100	0	0	0	0.00	0.68	1.53	2	3.38	3.78	5.01

Table S2. Living kin for a female Focal aged 0-4. Country-level averages for selected years (values for 2095-2100 refer to the median of the 1,000 country-level projections). Relative codes: ggd = great-grandchildren, gd = grandchildren, d = children, n = niblings, s = siblings, c = cousins, m = parents, a = aunts/uncles, gm = grandparents, ggm = great-grandparents. ***(continued)***

ISO	Year	ggd	gd	d	n	s	c	m	a	gm	ggm
PRT	1950-1955	0	0	0	0.01	0.97	3.47	2	4.94	3.36	3.02
	2020-2025	0	0	0	0.00	0.64	1.75	2	3.51	3.63	4.22
	2095-2100	0	0	0	0.00	0.58	1.26	2	2.93	3.81	4.18
PRY	1950-1955	0	0	0	0.05	1.84	12.81	2	9.77	3.25	2.85
	2020-2025	0	0	0	0.01	0.94	5.49	2	6.56	3.57	4.12
	2095-2100	0	0	0	0.00	0.79	2.14	2	3.65	3.80	5.05
PSE	1950-1955	0	0	0	0.04	1.85	12.33	2	9.68	2.92	2.48
	2020-2025	0	0	0	0.01	1.37	10.40	2	9.89	3.68	4.13
	2095-2100	0	0	0	0.00	0.90	2.96	2	4.31	3.92	6.20
PYF	1950-1955	0	0	0	0.03	1.55	8.90	2	8.16	3.26	3.28
	2020-2025	0	0	0	0.01	0.83	4.24	2	5.88	3.85	5.19
	2095-2100	0	0	0	0.00	0.64	1.55	2	3.23	3.93	5.89
QAT	1950-1955	0	0	0	0.07	1.69	10.46	2	8.62	3.39	3.82
	2020-2025	0	0	0	0.01	1.06	8.80	2	8.48	3.77	4.45
	2095-2100	0	0	0	0.00	0.75	1.91	2	3.36	3.94	5.43
ROU	1950-1955	0	0	0	0.01	0.90	3.14	2	5.02	3.58	3.99
	2020-2025	0	0	0	0.00	0.63	1.64	2	3.24	3.59	4.32
	2095-2100	0	0	0	0.00	0.66	1.57	2	3.35	3.82	4.85
RUS	1950-1955	0	0	0	0.01	0.80	2.62	2	4.70	3.08	2.54
	2020-2025	0	0	0	0.00	0.74	1.70	2	2.97	3.20	2.92
	2095-2100	0	0	0	0.00	0.66	1.59	2	3.34	3.66	4.13
RWA	1950-1955	0	0	0	0.08	1.76	11.33	2	9.09	3.03	2.67
	2020-2025	0	0	0	0.02	1.48	10.41	2	8.66	2.88	2.30
	2095-2100	0	0	0	0.00	0.94	3.18	2	4.44	3.59	3.55
SAU	1950-1955	0	0	0	0.11	1.67	9.78	2	7.64	3.05	2.94
	2020-2025	0	0	0	0.04	1.46	11.31	2	9.58	3.60	3.56
	2095-2100	0	0	0	0.00	0.74	2.02	2	3.69	3.90	5.35
SDN	1950-1955	0	0	0	0.06	1.63	9.42	2	8.19	3.20	3.31
	2020-2025	0	0	0	0.03	1.47	9.57	2	8.04	3.11	2.97
	2095-2100	0	0	0	0.00	1.05	3.96	2	4.90	3.57	3.66
SEN	1950-1955	0	0	0	0.06	1.46	7.16	2	6.64	2.99	2.66
	2020-2025	0	0	0	0.03	1.52	9.85	2	8.64	3.35	3.17
	2095-2100	0	0	0	0.01	1.11	4.59	2	5.32	3.80	4.91
SGP	1950-1955	0	0	0	0.04	2.08	15.05	2	9.79	3.02	1.79
	2020-2025	0	0	0	0.00	0.57	1.67	2	3.64	3.78	4.29
	2095-2100	0	0	0	0.00	0.49	0.87	2	2.51	3.87	4.54
SHN	1950-1955	0	0	0	0.02	1.84	12.42	2	9.65	3.58	4.08
	2020-2025	0	0	0	0.00	0.68	2.85	2	4.53	3.73	4.47
	2095-2100	0	0	0	0.00	0.62	1.41	2	3.16	3.87	4.82

Table S2. Living kin for a female Focal aged 0-4. Country-level averages for selected years (values for 2095-2100 refer to the median of the 1,000 country-level projections). Relative codes: ggd = great-grandchildren, gd = grandchildren, d = children, n = niblings, s = siblings, c = cousins, m = parents, a = aunts/uncles, gm = grandparents, ggm = great-grandparents. *(continued)*

ISO	Year	ggd	gd	d	n	s	c	m	a	gm	ggm
SLB	1950-1955	0	0	0	0.03	1.76	9.97	2	8.10	2.56	1.57
	2020-2025	0	0	0	0.03	1.40	9.60	2	8.61	3.49	3.35
	2095-2100	0	0	0	0.00	1.05	3.95	2	4.91	3.75	4.58
SLE	1950-1955	0	0	0	0.05	1.08	3.68	2	4.44	2.88	2.85
	2020-2025	0	0	0	0.05	1.38	7.39	2	7.26	3.13	2.99
	2095-2100	0	0	0	0.00	0.86	2.69	2	3.99	3.58	3.85
SLV	1950-1955	0	0	0	0.03	1.49	7.85	2	7.46	2.92	2.73
	2020-2025	0	0	0	0.01	0.69	3.41	2	5.25	3.38	4.15
	2095-2100	0	0	0	0.00	0.69	1.57	2	3.21	3.74	5.24
SMR	1950-1955	0	0	0	0.01	1.08	4.34	2	5.49	3.48	3.48
	2020-2025	0	0	0	0.00	0.59	1.56	2	3.34	3.82	4.64
	2095-2100	0	0	0	0.00	0.54	1.06	2	2.71	3.92	4.92
SOM	1950-1955	0	0	0	0.06	1.83	11.01	2	8.08	2.72	1.80
	2020-2025	0	0	0	0.05	1.73	10.32	2	8.51	3.06	2.56
	2095-2100	0	0	0	0.00	1.27	5.47	2	5.35	3.53	3.79
SPM	1950-1955	0	0	0	0.01	1.37	6.64	2	6.51	2.73	1.79
	2020-2025	0	0	0	0.00	0.72	2.37	2	4.08	3.66	3.85
	2095-2100	0	0	0	0.00	0.59	1.32	2	3.25	3.86	5.39
SRB	1950-1955	0	0	0	0.01	0.80	2.41	2	4.49	3.58	4.20
	2020-2025	0	0	0	0.00	0.65	1.45	2	3.16	3.63	4.18
	2095-2100	0	0	0	0.00	0.63	1.47	2	3.13	3.81	4.25
SSD	1950-1955	0	0	0	0.06	1.29	5.28	2	5.43	2.56	1.82
	2020-2025	0	0	0	0.07	1.54	6.59	2	6.59	2.53	1.72
	2095-2100	0	0	0	0.01	0.92	3.00	2	4.10	3.46	3.45
STP	1950-1955	0	0	0	0.02	1.76	9.00	2	6.88	2.98	2.46
	2020-2025	0	0	0	0.03	1.52	9.00	2	8.36	3.40	3.42
	2095-2100	0	0	0	0.00	0.96	3.38	2	4.51	3.57	3.91
SUR	1950-1955	0	0	0	0.04	1.75	11.61	2	9.65	3.07	2.31
	2020-2025	0	0	0	0.01	0.95	4.01	2	5.43	3.47	3.88
	2095-2100	0	0	0	0.00	0.75	1.99	2	3.61	3.72	4.47
SVK	1950-1955	0	0	0	0.01	1.13	4.67	2	5.84	3.57	4.07
	2020-2025	0	0	0	0.00	0.60	1.63	2	3.61	3.66	4.29
	2095-2100	0	0	0	0.00	0.66	1.72	2	3.32	3.83	4.87
SVN	1950-1955	0	0	0	0.01	0.81	2.54	2	4.48	3.31	2.91
	2020-2025	0	0	0	0.00	0.66	1.54	2	3.31	3.71	4.41
	2095-2100	0	0	0	0.00	0.68	1.67	2	3.32	3.89	5.08
SWE	1950-1955	0	0	0	0.01	0.87	2.69	2	3.99	3.65	4.01
	2020-2025	0	0	0	0.00	0.74	1.96	2	3.85	3.81	4.88
	2095-2100	0	0	0	0.00	0.67	1.59	2	3.34	3.93	5.73

Table S2. Living kin for a female Focal aged 0-4. Country-level averages for selected years (values for 2095-2100 refer to the median of the 1,000 country-level projections). Relative codes: ggd = great-grandchildren, gd = grandchildren, d = children, n = niblings, s = siblings, c = cousins, m = parents, a = aunts/uncles, gm = grandparents, ggm = great-grandparents. ***(continued)***

ISO	Year	ggd	gd	d	n	s	c	m	a	gm	ggm
SWZ	1950-1955	0	0	0	0.08	1.60	9.08	2	7.46	2.86	2.44
	2020-2025	0	0	0	0.02	1.00	5.99	2	6.33	2.36	2.24
	2095-2100	0	0	0	0.00	0.80	2.23	2	3.70	3.42	3.43
SXM	1950-1955	0	0	0	0.01	1.53	9.11	2	8.24	3.41	3.08
	2020-2025	0	0	0	0.00	0.34	1.44	2	3.73	3.84	5.18
	2095-2100	0	0	0	0.00	0.60	1.34	2	3.15	3.86	5.08
SYC	1950-1955	0	0	0	0.04	1.52	8.29	2	7.26	2.72	1.63
	2020-2025	0	0	0	0.01	0.88	3.47	2	4.74	3.52	3.55
	2095-2100	0	0	0	0.00	0.75	2.02	2	3.68	3.86	5.61
SYR	1950-1955	0	0	0	0.05	1.84	11.74	2	9.06	3.02	2.44
	2020-2025	0	0	0	0.02	1.12	8.66	2	8.42	3.56	3.75
	2095-2100	0	0	0	0.00	0.80	2.19	2	3.78	3.79	4.74
TCA	1950-1955	0	0	0	0.03	1.48	8.21	2	7.41	3.11	2.82
	2020-2025	0	0	0	0.01	0.89	4.64	2	6.41	3.64	3.71
	2095-2100	0	0	0	0.00	0.64	1.47	2	3.14	3.84	4.14
TCD	1950-1955	0	0	0	0.04	1.33	5.72	2	5.80	2.87	2.47
	2020-2025	0	0	0	0.08	1.80	9.46	2	8.57	3.07	2.94
	2095-2100	0	0	0	0.01	1.35	6.28	2	5.62	3.28	2.99
TGO	1950-1955	0	0	0	0.05	1.53	7.97	2	7.14	2.99	2.58
	2020-2025	0	0	0	0.03	1.40	8.88	2	7.98	3.24	3.02
	2095-2100	0	0	0	0.01	1.08	3.98	2	4.87	3.63	4.17
THA	1950-1955	0	0	0	0.03	1.61	8.95	2	7.83	2.57	1.75
	2020-2025	0	0	0	0.00	0.61	2.25	2	3.79	3.50	4.19
	2095-2100	0	0	0	0.00	0.57	1.25	2	2.87	3.79	5.07
TJK	1950-1955	0	0	0	0.03	1.24	6.15	2	7.13	3.27	3.04
	2020-2025	0	0	0	0.00	1.04	5.84	2	7.10	3.54	3.64
	2095-2100	0	0	0	0.00	0.90	2.64	2	4.24	3.88	6.26
TKL	1950-1955	0	0	0	0.06	1.83	11.36	2	8.35	2.95	2.23
	2020-2025	0	0	0	0.02	1.20	5.73	2	7.21	3.64	3.89
	2095-2100	0	0	0	0.00	0.77	2.09	2	3.85	3.81	4.81
TKM	1950-1955	0	0	0	0.03	1.26	6.45	2	6.98	3.01	2.35
	2020-2025	0	0	0	0.00	0.97	4.98	2	6.22	3.50	3.32
	2095-2100	0	0	0	0.00	0.95	2.88	2	4.22	3.80	5.29
TLS	1950-1955	0	0	0	0.02	1.32	5.30	2	5.53	2.25	1.18
	2020-2025	0	0	0	0.02	1.32	5.75	2	7.22	3.16	1.72
	2095-2100	0	0	0	0.00	0.83	2.34	2	3.91	3.80	5.16
TON	1950-1955	0	0	0	0.02	1.90	12.77	2	10.10	3.05	2.36
	2020-2025	0	0	0	0.01	1.24	7.75	2	8.23	3.38	2.98
	2095-2100	0	0	0	0.00	0.93	3.09	2	4.60	3.71	4.42

Table S2. Living kin for a female Focal aged 0-4. Country-level averages for selected years (values for 2095-2100 refer to the median of the 1,000 country-level projections). Relative codes: ggd = great-grandchildren, gd = grandchildren, d = children, n = niblings, s = siblings, c = cousins, m = parents, a = aunts/uncles, gm = grandparents, ggm = great-grandparents. *(continued)*

ISO	Year	ggd	gd	d	n	s	c	m	a	gm	ggm
TTO	1950-1955	0	0	0	0.03	1.64	9.76	2	8.07	3.25	2.98
	2020-2025	0	0	0	0.00	0.69	2.63	2	4.24	3.56	4.28
	2095-2100	0	0	0	0.00	0.66	1.56	2	3.22	3.80	5.11
TUN	1950-1955	0	0	0	0.03	1.58	8.24	2	7.08	2.69	1.84
	2020-2025	0	0	0	0.00	0.79	4.62	2	6.36	3.63	3.61
	2095-2100	0	0	0	0.00	0.72	1.88	2	3.55	3.85	4.95
TUV	1950-1955	0	0	0	0.01	1.38	5.73	2	6.08	2.59	1.72
	2020-2025	0	0	0	0.01	1.15	4.67	2	6.41	3.25	2.42
	2095-2100	0	0	0	0.00	0.93	3.10	2	4.41	3.52	3.48
TWN	1950-1955	0	0	0	0.04	2.10	15.54	2	10.51	3.13	2.00
	2020-2025	0	0	0	0.00	0.60	1.88	2	4.10	3.65	4.37
	2095-2100	0	0	0	0.00	0.57	1.30	2	2.89	3.72	3.33
TZA	1950-1955	0	0	0	0.06	1.48	7.61	2	6.96	2.87	2.54
	2020-2025	0	0	0	0.04	1.49	9.32	2	8.07	3.10	2.97
	2095-2100	0	0	0	0.01	1.12	4.32	2	4.97	3.70	4.57
UGA	1950-1955	0	0	0	0.07	1.64	8.62	2	7.02	2.73	2.31
	2020-2025	0	0	0	0.05	1.56	9.09	2	8.72	2.99	2.65
	2095-2100	0	0	0	0.01	0.94	3.35	2	4.36	3.54	3.72
UKR	1950-1955	0	0	0	0.01	0.71	2.03	2	4.04	3.33	3.12
	2020-2025	0	0	0	0.00	0.65	1.44	2	2.92	3.37	3.46
	2095-2100	0	0	0	0.00	0.64	1.57	2	3.04	3.60	3.88
URY	1950-1955	0	0	0	0.01	0.88	2.89	2	4.48	3.53	3.80
	2020-2025	0	0	0	0.01	0.75	2.46	2	4.13	3.69	4.46
	2095-2100	0	0	0	0.00	0.60	1.40	2	3.03	3.83	5.02
USA	1950-1955	0	0	0	0.01	1.11	4.49	2	5.47	3.57	4.10
	2020-2025	0	0	0	0.00	0.83	2.08	2	3.74	3.71	5.08
	2095-2100	0	0	0	0.00	0.65	1.50	2	3.26	3.80	5.37
UZB	1950-1955	0	0	0	0.02	1.28	6.41	2	7.67	3.40	3.55
	2020-2025	0	0	0	0.00	0.86	4.07	2	5.91	3.63	3.86
	2095-2100	0	0	0	0.00	0.86	2.57	2	4.06	3.79	4.99
VCT	1950-1955	0	0	0	0.05	1.90	13.60	2	9.81	3.18	3.23
	2020-2025	0	0	0	0.01	0.77	3.36	2	4.97	3.61	4.94
	2095-2100	0	0	0	0.00	0.68	1.69	2	3.29	3.73	4.82
VEN	1950-1955	0	0	0	0.04	1.80	11.65	2	9.12	3.08	2.94
	2020-2025	0	0	0	0.01	0.80	3.89	2	5.21	3.62	4.88
	2095-2100	0	0	0	0.00	0.77	1.93	2	3.44	3.81	5.52
VGB	1950-1955	0	0	0	0.05	1.60	9.23	2	7.95	3.28	3.30
	2020-2025	0	0	0	0.00	0.56	2.12	2	3.63	3.74	4.43
	2095-2100	0	0	0	0.00	0.53	1.00	2	2.58	3.84	4.73

Table S2. Living kin for a female Focal aged 0-4. Country-level averages for selected years (values for 2095-2100 refer to the median of the 1,000 country-level projections). Relative codes: ggd = great-grandchildren, gd = grandchildren, d = children, n = niblings, s = siblings, c = cousins, m = parents, a = aunts/uncles, gm = grandparents, ggm = great-grandparents. ***(continued)***

ISO	Year	ggd	gd	d	n	s	c	m	a	gm	ggm
VIR	1950-1955	0	0	0	0.03	1.43	7.63	2	7.02	3.25	3.31
	2020-2025	0	0	0	0.01	0.89	3.92	2	5.15	3.63	4.22
	2095-2100	0	0	0	0.00	0.69	1.74	2	3.66	3.86	5.75
VNM	1950-1955	0	0	0	0.02	1.63	8.53	2	7.08	2.59	1.61
	2020-2025	0	0	0	0.00	0.73	3.31	2	4.85	3.59	3.70
	2095-2100	0	0	0	0.00	0.72	1.84	2	3.51	3.77	4.79
VUT	1950-1955	0	0	0	0.03	1.88	11.71	2	9.16	2.83	2.28
	2020-2025	0	0	0	0.03	1.35	8.84	2	8.14	3.56	3.65
	2095-2100	0	0	0	0.01	1.06	4.06	2	4.99	3.80	5.03
WSM	1950-1955	0	0	0	0.05	2.05	14.64	2	10.39	2.99	2.47
	2020-2025	0	0	0	0.02	1.39	9.40	2	8.83	3.59	3.72
	2095-2100	0	0	0	0.01	1.05	3.85	2	4.92	3.84	5.44
YEM	1950-1955	0	0	0	0.04	1.43	6.80	2	6.45	2.69	1.78
	2020-2025	0	0	0	0.04	1.53	10.98	2	9.89	3.45	3.12
	2095-2100	0	0	0	0.00	0.88	2.92	2	4.19	3.62	3.66
ZAF	1950-1955	0	0	0	0.05	1.65	9.28	2	7.50	2.88	2.30
	2020-2025	0	0	0	0.01	0.82	4.08	2	5.15	2.87	2.87
	2095-2100	0	0	0	0.00	0.79	1.98	2	3.49	3.46	3.84
ZMB	1950-1955	0	0	0	0.07	1.58	8.84	2	7.60	3.11	3.06
	2020-2025	0	0	0	0.03	1.50	8.78	2	8.03	2.88	2.81
	2095-2100	0	0	0	0.01	1.04	3.76	2	4.72	3.75	4.73
ZWE	1950-1955	0	0	0	0.10	1.85	12.39	2	8.85	3.03	2.79
	2020-2025	0	0	0	0.01	1.08	6.57	2	6.36	2.55	2.44
	2095-2100	0	0	0	0.00	0.90	2.87	2	4.11	3.38	3.35

Table S3. Living kin for a female Focal aged 35-39. Country-level averages for selected years (values for 2095-2100 refer to the median of the 1,000 country-level projections). Relative codes: ggd = great-grandchildren, gd = grandchildren, d = children, n = niblings, s = siblings, c = cousins, m = parents, a = aunts/uncles, gm = grandparents, ggm = great-grandparents.

ISO	Year	ggd	gd	d	n	s	c	m	a	gm	ggm
ABW	1950-1955	0	0.01	4.11	12.50	4.33	39.21	1.24	6.28	0.44	0.01
	2020-2025	0	0.00	1.68	2.51	2.09	15.00	1.69	6.13	1.12	0.08
	2095-2100	0	0.00	1.09	0.96	1.38	4.54	1.85	3.03	1.37	0.10
AFG	1950-1955	0	0.04	3.13	6.38	2.68	17.09	0.72	2.87	0.18	0.01
	2020-2025	0	0.06	4.31	12.76	4.56	33.43	1.20	5.27	0.51	0.03
	2095-2100	0	0.00	2.39	3.88	2.17	10.92	1.65	4.13	1.12	0.11
AGO	1950-1955	0	0.03	2.84	5.60	2.67	15.85	1.04	3.51	0.43	0.03
	2020-2025	0	0.08	3.98	10.40	3.90	27.48	1.13	4.74	0.50	0.04
	2095-2100	0	0.01	2.81	4.81	2.49	14.43	1.57	4.53	1.02	0.12
AIA	1950-1955	0	0.08	4.74	16.33	4.92	50.54	1.30	7.45	0.62	0.04
	2020-2025	0	0.00	1.31	2.40	2.40	20.97	1.74	8.48	1.47	0.22
	2095-2100	0	0.00	1.18	1.15	1.45	4.06	1.87	2.66	1.75	0.32
ALB	1950-1955	0	0.00	3.28	7.78	3.71	27.81	1.30	5.58	0.45	0.01
	2020-2025	0	0.00	1.57	3.43	2.82	19.50	1.76	7.00	1.30	0.09
	2095-2100	0	0.00	1.17	1.29	1.63	5.18	1.92	3.12	2.10	0.35
AND	1950-1955	0	0.00	1.82	2.48	2.01	8.24	1.45	3.20	0.53	0.01
	2020-2025	0	0.00	1.09	1.28	1.69	6.95	1.86	4.12	1.46	0.09
	2095-2100	0	0.00	1.03	0.91	1.36	3.70	1.93	2.57	1.63	0.08
ARE	1950-1955	0	0.07	3.69	9.35	3.53	27.29	1.13	4.98	0.58	0.05
	2020-2025	0	0.00	1.56	6.40	4.56	31.34	1.71	8.35	1.19	0.12
	2095-2100	0	0.00	1.21	1.22	1.50	5.09	1.92	3.18	1.35	0.06
ARG	1950-1955	0	0.01	2.39	4.39	2.56	13.32	1.39	3.96	0.54	0.02
	2020-2025	0	0.01	1.91	3.63	2.63	12.97	1.63	4.45	1.10	0.12
	2095-2100	0	0.00	1.45	1.65	1.73	6.26	1.85	3.46	1.76	0.32
ARM	1950-1955	0	0.00	3.39	7.79	3.78	28.59	1.42	6.16	0.77	0.04
	2020-2025	0	0.00	1.51	2.43	2.05	12.21	1.64	5.26	0.97	0.05
	2095-2100	0	0.00	1.30	1.49	1.64	5.44	1.81	3.13	1.50	0.24
ASM	1950-1955	0	0.01	4.62	15.27	4.94	50.19	1.37	7.75	0.72	0.05
	2020-2025	0	0.00	2.81	7.62	4.03	40.19	1.57	8.86	0.86	0.05
	2095-2100	0	0.00	1.57	1.95	1.83	7.81	1.71	3.78	1.08	0.11
ATG	1950-1955	0	0.04	3.47	8.81	3.65	27.41	1.30	5.53	0.66	0.06
	2020-2025	0	0.00	1.55	1.99	2.09	12.79	1.75	5.75	1.75	0.35
	2095-2100	0	0.00	1.37	1.44	1.60	5.02	1.89	3.03	2.12	0.43
AUS	1950-1955	0	0.00	2.70	5.74	2.83	16.38	1.49	4.58	0.71	0.04
	2020-2025	0	0.00	1.65	2.25	2.14	11.40	1.82	5.04	1.80	0.22
	2095-2100	0	0.00	1.37	1.45	1.63	5.67	1.92	3.32	1.89	0.14
AUT	1950-1955	0	0.00	1.68	2.14	1.77	6.34	1.50	2.89	0.75	0.04
	2020-2025	0	0.00	1.37	1.35	1.59	6.06	1.80	3.53	1.71	0.23
	2095-2100	0	0.00	1.23	1.22	1.53	5.03	1.90	3.13	1.66	0.10

Table S3. Living kin for a female Focal aged 35-39. Country-level averages for selected years (values for 2095-2100 refer to the median of the 1,000 country-level projections). Relative codes: ggd = great-grandchildren, gd = grandchildren, d = children, n = niblings, s = siblings, c = cousins, m = parents, a = aunts/uncles, gm = grandparents, ggm = great-grandparents. ***(continued)***

ISO	Year	ggd	gd	d	n	s	c	m	a	gm	ggm
AZE	1950-1955	0	0.00	3.35	7.34	3.58	26.51	1.21	5.31	0.51	0.02
	2020-2025	0	0.00	1.65	3.30	2.36	16.42	1.57	6.30	0.76	0.04
	2095-2100	0	0.00	1.45	1.65	1.85	5.63	1.84	2.99	2.07	0.45
BDI	1950-1955	0	0.02	3.47	8.68	3.62	27.93	1.06	4.75	0.35	0.01
	2020-2025	0	0.02	4.06	10.71	4.34	32.02	1.18	5.02	0.41	0.02
	2095-2100	0	0.00	2.52	4.09	2.34	13.17	1.55	4.39	0.87	0.06
BEL	1950-1955	0	0.00	1.92	2.84	2.07	8.66	1.50	3.36	0.72	0.03
	2020-2025	0	0.00	1.65	1.77	1.78	7.11	1.79	3.62	1.61	0.15
	2095-2100	0	0.00	1.41	1.49	1.63	5.67	1.90	3.30	1.94	0.15
BEN	1950-1955	0	0.03	2.75	5.43	2.65	15.41	1.03	3.42	0.38	0.02
	2020-2025	0	0.05	3.77	10.52	4.21	27.89	1.36	5.29	0.71	0.06
	2095-2100	0	0.00	2.80	4.91	2.51	14.35	1.56	4.48	0.98	0.09
BES	1950-1955	0	0.02	4.16	12.80	4.57	42.20	1.44	7.29	0.71	0.05
	2020-2025	0	0.00	1.54	2.53	2.25	16.67	1.73	6.68	1.23	0.11
	2095-2100	0	0.00	1.40	1.51	1.63	5.28	1.88	3.10	1.65	0.19
BFA	1950-1955	0	0.04	2.70	5.03	2.50	13.96	1.01	3.24	0.34	0.01
	2020-2025	0	0.04	3.81	10.53	4.23	27.14	1.28	4.97	0.58	0.03
	2095-2100	0	0.00	2.41	3.79	2.16	10.80	1.57	3.96	0.96	0.08
BGD	1950-1955	0	0.15	3.23	7.51	3.25	21.92	1.28	4.89	0.60	0.04
	2020-2025	0	0.04	2.00	5.47	3.25	22.52	1.59	6.79	1.15	0.18
	2095-2100	0	0.00	1.70	2.00	1.68	5.84	1.90	3.31	2.46	0.79
BGR	1950-1955	0	0.00	1.87	2.71	1.87	7.26	1.54	3.15	0.99	0.09
	2020-2025	0	0.00	1.38	1.67	1.58	5.27	1.65	2.99	1.41	0.20
	2095-2100	0	0.00	1.25	1.49	1.68	5.32	1.78	2.99	1.42	0.20
BHR	1950-1955	0	0.12	3.49	8.69	3.41	25.15	1.17	4.84	0.59	0.05
	2020-2025	0	0.00	1.82	6.16	4.06	30.26	1.77	8.58	1.35	0.16
	2095-2100	0	0.00	1.47	1.77	1.70	6.14	1.93	3.50	1.90	0.21
BHS	1950-1955	0	0.03	3.52	9.14	3.60	27.43	1.23	5.22	0.60	0.04
	2020-2025	0	0.00	1.49	2.63	2.42	16.29	1.53	5.78	1.22	0.18
	2095-2100	0	0.00	1.24	1.18	1.45	4.22	1.75	2.58	1.57	0.23
BIH	1950-1955	0	0.00	3.12	6.58	3.40	23.45	1.31	5.27	0.54	0.02
	2020-2025	0	0.00	1.29	1.58	1.68	8.78	1.53	4.37	1.10	0.10
	2095-2100	0	0.00	1.18	1.17	1.49	4.53	1.88	2.88	1.63	0.19
BLR	1950-1955	0	0.00	2.03	2.86	2.12	9.23	1.35	3.33	0.58	0.02
	2020-2025	0	0.00	1.47	1.86	1.61	6.46	1.44	3.22	0.76	0.06
	2095-2100	0	0.00	1.39	1.49	1.62	5.68	1.75	3.12	1.35	0.17
BLZ	1950-1955	0	0.05	4.38	14.07	4.41	41.33	1.23	6.46	0.59	0.04
	2020-2025	0	0.02	2.32	6.54	3.87	33.41	1.57	8.15	1.37	0.22
	2095-2100	0	0.00	1.66	1.84	1.67	6.03	1.84	3.27	1.98	0.47

Table S3. Living kin for a female Focal aged 35-39. Country-level averages for selected years (values for 2095-2100 refer to the median of the 1,000 country-level projections). Relative codes: ggd = great-grandchildren, gd = grandchildren, d = children, n = niblings, s = siblings, c = cousins, m = parents, a = aunts/uncles, gm = grandparents, ggm = great-grandparents. ***(continued)***

ISO	Year	ggd	gd	d	n	s	c	m	a	gm	ggm
BMU	1950-1955	0	0.01	2.66	5.14	2.52	13.92	1.13	3.48	0.46	0.02
	2020-2025	0	0.00	1.48	1.67	1.80	9.36	1.74	4.59	1.55	0.20
	2095-2100	0	0.00	1.12	1.10	1.46	4.62	1.88	2.94	1.44	0.11
BOL	1950-1955	0	0.02	3.24	7.14	3.15	21.72	1.13	4.34	0.46	0.02
	2020-2025	0	0.02	2.44	6.00	3.45	23.53	1.42	5.76	0.64	0.04
	2095-2100	0	0.00	1.84	2.30	1.84	7.13	1.73	3.39	1.25	0.14
BRA	1950-1955	0	0.02	3.74	9.77	3.72	29.85	1.23	5.43	0.61	0.04
	2020-2025	0	0.02	1.61	3.20	2.44	17.78	1.57	6.40	1.08	0.09
	2095-2100	0	0.00	1.35	1.45	1.62	5.22	1.86	3.09	1.79	0.32
BRB	1950-1955	0	0.02	2.97	6.63	3.13	19.95	1.37	4.88	0.75	0.07
	2020-2025	0	0.00	1.41	1.74	1.77	10.45	1.72	5.27	1.58	0.21
	2095-2100	0	0.00	1.39	1.45	1.60	5.39	1.88	3.17	1.84	0.23
BRN	1950-1955	0	0.01	4.53	15.01	4.71	46.28	1.31	7.10	0.60	0.03
	2020-2025	0	0.00	1.77	4.57	3.50	30.31	1.65	8.61	1.06	0.09
	2095-2100	0	0.00	1.39	1.52	1.64	5.70	1.82	3.21	1.32	0.08
BTN	1950-1955	0	0.03	3.00	6.01	2.69	16.78	0.76	2.97	0.20	0.01
	2020-2025	0	0.01	1.95	5.84	3.73	22.20	1.44	5.62	0.64	0.04
	2095-2100	0	0.00	1.28	1.24	1.48	4.73	1.86	2.92	1.74	0.22
BWA	1950-1955	0	0.07	3.50	8.88	3.67	28.53	1.06	4.94	0.46	0.03
	2020-2025	0	0.01	2.42	6.49	3.38	26.68	1.07	5.78	0.53	0.06
	2095-2100	0	0.00	1.85	2.39	1.88	7.41	1.54	3.20	0.85	0.07
CAF	1950-1955	0	0.07	2.85	5.68	2.52	14.75	0.93	3.11	0.38	0.03
	2020-2025	0	0.11	3.66	9.68	3.66	24.47	1.03	4.22	0.53	0.06
	2095-2100	0	0.01	2.72	4.42	2.23	11.93	1.42	3.90	0.86	0.11
CAN	1950-1955	0	0.00	2.95	6.79	3.17	20.39	1.52	5.20	0.86	0.06
	2020-2025	0	0.00	1.47	1.65	1.79	10.01	1.81	5.08	1.74	0.22
	2095-2100	0	0.00	1.22	1.18	1.51	4.90	1.91	3.12	1.78	0.12
CHE	1950-1955	0	0.00	1.95	2.87	2.09	8.91	1.52	3.43	0.65	0.02
	2020-2025	0	0.00	1.35	1.42	1.72	6.75	1.82	3.70	1.64	0.14
	2095-2100	0	0.00	1.30	1.31	1.57	4.98	1.94	3.07	1.79	0.08
CHL	1950-1955	0	0.02	3.06	7.04	3.43	23.90	1.24	5.05	0.47	0.02
	2020-2025	0	0.01	1.51	2.56	2.35	13.96	1.75	5.62	1.48	0.22
	2095-2100	0	0.00	1.28	1.35	1.56	5.07	1.90	3.13	1.94	0.35
CHN	1950-1955	0	0.01	3.86	10.91	3.96	33.49	0.96	4.97	0.23	0.01
	2020-2025	0	0.00	1.69	2.71	2.02	16.64	1.72	7.19	1.22	0.10
	2095-2100	0	0.00	1.16	1.08	1.39	4.58	1.90	3.09	1.83	0.26
CIV	1950-1955	0	0.06	3.21	7.06	3.04	20.32	1.04	4.01	0.42	0.03
	2020-2025	0	0.06	3.52	11.24	4.34	33.53	1.17	5.79	0.58	0.06
	2095-2100	0	0.01	2.55	4.13	2.36	12.45	1.50	4.05	0.90	0.10

Table S3. Living kin for a female Focal aged 35-39. Country-level averages for selected years (values for 2095-2100 refer to the median of the 1,000 country-level projections). Relative codes: ggd = great-grandchildren, gd = grandchildren, d = children, n = niblings, s = siblings, c = cousins, m = parents, a = aunts/uncles, gm = grandparents, ggm = great-grandparents. ***(continued)***

ISO	Year	ggd	gd	d	n	s	c	m	a	gm	ggm
CMR	1950-1955	0	0.05	2.51	4.40	2.31	11.90	1.08	3.12	0.52	0.04
	2020-2025	0	0.06	3.62	10.00	4.09	25.68	1.28	4.97	0.77	0.10
	2095-2100	0	0.00	2.54	4.06	2.32	12.37	1.55	4.14	1.01	0.12
COD	1950-1955	0	0.07	2.95	6.17	2.90	18.03	1.09	3.97	0.51	0.04
	2020-2025	0	0.05	4.27	11.38	4.22	29.58	1.28	5.13	0.68	0.07
	2095-2100	0	0.00	3.26	6.13	2.73	18.01	1.54	5.06	0.86	0.08
COG	1950-1955	0	0.04	3.24	7.30	3.06	20.92	1.00	3.93	0.42	0.03
	2020-2025	0	0.04	3.30	8.86	3.83	29.07	1.20	5.61	0.60	0.05
	2095-2100	0	0.00	2.46	3.92	2.33	12.22	1.53	4.11	0.92	0.09
COK	1950-1955	0	0.02	4.17	11.54	3.69	31.84	0.85	4.41	0.32	0.02
	2020-2025	0	0.01	2.10	5.27	3.47	30.48	1.57	8.13	1.15	0.14
	2095-2100	0	0.00	1.67	2.05	1.81	6.74	1.81	3.45	1.86	0.40
COL	1950-1955	0	0.04	4.02	11.51	4.08	35.58	1.20	5.89	0.62	0.05
	2020-2025	0	0.02	1.69	3.53	2.61	21.44	1.62	7.39	1.39	0.16
	2095-2100	0	0.00	1.45	1.61	1.68	5.18	1.90	3.05	2.24	0.60
COM	1950-1955	0	0.04	2.98	6.12	2.79	17.41	1.01	3.57	0.38	0.02
	2020-2025	0	0.03	3.23	10.00	4.55	31.97	1.32	5.94	0.53	0.03
	2095-2100	0	0.00	2.29	3.48	2.31	12.36	1.58	4.30	0.76	0.06
CPV	1950-1955	0	0.02	3.86	10.77	4.10	35.57	1.07	5.46	0.36	0.01
	2020-2025	0	0.02	2.01	5.70	3.91	32.44	1.55	8.04	0.99	0.07
	2095-2100	0	0.00	1.58	1.75	1.71	5.98	1.87	3.30	2.07	0.48
CRI	1950-1955	0	0.03	4.32	13.62	4.59	43.55	1.35	7.09	0.71	0.05
	2020-2025	0	0.01	1.68	3.61	2.77	22.79	1.75	7.90	1.69	0.24
	2095-2100	0	0.00	1.29	1.35	1.59	5.18	1.91	3.18	2.19	0.52
CUB	1950-1955	0	0.06	2.78	6.01	2.89	16.99	1.56	4.88	1.12	0.14
	2020-2025	0	0.01	1.33	1.79	1.75	9.83	1.75	5.24	1.89	0.55
	2095-2100	0	0.00	1.31	1.35	1.55	5.27	1.87	3.21	2.02	0.39
CYM	1950-1955	0	0.05	3.07	6.74	3.05	19.78	1.27	4.58	0.66	0.05
	2020-2025	0	0.00	1.45	2.01	1.97	12.30	1.67	5.69	1.23	0.15
	2095-2100	0	0.00	1.00	0.92	1.37	4.36	1.80	2.74	0.84	0.03
CYP	1950-1955	0	0.00	2.48	4.53	2.66	14.65	1.26	3.91	0.43	0.01
	2020-2025	0	0.00	1.35	1.85	2.24	11.09	1.80	4.80	1.55	0.15
	2095-2100	0	0.00	0.97	0.93	1.43	4.61	1.89	3.03	1.26	0.07
CZE	1950-1955	0	0.00	2.26	4.02	2.33	11.07	1.52	3.83	0.83	0.04
	2020-2025	0	0.00	1.43	1.79	1.80	6.76	1.75	3.46	1.56	0.22
	2095-2100	0	0.00	1.46	1.65	1.68	5.69	1.88	3.22	1.66	0.13
DEU	1950-1955	0	0.00	1.73	2.21	1.82	6.70	1.56	3.07	0.90	0.06
	2020-2025	0	0.00	1.34	1.25	1.54	5.55	1.78	3.37	1.72	0.22
	2095-2100	0	0.00	1.24	1.25	1.56	5.07	1.87	3.10	1.63	0.11

Table S3. Living kin for a female Focal aged 35-39. Country-level averages for selected years (values for 2095-2100 refer to the median of the 1,000 country-level projections). Relative codes: ggd = great-grandchildren, gd = grandchildren, d = children, n = niblings, s = siblings, c = cousins, m = parents, a = aunts/uncles, gm = grandparents, ggm = great-grandparents. ***(continued)***

ISO	Year	ggd	gd	d	n	s	c	m	a	gm	ggm
DJI	1950-1955	0	0.02	3.51	8.27	3.59	27.58	1.06	4.77	0.35	0.01
	2020-2025	0	0.01	2.36	6.72	4.04	29.83	1.26	6.00	0.50	0.02
	2095-2100	0	0.00	1.79	2.19	1.81	6.95	1.58	3.14	0.73	0.03
DMA	1950-1955	0	0.02	3.03	6.56	2.99	19.26	1.16	4.18	0.52	0.04
	2020-2025	0	0.01	1.58	2.76	2.63	17.00	1.63	6.15	1.12	0.11
	2095-2100	0	0.00	1.41	1.51	1.63	4.80	1.83	2.79	1.65	0.26
DNK	1950-1955	0	0.00	2.12	3.53	2.24	10.18	1.63	3.85	1.02	0.07
	2020-2025	0	0.00	1.61	1.81	1.90	7.95	1.77	3.81	1.62	0.19
	2095-2100	0	0.00	1.46	1.58	1.71	6.03	1.92	3.42	1.83	0.07
DOM	1950-1955	0	0.04	4.29	12.59	4.33	39.90	1.22	6.34	0.58	0.04
	2020-2025	0	0.03	2.10	4.70	2.79	24.01	1.59	7.53	1.28	0.22
	2095-2100	0	0.00	1.66	1.95	1.77	6.66	1.83	3.48	1.99	0.55
DZA	1950-1955	0	0.03	4.01	10.89	3.96	34.04	1.15	5.53	0.47	0.03
	2020-2025	0	0.00	2.10	6.35	4.16	31.55	1.67	7.91	0.94	0.07
	2095-2100	0	0.00	1.92	2.62	1.97	8.23	1.91	4.00	1.82	0.22
ECU	1950-1955	0	0.04	4.11	12.17	4.30	38.62	1.25	6.34	0.58	0.04
	2020-2025	0	0.02	2.13	5.23	3.19	26.99	1.65	7.93	1.35	0.19
	2095-2100	0	0.00	1.57	1.79	1.68	6.21	1.88	3.44	2.15	0.55
EGY	1950-1955	0	0.08	3.26	7.64	3.30	22.82	1.22	4.80	0.55	0.04
	2020-2025	0	0.01	2.67	7.39	3.65	26.04	1.51	6.36	0.80	0.06
	2095-2100	0	0.00	2.02	2.85	2.04	8.95	1.83	4.04	1.77	0.28
ERI	1950-1955	0	0.04	2.92	5.59	2.75	16.86	0.92	3.39	0.29	0.01
	2020-2025	0	0.02	3.29	9.01	3.98	26.14	1.31	5.16	0.59	0.04
	2095-2100	0	0.00	2.17	3.12	2.08	9.51	1.76	4.10	1.59	0.22
ESH	1950-1955	0	0.04	3.25	7.29	2.98	20.27	0.86	3.48	0.24	0.01
	2020-2025	0	0.01	2.10	5.33	3.47	22.26	1.38	5.32	0.53	0.03
	2095-2100	0	0.00	1.65	1.98	1.77	6.72	1.79	3.32	1.21	0.09
ESP	1950-1955	0	0.00	1.93	2.71	2.16	9.45	1.46	3.47	0.58	0.02
	2020-2025	0	0.00	1.10	1.36	1.81	7.53	1.77	4.19	1.46	0.10
	2095-2100	0	0.00	1.12	1.04	1.43	4.03	1.90	2.69	1.61	0.08
EST	1950-1955	0	0.00	1.92	2.64	2.02	8.39	1.39	3.20	0.66	0.03
	2020-2025	0	0.00	1.52	1.83	1.71	5.86	1.54	2.83	1.01	0.10
	2095-2100	0	0.00	1.38	1.51	1.64	5.60	1.81	3.14	1.47	0.19
ETH	1950-1955	0	0.02	3.25	7.53	3.34	23.93	0.98	4.21	0.30	0.01
	2020-2025	0	0.04	3.63	9.73	4.14	29.46	1.27	5.25	0.58	0.04
	2095-2100	0	0.00	2.33	3.62	2.19	10.79	1.71	4.22	1.32	0.16
FIN	1950-1955	0	0.00	2.45	4.61	2.67	14.58	1.35	4.07	0.52	0.02
	2020-2025	0	0.00	1.55	1.76	1.81	8.22	1.73	3.96	1.35	0.12
	2095-2100	0	0.00	1.26	1.25	1.51	4.85	1.90	3.04	1.81	0.12

Table S3. Living kin for a female Focal aged 35-39. Country-level averages for selected years (values for 2095-2100 refer to the median of the 1,000 country-level projections). Relative codes: ggd = great-grandchildren, gd = grandchildren, d = children, n = niblings, s = siblings, c = cousins, m = parents, a = aunts/uncles, gm = grandparents, ggm = great-grandparents. ***(continued)***

ISO	Year	ggd	gd	d	n	s	c	m	a	gm	ggm
FJI	1950-1955	0	0.08	4.58	15.30	4.78	47.31	1.05	6.53	0.25	0.00
	2020-2025	0	0.00	2.38	5.31	3.18	28.19	1.46	7.15	0.79	0.06
	2095-2100	0	0.00	1.77	2.23	1.90	7.67	1.60	3.38	0.90	0.07
FLK	1950-1955	0	0.00	2.15	3.33	2.01	8.86	1.21	2.90	0.56	0.03
	2020-2025	0	0.00	1.33	1.51	1.64	6.90	1.67	3.77	1.16	0.09
	2095-2100	0	0.00	1.35	1.45	1.60	4.93	1.92	3.00	1.86	0.20
FRA	1950-1955	0	0.00	2.34	4.13	2.45	12.27	1.48	3.98	0.77	0.04
	2020-2025	0	0.00	1.76	2.23	2.01	9.21	1.74	4.17	1.67	0.21
	2095-2100	0	0.00	1.50	1.67	1.73	6.23	1.87	3.42	2.00	0.21
FRO	1950-1955	0	0.00	2.83	6.18	3.06	19.33	1.32	4.55	0.47	0.02
	2020-2025	0	0.00	2.32	3.88	2.67	15.99	1.80	5.54	1.41	0.13
	2095-2100	0	0.00	2.17	3.03	2.18	9.69	1.93	4.39	1.99	0.18
FSM	1950-1955	0	0.02	4.20	12.54	4.31	39.64	1.14	6.00	0.44	0.02
	2020-2025	0	0.01	2.62	8.15	4.46	40.41	1.55	8.66	0.82	0.05
	2095-2100	0	0.00	1.80	2.23	1.90	7.72	1.77	3.65	1.30	0.12
GAB	1950-1955	0	0.03	2.09	3.16	1.94	8.34	1.13	2.65	0.54	0.05
	2020-2025	0	0.04	3.02	7.84	3.78	20.11	1.39	4.54	0.83	0.10
	2095-2100	0	0.00	2.14	3.05	2.04	9.38	1.61	3.71	1.10	0.13
GBR	1950-1955	0	0.00	1.89	2.81	2.00	8.12	1.51	3.24	0.67	0.03
	2020-2025	0	0.00	1.63	1.93	1.88	7.81	1.81	3.87	1.68	0.19
	2095-2100	0	0.00	1.34	1.43	1.62	5.61	1.91	3.32	1.89	0.19
GEO	1950-1955	0	0.00	2.15	3.23	2.21	10.21	1.28	3.35	0.59	0.03
	2020-2025	0	0.00	1.63	2.33	1.81	7.61	1.54	3.53	0.92	0.05
	2095-2100	0	0.00	1.50	1.92	1.91	7.34	1.73	3.54	1.25	0.16
GGY	1950-1955	0	0.00	1.93	3.14	2.05	8.55	1.56	3.35	0.69	0.02
	2020-2025	0	0.00	1.36	1.44	1.66	6.17	1.86	3.54	1.68	0.17
	2095-2100	0	0.00	1.24	1.24	1.55	4.87	1.93	3.07	1.90	0.20
GHA	1950-1955	0	0.06	3.58	9.10	3.69	29.02	1.14	5.16	0.50	0.03
	2020-2025	0	0.02	2.99	8.04	3.95	31.10	1.37	6.40	0.71	0.06
	2095-2100	0	0.00	2.20	3.12	2.07	9.55	1.60	3.75	0.99	0.08
GIN	1950-1955	0	0.06	2.71	5.25	2.60	14.87	1.06	3.44	0.43	0.03
	2020-2025	0	0.07	3.45	8.31	3.77	22.97	1.34	4.70	0.71	0.07
	2095-2100	0	0.00	2.33	3.45	2.08	9.88	1.57	3.76	1.00	0.11
GLP	1950-1955	0	0.01	3.80	10.57	3.88	32.75	0.92	4.74	0.24	0.01
	2020-2025	0	0.00	1.89	3.02	2.49	20.12	1.70	7.21	1.45	0.21
	2095-2100	0	0.00	1.55	1.71	1.79	6.68	1.87	3.60	1.95	0.24
GMB	1950-1955	0	0.11	2.74	5.20	2.48	13.79	1.06	3.36	0.51	0.04
	2020-2025	0	0.04	3.94	9.74	4.17	25.93	1.38	4.95	0.86	0.11
	2095-2100	0	0.00	2.30	3.48	2.25	12.22	1.59	4.43	0.90	0.07

Table S3. Living kin for a female Focal aged 35-39. Country-level averages for selected years (values for 2095-2100 refer to the median of the 1,000 country-level projections). Relative codes: ggd = great-grandchildren, gd = grandchildren, d = children, n = niblings, s = siblings, c = cousins, m = parents, a = aunts/uncles, gm = grandparents, ggm = great-grandparents. ***(continued)***

ISO	Year	ggd	gd	d	n	s	c	m	a	gm	ggm
GNB	1950-1955	0	0.03	2.75	5.39	2.59	14.97	1.03	3.30	0.34	0.01
	2020-2025	0	0.05	3.26	7.90	3.75	22.02	1.19	4.09	0.45	0.03
	2095-2100	0	0.00	2.19	3.11	2.02	9.37	1.52	3.63	0.87	0.08
GNQ	1950-1955	0	0.05	2.90	6.10	2.78	17.01	1.08	3.73	0.45	0.03
	2020-2025	0	0.08	3.38	8.34	3.74	23.42	1.31	4.73	0.66	0.06
	2095-2100	0	0.01	2.10	2.88	1.93	8.87	1.56	3.54	1.03	0.12
GRC	1950-1955	0	0.00	1.92	2.66	2.12	9.05	1.50	3.50	0.71	0.03
	2020-2025	0	0.00	1.24	1.41	1.78	6.60	1.78	3.59	1.64	0.16
	2095-2100	0	0.00	1.07	1.00	1.45	4.40	1.87	2.88	1.47	0.10
GRD	1950-1955	0	0.05	3.93	10.88	4.00	33.41	1.23	6.00	0.66	0.06
	2020-2025	0	0.01	1.85	3.83	3.09	24.14	1.62	7.52	1.41	0.25
	2095-2100	0	0.00	1.55	1.80	1.73	6.15	1.82	3.30	1.70	0.29
GRL	1950-1955	0	0.01	3.76	9.55	3.74	30.40	1.01	4.89	0.39	0.02
	2020-2025	0	0.01	2.01	2.98	2.09	16.75	1.54	6.14	0.88	0.07
	2095-2100	0	0.00	1.56	1.81	1.74	6.34	1.75	3.24	1.39	0.20
GTM	1950-1955	0	0.05	3.78	9.86	3.68	29.29	1.10	5.09	0.53	0.04
	2020-2025	0	0.03	2.63	8.06	4.19	32.70	1.46	6.98	1.13	0.19
	2095-2100	0	0.00	1.75	2.08	1.74	6.40	1.82	3.31	1.99	0.49
GUF	1950-1955	0	0.02	3.17	7.66	3.05	20.56	1.07	3.97	0.39	0.02
	2020-2025	0	0.02	3.06	6.89	3.36	24.18	1.68	6.13	1.06	0.09
	2095-2100	0	0.00	2.23	3.55	2.52	13.40	1.88	5.21	1.81	0.25
GUM	1950-1955	0	0.02	4.22	13.76	4.59	43.73	1.02	6.07	0.15	0.00
	2020-2025	0	0.00	2.65	5.56	2.97	25.87	1.61	7.26	1.42	0.22
	2095-2100	0	0.00	1.76	2.24	2.00	8.91	1.83	4.19	1.93	0.40
GUY	1950-1955	0	0.07	4.21	12.92	4.15	37.07	1.06	5.62	0.39	0.02
	2020-2025	0	0.02	2.21	4.55	2.74	23.89	1.40	6.85	0.94	0.14
	2095-2100	0	0.00	1.77	2.17	1.83	6.94	1.66	3.23	1.35	0.24
HKG	1950-1955	0	0.00	3.40	8.66	3.57	26.50	1.14	4.93	0.27	0.00
	2020-2025	0	0.00	1.02	1.28	1.80	12.42	1.83	7.02	1.60	0.14
	2095-2100	0	0.00	0.79	0.57	1.07	2.48	1.91	2.16	1.55	0.09
HND	1950-1955	0	0.05	4.19	12.00	4.07	36.01	1.15	5.71	0.52	0.03
	2020-2025	0	0.03	2.50	7.98	4.24	36.91	1.57	8.45	0.92	0.08
	2095-2100	0	0.00	1.77	2.12	1.76	6.47	1.84	3.35	1.86	0.37
HRV	1950-1955	0	0.00	2.09	3.17	2.15	9.54	1.40	3.42	0.67	0.03
	2020-2025	0	0.00	1.46	1.61	1.72	6.21	1.69	3.20	1.32	0.14
	2095-2100	0	0.00	1.14	1.12	1.52	5.04	1.85	3.10	1.34	0.09
HTI	1950-1955	0	0.03	2.90	6.20	3.10	20.24	1.04	4.05	0.34	0.02
	2020-2025	0	0.01	2.42	5.97	3.56	23.41	1.30	5.27	0.52	0.03
	2095-2100	0	0.00	1.85	2.27	1.82	7.12	1.59	3.20	0.83	0.06

Table S3. Living kin for a female Focal aged 35-39. Country-level averages for selected years (values for 2095-2100 refer to the median of the 1,000 country-level projections). Relative codes: ggd = great-grandchildren, gd = grandchildren, d = children, n = niblings, s = siblings, c = cousins, m = parents, a = aunts/uncles, gm = grandparents, ggm = great-grandparents. ***(continued)***

ISO	Year	ggd	gd	d	n	s	c	m	a	gm	ggm
HUN	1950-1955	0	0.00	1.99	3.03	2.04	8.55	1.50	3.36	0.85	0.05
	2020-2025	0	0.00	1.28	1.55	1.75	5.85	1.56	2.89	1.16	0.15
	2095-2100	0	0.00	1.29	1.38	1.59	4.95	1.80	2.90	1.37	0.12
IDN	1950-1955	0	0.05	2.85	5.52	2.70	16.01	1.15	3.82	0.57	0.04
	2020-2025	0	0.01	1.99	4.13	2.83	17.41	1.56	5.66	0.94	0.09
	2095-2100	0	0.00	1.70	2.04	1.76	6.42	1.76	3.20	1.39	0.14
IMN	1950-1955	0	0.00	1.78	2.38	1.75	6.49	1.45	2.79	0.81	0.05
	2020-2025	0	0.00	1.60	2.05	1.86	7.18	1.74	3.55	1.42	0.16
	2095-2100	0	0.00	1.35	1.39	1.58	5.03	1.91	3.08	2.13	0.32
IND	1950-1955	0	0.05	3.28	7.79	3.24	22.47	1.16	4.55	0.53	0.04
	2020-2025	0	0.01	2.40	5.29	2.97	20.02	1.55	5.83	1.04	0.13
	2095-2100	0	0.00	1.62	1.90	1.78	6.78	1.83	3.53	1.81	0.34
IRL	1950-1955	0	0.00	2.64	5.44	3.13	19.80	1.42	4.86	0.42	0.01
	2020-2025	0	0.00	1.58	2.73	2.50	14.98	1.78	5.78	1.08	0.04
	2095-2100	0	0.00	1.37	1.48	1.68	5.90	1.93	3.41	1.68	0.09
IRN	1950-1955	0	0.01	3.69	9.68	3.77	30.12	1.16	5.29	0.45	0.02
	2020-2025	0	0.00	1.55	4.76	3.51	25.77	1.60	7.54	0.90	0.06
	2095-2100	0	0.00	1.41	1.57	1.65	5.64	1.89	3.23	1.75	0.26
IRQ	1950-1955	0	0.02	3.67	9.58	3.59	28.00	1.08	4.76	0.38	0.02
	2020-2025	0	0.01	3.17	11.08	4.69	38.70	1.36	7.13	0.68	0.04
	2095-2100	0	0.00	2.22	3.42	2.19	10.61	1.79	4.36	1.52	0.19
ISL	1950-1955	0	0.00	3.06	7.23	3.32	22.43	1.59	5.64	1.08	0.10
	2020-2025	0	0.00	1.79	2.60	2.31	13.97	1.85	5.76	1.87	0.27
	2095-2100	0	0.00	1.38	1.54	1.66	6.10	1.94	3.64	1.73	0.11
ISR	1950-1955	0	0.01	3.74	10.79	3.94	31.49	1.56	6.59	0.89	0.06
	2020-2025	0	0.00	2.62	5.41	3.21	23.59	1.81	6.92	1.72	0.27
	2095-2100	0	0.00	2.13	3.30	2.34	11.74	1.91	4.83	2.11	0.34
ITA	1950-1955	0	0.00	1.79	2.40	1.99	7.95	1.51	3.26	0.69	0.02
	2020-2025	0	0.00	1.17	1.23	1.65	6.16	1.81	3.65	1.59	0.14
	2095-2100	0	0.00	1.07	1.00	1.44	4.22	1.92	2.84	1.58	0.06
JAM	1950-1955	0	0.03	2.91	6.37	2.99	18.57	1.40	4.74	0.87	0.09
	2020-2025	0	0.01	1.48	3.16	2.81	18.67	1.65	6.77	1.32	0.19
	2095-2100	0	0.00	1.24	1.17	1.47	4.17	1.77	2.56	1.40	0.20
JEY	1950-1955	0	0.00	1.76	2.41	1.86	7.01	1.59	3.13	0.89	0.05
	2020-2025	0	0.00	1.44	1.47	1.61	6.05	1.83	3.47	1.58	0.15
	2095-2100	0	0.00	1.30	1.31	1.53	4.86	1.93	3.04	1.80	0.16
JOR	1950-1955	0	0.02	4.18	11.70	4.23	38.25	1.06	5.72	0.42	0.02
	2020-2025	0	0.00	2.96	10.67	5.08	48.73	1.66	10.14	1.03	0.08
	2095-2100	0	0.00	1.89	2.57	2.06	9.03	1.89	4.23	1.91	0.25

Table S3. **Living kin for a female Focal aged 35-39. Country-level averages for selected years (values for 2095-2100 refer to the median of the 1,000 country-level projections). Relative codes: ggd = great-grandchildren, gd = grandchildren, d = children, n = niblings, s = siblings, c = cousins, m = parents, a = aunts/uncles, gm = grandparents, ggm = great-grandparents.** ***(continued)***

ISO	Year	ggd	gd	d	n	s	c	m	a	gm	ggm
JPN	1950-1955	0	0.00	2.40	3.99	2.51	12.99	1.43	4.04	0.62	0.02
	2020-2025	0	0.00	1.27	1.55	1.84	6.97	1.82	3.79	1.71	0.16
	2095-2100	0	0.00	1.10	1.03	1.46	4.49	1.90	2.94	1.66	0.09
KAZ	1950-1955	0	0.00	3.25	7.31	3.22	22.21	1.22	4.77	0.58	0.03
	2020-2025	0	0.00	2.19	3.90	2.28	14.64	1.36	4.79	0.65	0.04
	2095-2100	0	0.00	2.08	3.30	2.34	11.02	1.70	4.19	1.25	0.14
KEN	1950-1955	0	0.10	3.67	9.32	3.51	27.26	1.06	4.71	0.50	0.04
	2020-2025	0	0.04	3.12	10.20	4.46	39.08	1.20	7.02	0.78	0.11
	2095-2100	0	0.00	2.16	3.13	2.01	8.70	1.56	3.49	1.10	0.14
KGZ	1950-1955	0	0.00	3.24	7.34	3.27	22.62	1.16	4.71	0.52	0.03
	2020-2025	0	0.00	2.50	6.09	3.13	22.59	1.42	6.01	0.71	0.04
	2095-2100	0	0.00	2.05	3.12	2.05	9.08	1.76	3.94	1.63	0.27
KHM	1950-1955	0	0.01	3.64	8.82	3.38	25.62	0.98	4.27	0.32	0.01
	2020-2025	0	0.00	2.25	4.98	3.26	19.87	1.45	4.92	0.62	0.05
	2095-2100	0	0.00	1.82	2.26	1.76	6.25	1.80	3.13	1.95	0.37
KIR	1950-1955	0	0.02	3.77	10.15	3.78	30.81	1.09	5.08	0.40	0.02
	2020-2025	0	0.01	2.91	7.12	3.78	30.30	1.46	6.74	0.71	0.04
	2095-2100	0	0.00	2.28	3.34	2.21	10.22	1.68	4.01	0.97	0.07
KNA	1950-1955	0	0.08	4.62	16.32	4.57	44.78	1.22	6.49	0.53	0.03
	2020-2025	0	0.01	1.54	2.65	2.44	20.37	1.58	7.18	1.09	0.12
	2095-2100	0	0.00	1.34	1.37	1.56	4.48	1.75	2.59	1.51	0.24
KOR	1950-1955	0	0.00	3.26	6.35	2.40	15.37	0.61	2.24	0.11	0.00
	2020-2025	0	0.00	1.19	1.40	1.98	13.69	1.78	6.60	1.34	0.08
	2095-2100	0	0.00	0.92	0.65	1.19	3.11	1.91	2.38	1.45	0.05
KWT	1950-1955	0	0.04	4.40	14.67	4.38	40.98	1.31	6.53	0.60	0.03
	2020-2025	0	0.00	1.98	7.04	4.16	37.34	1.78	9.66	1.26	0.11
	2095-2100	0	0.00	1.78	2.36	1.99	7.30	1.95	3.67	2.53	0.36
LAO	1950-1955	0	0.03	3.26	7.52	3.21	22.50	1.01	4.13	0.34	0.01
	2020-2025	0	0.03	2.42	6.93	3.83	25.50	1.40	5.78	0.62	0.04
	2095-2100	0	0.00	1.85	2.42	1.85	6.66	1.80	3.24	1.73	0.29
LBN	1950-1955	0	0.03	4.15	12.89	4.60	42.60	1.43	7.36	0.77	0.05
	2020-2025	0	0.00	1.86	4.18	3.06	25.59	1.71	7.93	1.25	0.12
	2095-2100	0	0.00	1.62	1.94	1.77	6.37	1.91	3.50	2.19	0.39
LBR	1950-1955	0	0.07	3.03	6.40	2.91	18.58	1.08	3.92	0.45	0.03
	2020-2025	0	0.06	3.28	8.28	3.73	24.89	1.26	5.03	0.64	0.06
	2095-2100	0	0.00	2.19	3.16	2.06	9.73	1.59	3.77	1.11	0.14
LBY	1950-1955	0	0.01	3.14	6.57	2.97	19.60	1.01	3.76	0.34	0.01
	2020-2025	0	0.00	1.96	7.01	4.85	33.17	1.55	7.94	0.76	0.04
	2095-2100	0	0.00	1.64	1.92	1.78	6.53	1.80	3.37	1.42	0.09

Table S3. Living kin for a female Focal aged 35-39. Country-level averages for selected years (values for 2095-2100 refer to the median of the 1,000 country-level projections). Relative codes: ggd = great-grandchildren, gd = grandchildren, d = children, n = niblings, s = siblings, c = cousins, m = parents, a = aunts/uncles, gm = grandparents, ggm = great-grandparents. ***(continued)***

ISO	Year	ggd	gd	d	n	s	c	m	a	gm	ggm
LCA	1950-1955	0	0.04	3.70	9.67	3.64	28.37	1.26	5.41	0.61	0.04
	2020-2025	0	0.00	1.31	3.23	3.13	23.46	1.64	7.87	1.18	0.13
	2095-2100	0	0.00	1.26	1.25	1.50	4.14	1.82	2.57	1.56	0.23
LIE	1950-1955	0	0.00	1.99	2.95	2.00	8.48	1.28	2.90	0.45	0.01
	2020-2025	0	0.00	1.32	1.50	1.73	6.82	1.83	3.74	1.41	0.11
	2095-2100	0	0.00	1.26	1.23	1.53	4.78	1.95	3.02	1.88	0.14
LKA	1950-1955	0	0.03	3.91	10.97	3.98	33.36	1.33	6.07	0.61	0.03
	2020-2025	0	0.00	1.97	3.50	2.58	19.95	1.54	6.59	0.89	0.07
	2095-2100	0	0.00	1.61	1.82	1.75	6.28	1.87	3.39	1.86	0.21
LSO	1950-1955	0	0.03	3.15	7.09	3.25	22.56	1.01	4.22	0.37	0.02
	2020-2025	0	0.01	2.24	5.88	3.02	20.44	0.76	3.83	0.36	0.04
	2095-2100	0	0.00	1.86	2.25	1.68	6.16	1.30	2.50	0.65	0.06
LTU	1950-1955	0	0.00	2.26	3.62	2.42	12.05	1.40	3.85	0.69	0.03
	2020-2025	0	0.00	1.52	1.85	1.69	7.11	1.44	3.31	0.88	0.09
	2095-2100	0	0.00	1.36	1.45	1.64	5.88	1.74	3.20	1.20	0.12
LUX	1950-1955	0	0.00	1.85	2.52	1.91	7.53	1.47	3.09	0.71	0.02
	2020-2025	0	0.00	1.43	1.44	1.70	6.31	1.77	3.33	1.51	0.14
	2095-2100	0	0.00	1.20	1.19	1.50	4.77	1.88	2.98	1.59	0.13
LVA	1950-1955	0	0.00	1.64	1.92	1.77	6.37	1.42	2.82	0.68	0.03
	2020-2025	0	0.00	1.43	1.64	1.60	4.99	1.45	2.50	0.84	0.08
	2095-2100	0	0.00	1.34	1.43	1.58	5.17	1.74	2.89	1.29	0.16
MAC	1950-1955	0	0.00	4.07	12.59	4.50	41.59	1.29	6.64	0.39	0.01
	2020-2025	0	0.00	0.93	0.97	1.51	11.23	1.84	7.01	1.54	0.10
	2095-2100	0	0.00	1.21	1.16	1.43	3.84	1.94	2.61	2.05	0.21
MAF	1950-1955	0	0.02	4.40	15.04	4.99	50.25	1.43	7.88	0.66	0.04
	2020-2025	0	0.00	2.19	5.41	3.35	31.27	1.75	8.97	1.19	0.10
	2095-2100	0	0.00	1.70	2.21	1.98	8.38	1.90	4.13	1.72	0.18
MAR	1950-1955	0	0.15	3.52	8.50	3.38	25.06	1.01	4.48	0.43	0.03
	2020-2025	0	0.00	1.94	5.40	3.64	26.28	1.54	7.25	0.85	0.08
	2095-2100	0	0.00	1.64	1.96	1.79	6.78	1.88	3.58	1.75	0.23
MCO	1950-1955	0	0.00	2.55	5.05	2.62	14.17	1.48	4.19	0.62	0.02
	2020-2025	0	0.00	1.98	2.51	1.98	10.40	1.86	4.85	1.57	0.12
	2095-2100	0	0.00	1.73	2.13	1.94	7.39	1.96	3.81	2.42	0.26
MDA	1950-1955	0	0.00	2.46	4.48	2.59	14.03	1.18	3.67	0.44	0.02
	2020-2025	0	0.00	1.56	2.35	1.87	8.65	1.39	3.51	0.63	0.03
	2095-2100	0	0.00	1.49	1.72	1.71	6.26	1.67	3.15	0.94	0.07
MDG	1950-1955	0	0.04	4.17	11.79	3.75	31.92	1.00	4.80	0.37	0.02
	2020-2025	0	0.08	3.28	9.84	4.20	36.21	1.41	6.98	0.80	0.07
	2095-2100	0	0.01	2.27	3.36	2.15	10.14	1.72	4.08	1.41	0.23

Table S3. Living kin for a female Focal aged 35-39. Country-level averages for selected years (values for 2095-2100 refer to the median of the 1,000 country-level projections). Relative codes: ggd = great-grandchildren, gd = grandchildren, d = children, n = niblings, s = siblings, c = cousins, m = parents, a = aunts/uncles, gm = grandparents, ggm = great-grandparents. ***(continued)***

ISO	Year	ggd	gd	d	n	s	c	m	a	gm	ggm
MDV	1950-1955	0	0.04	3.05	6.39	2.94	18.50	1.28	4.43	0.69	0.06
	2020-2025	0	0.00	1.95	6.26	4.30	27.07	1.77	7.51	1.43	0.22
	2095-2100	0	0.00	1.40	1.58	1.65	6.15	1.94	3.62	1.96	0.35
MEX	1950-1955	0	0.05	4.03	11.33	3.98	34.31	1.14	5.55	0.55	0.04
	2020-2025	0	0.01	1.93	4.98	3.17	26.43	1.60	7.92	1.26	0.20
	2095-2100	0	0.00	1.52	1.62	1.63	5.38	1.85	3.09	2.17	0.54
MHL	1950-1955	0	0.05	5.38	20.55	5.18	58.87	1.09	7.02	0.45	0.03
	2020-2025	0	0.02	3.15	11.88	4.94	53.59	1.38	9.18	0.72	0.06
	2095-2100	0	0.00	1.76	2.07	1.77	6.99	1.54	3.15	0.87	0.07
MKD	1950-1955	0	0.00	3.24	7.50	3.61	26.08	1.44	5.81	0.66	0.03
	2020-2025	0	0.00	1.52	2.14	1.99	10.71	1.72	4.77	1.16	0.07
	2095-2100	0	0.00	1.19	1.22	1.51	4.82	1.88	3.02	1.59	0.16
MLI	1950-1955	0	0.07	2.77	5.24	2.44	13.61	0.92	2.96	0.27	0.01
	2020-2025	0	0.09	4.22	11.26	4.24	26.52	1.33	4.75	0.61	0.04
	2095-2100	0	0.00	3.00	5.25	2.48	14.79	1.58	4.65	0.98	0.09
MLT	1950-1955	0	0.00	3.18	7.82	3.61	26.19	1.40	5.62	0.45	0.01
	2020-2025	0	0.00	1.29	1.70	1.95	11.30	1.81	5.40	1.43	0.12
	2095-2100	0	0.00	1.07	1.07	1.48	4.62	1.91	2.96	1.54	0.11
MMR	1950-1955	0	0.01	2.97	6.20	2.93	18.88	0.95	3.61	0.27	0.01
	2020-2025	0	0.00	1.75	3.95	2.86	18.52	1.32	5.32	0.50	0.02
	2095-2100	0	0.00	1.69	1.98	1.70	5.68	1.69	2.85	1.10	0.08
MNE	1950-1955	0	0.00	3.28	7.42	3.70	27.41	1.39	5.92	0.65	0.03
	2020-2025	0	0.00	1.70	2.29	2.00	11.11	1.67	4.85	1.14	0.09
	2095-2100	0	0.00	1.36	1.46	1.61	5.76	1.84	3.37	1.40	0.11
MNG	1950-1955	0	0.00	3.09	7.10	3.19	21.67	0.85	3.72	0.13	0.00
	2020-2025	0	0.00	2.20	6.05	3.31	25.36	1.31	6.70	0.57	0.02
	2095-2100	0	0.00	1.77	2.42	1.92	7.98	1.79	3.81	1.74	0.34
MNP	1950-1955	0	0.05	4.50	15.53	4.74	47.17	1.19	6.65	0.45	0.03
	2020-2025	0	0.01	2.50	5.54	3.15	27.80	1.73	8.66	1.26	0.13
	2095-2100	0	0.00	1.66	1.97	1.84	7.22	1.89	3.78	2.01	0.29
MOZ	1950-1955	0	0.07	2.93	6.23	2.88	17.96	1.06	3.76	0.39	0.02
	2020-2025	0	0.09	3.49	8.27	3.48	24.13	1.08	4.49	0.47	0.03
	2095-2100	0	0.01	2.33	3.46	2.12	10.51	1.61	3.97	1.10	0.15
MRT	1950-1955	0	0.05	3.11	6.82	3.12	21.03	1.04	4.08	0.40	0.02
	2020-2025	0	0.06	3.37	9.98	4.54	34.00	1.43	6.57	0.68	0.05
	2095-2100	0	0.01	2.50	4.10	2.40	13.03	1.69	4.60	1.14	0.12
MSR	1950-1955	0	0.07	3.27	7.79	3.10	21.15	1.14	4.35	0.58	0.06
	2020-2025	0	0.01	1.16	1.78	1.99	11.24	1.74	5.72	1.63	0.31
	2095-2100	0	0.00	1.39	1.51	1.67	4.64	1.87	2.73	1.97	0.35

Table S3. Living kin for a female Focal aged 35-39. Country-level averages for selected years (values for 2095-2100 refer to the median of the 1,000 country-level projections). Relative codes: ggd = great-grandchildren, gd = grandchildren, d = children, n = niblings, s = siblings, c = cousins, m = parents, a = aunts/uncles, gm = grandparents, ggm = great-grandparents. ***(continued)***

ISO	Year	ggd	gd	d	n	s	c	m	a	gm	ggm
MTQ	1950-1955	0	0.00	4.10	12.54	4.39	40.56	1.00	5.60	0.24	0.00
	2020-2025	0	0.00	1.69	2.45	2.28	19.01	1.75	7.53	1.54	0.18
	2095-2100	0	0.00	1.48	1.64	1.78	6.48	1.90	3.55	2.00	0.23
MUS	1950-1955	0	0.03	4.06	12.32	4.24	37.63	0.92	5.30	0.16	0.00
	2020-2025	0	0.00	1.57	2.41	2.17	17.34	1.59	6.73	1.09	0.12
	2095-2100	0	0.00	1.18	1.12	1.47	4.61	1.80	2.86	1.42	0.14
MWI	1950-1955	0	0.05	2.91	5.68	2.77	16.67	1.09	3.76	0.45	0.03
	2020-2025	0	0.06	3.51	9.06	3.64	24.36	1.00	4.33	0.50	0.05
	2095-2100	0	0.00	2.22	3.22	2.00	8.91	1.60	3.65	1.27	0.20
MYS	1950-1955	0	0.02	4.26	12.94	4.29	39.52	0.99	5.53	0.25	0.01
	2020-2025	0	0.00	1.83	4.41	3.49	29.89	1.60	8.02	1.05	0.09
	2095-2100	0	0.00	1.40	1.54	1.64	5.78	1.83	3.27	1.45	0.10
MYT	1950-1955	0	0.07	3.95	11.90	4.12	36.12	1.14	5.64	0.46	0.03
	2020-2025	0	0.03	3.76	13.69	5.35	51.84	1.67	9.46	1.17	0.14
	2095-2100	0	0.00	2.88	5.43	2.82	17.36	1.87	5.88	2.08	0.40
NAM	1950-1955	0	0.02	3.07	6.74	3.11	20.77	1.10	4.17	0.42	0.02
	2020-2025	0	0.02	2.63	7.43	3.57	26.16	0.94	4.95	0.48	0.05
	2095-2100	0	0.00	2.04	2.88	2.03	8.98	1.47	3.42	0.77	0.07
NCL	1950-1955	0	0.01	3.37	8.52	3.38	24.76	1.10	4.53	0.41	0.02
	2020-2025	0	0.00	1.88	4.12	3.05	21.16	1.68	6.57	1.37	0.19
	2095-2100	0	0.00	1.51	1.79	1.77	6.42	1.87	3.49	1.95	0.38
NER	1950-1955	0	0.13	3.67	9.09	3.45	26.25	1.01	4.55	0.39	0.02
	2020-2025	0	0.11	4.70	12.68	4.55	35.82	1.39	6.03	0.74	0.06
	2095-2100	0	0.00	3.71	7.85	3.01	22.16	1.69	6.00	1.27	0.16
NGA	1950-1955	0	0.05	3.03	6.26	2.92	18.66	1.00	3.75	0.35	0.02
	2020-2025	0	0.04	3.64	9.21	3.75	24.86	1.19	4.57	0.54	0.04
	2095-2100	0	0.00	2.58	4.12	2.17	10.74	1.48	3.76	0.83	0.06
NIC	1950-1955	0	0.06	4.05	11.22	3.84	32.55	1.05	5.19	0.45	0.03
	2020-2025	0	0.03	2.14	5.95	3.46	27.47	1.51	7.33	1.07	0.11
	2095-2100	0	0.00	1.74	2.11	1.79	6.52	1.86	3.39	2.12	0.55
NIU	1950-1955	0	0.01	4.56	14.53	4.72	46.13	1.34	7.37	0.73	0.05
	2020-2025	0	0.00	2.20	5.82	3.94	36.33	1.52	8.74	0.88	0.07
	2095-2100	0	0.00	1.71	2.01	1.82	7.11	1.73	3.42	1.12	0.06
NLD	1950-1955	0	0.00	2.45	4.66	2.88	16.56	1.60	4.87	0.70	0.02
	2020-2025	0	0.00	1.54	1.70	1.87	9.04	1.82	4.53	1.37	0.07
	2095-2100	0	0.00	1.39	1.44	1.64	5.53	1.93	3.28	1.83	0.06
NOR	1950-1955	0	0.00	2.11	3.61	2.33	10.98	1.62	3.94	0.88	0.04
	2020-2025	0	0.00	1.66	2.06	2.02	8.92	1.83	4.12	1.61	0.15
	2095-2100	0	0.00	1.35	1.37	1.60	5.43	1.94	3.30	1.93	0.10

Table S3. Living kin for a female Focal aged 35-39. Country-level averages for selected years (values for 2095-2100 refer to the median of the 1,000 country-level projections). Relative codes: ggd = great-grandchildren, gd = grandchildren, d = children, n = niblings, s = siblings, c = cousins, m = parents, a = aunts/uncles, gm = grandparents, ggm = great-grandparents. ***(continued)***

ISO	Year	ggd	gd	d	n	s	c	m	a	gm	ggm
NPL	1950-1955	0	0.03	3.10	6.71	2.97	19.30	1.07	4.00	0.44	0.03
	2020-2025	0	0.02	2.20	5.95	3.38	20.98	1.45	5.46	0.76	0.06
	2095-2100	0	0.00	1.78	2.15	1.65	5.69	1.87	3.24	2.24	0.59
NRU	1950-1955	0	0.01	3.47	8.63	3.81	30.25	1.15	5.27	0.32	0.01
	2020-2025	0	0.01	3.00	6.71	2.90	23.51	1.10	5.09	0.33	0.01
	2095-2100	0	0.00	2.27	3.47	2.14	10.09	1.50	3.67	0.98	0.11
NZL	1950-1955	0	0.00	3.09	7.42	3.26	21.68	1.55	5.42	0.84	0.05
	2020-2025	0	0.00	1.76	2.55	2.29	13.64	1.82	5.64	1.84	0.25
	2095-2100	0	0.00	1.44	1.57	1.63	5.87	1.91	3.48	1.97	0.24
OMN	1950-1955	0	0.19	2.74	5.14	2.52	14.17	1.08	3.42	0.58	0.08
	2020-2025	0	0.00	2.41	8.68	5.04	31.23	1.69	7.27	1.18	0.19
	2095-2100	0	0.00	1.63	2.17	1.89	8.57	1.92	4.35	1.61	0.17
PAK	1950-1955	0	0.06	3.32	7.93	3.42	24.54	1.17	4.83	0.52	0.04
	2020-2025	0	0.01	3.30	10.77	4.59	36.62	1.48	7.21	0.79	0.06
	2095-2100	0	0.00	2.25	3.42	2.10	9.22	1.69	3.82	1.15	0.09
PAN	1950-1955	0	0.03	3.93	11.30	4.07	34.37	1.36	6.35	0.85	0.10
	2020-2025	0	0.02	2.15	4.71	2.87	22.47	1.70	7.33	1.72	0.39
	2095-2100	0	0.00	1.74	2.14	1.82	6.88	1.88	3.58	2.34	0.74
PER	1950-1955	0	0.04	3.86	10.78	4.10	34.67	1.36	6.35	0.69	0.05
	2020-2025	0	0.01	2.00	5.12	3.29	26.35	1.63	7.64	1.18	0.12
	2095-2100	0	0.00	1.63	1.88	1.73	6.19	1.89	3.39	2.07	0.42
PHL	1950-1955	0	0.01	4.81	16.83	5.33	58.58	1.22	7.69	0.46	0.02
	2020-2025	0	0.00	2.55	7.11	3.86	38.73	1.58	8.94	0.82	0.04
	2095-2100	0	0.00	1.91	2.49	1.90	7.77	1.79	3.67	1.52	0.19
PLW	1950-1955	0	0.01	4.56	15.42	4.56	45.59	1.00	5.80	0.34	0.02
	2020-2025	0	0.00	1.66	3.32	2.55	21.93	1.29	6.48	0.58	0.04
	2095-2100	0	0.00	1.80	2.22	1.82	6.34	1.54	2.82	0.83	0.05
PNG	1950-1955	0	0.02	3.16	6.86	2.95	19.79	0.93	3.61	0.32	0.01
	2020-2025	0	0.02	2.78	7.89	4.03	29.11	1.33	6.05	0.54	0.03
	2095-2100	0	0.00	2.01	2.74	1.99	8.58	1.57	3.50	0.84	0.07
POL	1950-1955	0	0.00	2.76	5.55	2.91	17.42	1.39	4.57	0.63	0.03
	2020-2025	0	0.00	1.29	1.80	1.87	8.65	1.61	4.03	1.16	0.11
	2095-2100	0	0.00	1.29	1.34	1.56	4.87	1.82	2.90	1.51	0.15
PRI	1950-1955	0	0.01	3.72	10.40	3.95	32.26	1.40	6.26	0.90	0.11
	2020-2025	0	0.01	1.43	2.33	2.13	13.61	1.65	5.61	1.64	0.42
	2095-2100	0	0.00	1.27	1.27	1.54	4.36	1.83	2.72	2.25	0.74
PRK	1950-1955	0	0.00	1.43	1.07	0.77	2.06	0.46	0.66	0.13	0.00
	2020-2025	0	0.00	1.78	2.88	2.24	10.16	1.61	4.52	0.90	0.03
	2095-2100	0	0.00	1.47	1.62	1.68	5.64	1.82	3.13	1.67	0.12

Table S3. Living kin for a female Focal aged 35-39. Country-level averages for selected years (values for 2095-2100 refer to the median of the 1,000 country-level projections). Relative codes: ggd = great-grandchildren, gd = grandchildren, d = children, n = niblings, s = siblings, c = cousins, m = parents, a = aunts/uncles, gm = grandparents, ggm = great-grandparents. ***(continued)***

ISO	Year	ggd	gd	d	n	s	c	m	a	gm	ggm
PRT	1950-1955	0	0.00	2.19	3.67	2.48	12.47	1.41	3.89	0.61	0.03
	2020-2025	0	0.00	1.25	1.51	1.81	8.30	1.72	4.28	1.46	0.13
	2095-2100	0	0.00	1.05	1.00	1.46	4.62	1.87	2.97	1.42	0.07
PRY	1950-1955	0	0.03	4.38	14.38	4.97	49.83	1.27	7.44	0.53	0.03
	2020-2025	0	0.01	2.23	6.21	3.66	33.10	1.55	8.19	1.05	0.13
	2095-2100	0	0.00	1.78	2.18	1.82	6.89	1.80	3.42	1.71	0.34
PSE	1950-1955	0	0.04	4.72	14.82	4.67	47.34	1.08	6.36	0.44	0.02
	2020-2025	0	0.01	3.62	13.44	5.47	53.98	1.63	9.51	0.94	0.07
	2095-2100	0	0.00	2.19	3.40	2.12	9.89	1.91	4.47	2.25	0.50
PYF	1950-1955	0	0.02	3.92	10.97	4.05	34.32	1.29	6.10	0.65	0.05
	2020-2025	0	0.00	1.78	4.21	3.28	25.00	1.85	8.16	1.76	0.28
	2095-2100	0	0.00	1.40	1.48	1.63	5.50	1.93	3.32	2.22	0.50
QAT	1950-1955	0	0.19	4.22	12.92	4.35	39.02	1.38	6.85	0.81	0.09
	2020-2025	0	0.00	1.83	7.05	4.49	37.32	1.80	9.28	1.50	0.20
	2095-2100	0	0.00	1.44	1.78	1.69	6.06	1.95	3.49	1.83	0.16
ROU	1950-1955	0	0.00	2.39	4.28	2.55	13.10	1.48	4.18	0.79	0.04
	2020-2025	0	0.00	1.38	1.97	1.77	7.49	1.58	3.78	1.21	0.15
	2095-2100	0	0.00	1.44	1.60	1.68	5.90	1.83	3.29	1.63	0.20
RUS	1950-1955	0	0.00	2.24	3.54	2.30	11.21	1.12	3.23	0.41	0.01
	2020-2025	0	0.00	1.47	1.76	1.50	5.89	1.34	2.86	0.71	0.05
	2095-2100	0	0.00	1.43	1.59	1.67	5.90	1.71	3.07	1.28	0.17
RWA	1950-1955	0	0.03	4.12	12.00	4.56	42.28	1.20	6.60	0.53	0.03
	2020-2025	0	0.00	3.30	10.22	4.45	41.78	1.12	6.89	0.47	0.03
	2095-2100	0	0.00	2.25	3.29	2.20	11.01	1.64	4.19	0.99	0.08
SAU	1950-1955	0	0.28	3.75	10.17	3.81	30.72	1.18	5.43	0.61	0.07
	2020-2025	0	0.00	2.32	8.54	5.07	39.66	1.68	8.74	1.15	0.17
	2095-2100	0	0.00	1.66	2.07	1.86	7.72	1.91	3.93	1.86	0.21
SDN	1950-1955	0	0.14	3.94	10.88	4.09	34.72	1.28	6.15	0.71	0.07
	2020-2025	0	0.03	3.44	10.06	4.08	33.00	1.21	6.00	0.67	0.07
	2095-2100	0	0.00	2.55	4.23	2.39	12.72	1.59	4.30	0.92	0.08
SEN	1950-1955	0	0.11	3.34	7.77	3.22	22.43	1.12	4.44	0.46	0.03
	2020-2025	0	0.03	3.49	10.37	4.50	33.52	1.41	6.35	0.72	0.06
	2095-2100	0	0.00	2.62	4.65	2.63	15.31	1.83	5.34	1.64	0.22
SGP	1950-1955	0	0.00	4.59	16.30	4.82	48.75	1.02	6.21	0.18	0.00
	2020-2025	0	0.00	1.11	1.32	1.86	16.70	1.81	8.08	1.53	0.16
	2095-2100	0	0.00	0.95	0.73	1.24	3.37	1.90	2.51	1.59	0.07
SHN	1950-1955	0	0.00	4.61	15.81	4.84	47.83	1.53	8.05	0.89	0.06
	2020-2025	0	0.00	1.45	2.63	2.45	18.72	1.74	7.45	1.36	0.14
	2095-2100	0	0.00	1.37	1.44	1.58	5.12	1.87	3.05	1.47	0.14

Table S3. Living kin for a female Focal aged 35-39. Country-level averages for selected years (values for 2095-2100 refer to the median of the 1,000 country-level projections). Relative codes: ggd = great-grandchildren, gd = grandchildren, d = children, n = niblings, s = siblings, c = cousins, m = parents, a = aunts/uncles, gm = grandparents, ggm = great-grandparents. ***(continued)***

ISO	Year	ggd	gd	d	n	s	c	m	a	gm	ggm
SLB	1950-1955	0	0.02	3.87	10.31	3.82	32.39	0.89	4.55	0.23	0.01
	2020-2025	0	0.02	3.46	11.14	4.59	41.85	1.47	7.91	0.68	0.03
	2095-2100	0	0.00	2.50	4.19	2.43	13.08	1.75	4.71	1.35	0.15
SLE	1950-1955	0	0.15	2.36	3.86	2.09	9.96	1.07	2.82	0.59	0.08
	2020-2025	0	0.07	3.28	7.95	3.73	20.46	1.23	4.34	0.64	0.08
	2095-2100	0	0.00	2.11	2.88	1.92	8.32	1.59	3.48	0.97	0.09
SLV	1950-1955	0	0.04	3.82	9.92	3.52	27.93	1.06	4.76	0.49	0.04
	2020-2025	0	0.02	1.92	4.46	2.91	22.26	1.41	6.28	1.24	0.24
	2095-2100	0	0.00	1.49	1.56	1.59	5.09	1.78	2.89	1.87	0.47
SMR	1950-1955	0	0.00	2.57	5.07	2.75	15.43	1.45	4.39	0.71	0.04
	2020-2025	0	0.00	0.99	1.12	1.72	7.84	1.87	4.42	1.75	0.20
	2095-2100	0	0.00	1.03	0.91	1.36	3.58	1.94	2.54	1.71	0.07
SOM	1950-1955	0	0.04	3.76	10.25	3.90	32.64	1.03	5.00	0.31	0.01
	2020-2025	0	0.08	4.36	11.60	4.08	36.27	1.17	5.79	0.45	0.02
	2095-2100	0	0.00	3.19	5.93	2.50	14.59	1.55	4.46	0.95	0.09
SPM	1950-1955	0	0.00	3.18	7.31	3.08	21.16	0.95	3.77	0.27	0.01
	2020-2025	0	0.00	1.56	2.39	2.12	13.98	1.66	5.65	0.98	0.07
	2095-2100	0	0.00	1.34	1.37	1.65	5.09	1.87	3.00	1.91	0.27
SRB	1950-1955	0	0.00	2.15	3.44	2.26	10.42	1.50	3.75	0.86	0.06
	2020-2025	0	0.00	1.38	1.54	1.61	5.55	1.63	2.96	1.16	0.11
	2095-2100	0	0.00	1.25	1.30	1.57	5.17	1.84	3.06	1.36	0.11
SSD	1950-1955	0	0.05	2.77	5.30	2.55	14.73	0.87	3.04	0.28	0.01
	2020-2025	0	0.07	3.38	6.27	2.91	16.05	0.77	2.80	0.31	0.02
	2095-2100	0	0.00	2.19	3.04	1.96	8.77	1.51	3.44	0.91	0.07
STP	1950-1955	0	0.03	3.44	9.19	3.20	22.94	1.15	4.36	0.43	0.01
	2020-2025	0	0.03	3.53	10.28	4.44	36.75	1.41	6.89	0.71	0.04
	2095-2100	0	0.00	2.27	3.44	2.20	11.10	1.60	4.11	1.11	0.13
SUR	1950-1955	0	0.02	4.73	15.61	4.74	47.88	1.03	6.39	0.25	0.01
	2020-2025	0	0.02	2.30	4.73	2.92	26.74	1.48	7.55	1.02	0.12
	2095-2100	0	0.00	1.66	1.97	1.82	7.03	1.75	3.45	1.48	0.23
SVK	1950-1955	0	0.00	2.77	5.85	2.93	17.52	1.52	4.86	0.88	0.05
	2020-2025	0	0.00	1.28	1.82	1.92	8.93	1.67	4.18	1.24	0.13
	2095-2100	0	0.00	1.35	1.55	1.68	5.07	1.85	2.92	1.65	0.22
SVN	1950-1955	0	0.00	2.08	3.07	2.23	10.22	1.31	3.39	0.52	0.02
	2020-2025	0	0.00	1.41	1.61	1.66	6.05	1.75	3.32	1.45	0.15
	2095-2100	0	0.00	1.35	1.51	1.67	5.63	1.90	3.26	1.78	0.19
SWE	1950-1955	0	0.00	1.87	2.83	2.00	8.10	1.62	3.38	0.91	0.05
	2020-2025	0	0.00	1.62	2.02	1.97	7.46	1.82	3.56	1.62	0.17
	2095-2100	0	0.00	1.43	1.57	1.67	5.79	1.94	3.37	2.01	0.13

Table S3. Living kin for a female Focal aged 35-39. Country-level averages for selected years (values for 2095-2100 refer to the median of the 1,000 country-level projections). Relative codes: ggd = great-grandchildren, gd = grandchildren, d = children, n = niblings, s = siblings, c = cousins, m = parents, a = aunts/uncles, gm = grandparents, ggm = great-grandparents. ***(continued)***

ISO	Year	ggd	gd	d	n	s	c	m	a	gm	ggm
SWZ	1950-1955	0	0.10	3.65	9.59	3.65	28.76	1.05	4.87	0.45	0.03
	2020-2025	0	0.02	2.38	6.92	3.38	25.80	0.80	4.52	0.49	0.08
	2095-2100	0	0.00	1.90	2.38	1.80	6.74	1.46	2.84	0.88	0.10
SXM	1950-1955	0	0.00	3.72	10.18	4.13	34.58	1.43	6.58	0.61	0.02
	2020-2025	0	0.00	1.46	2.45	2.13	14.23	1.69	6.01	1.01	0.07
	2095-2100	0	0.00	1.33	1.32	1.58	5.08	1.87	3.08	1.79	0.11
SYC	1950-1955	0	0.01	3.47	9.06	3.53	26.82	0.87	4.16	0.19	0.00
	2020-2025	0	0.01	1.92	3.95	2.65	19.56	1.53	6.51	0.85	0.05
	2095-2100	0	0.00	1.77	2.16	1.85	7.04	1.86	3.57	1.94	0.35
SYR	1950-1955	0	0.03	4.14	12.48	4.47	41.70	1.16	6.29	0.45	0.02
	2020-2025	0	0.01	2.66	9.22	4.66	43.41	1.55	8.90	0.85	0.06
	2095-2100	0	0.00	1.85	2.29	1.88	7.40	1.80	3.56	1.54	0.13
TCA	1950-1955	0	0.03	3.64	9.14	3.65	28.18	1.21	5.27	0.54	0.04
	2020-2025	0	0.00	1.50	3.55	3.37	23.21	1.70	7.41	1.20	0.14
	2095-2100	0	0.00	1.31	1.38	1.58	5.23	1.85	3.03	1.25	0.07
TCD	1950-1955	0	0.08	3.02	6.22	2.73	16.75	1.04	3.60	0.43	0.03
	2020-2025	0	0.09	4.30	11.16	4.11	28.16	1.18	4.62	0.62	0.06
	2095-2100	0	0.00	3.22	6.09	2.60	16.49	1.41	4.35	0.75	0.06
TGO	1950-1955	0	0.07	3.52	8.73	3.46	25.81	1.13	4.78	0.44	0.02
	2020-2025	0	0.03	3.36	9.84	4.14	32.92	1.30	6.32	0.60	0.04
	2095-2100	0	0.00	2.65	4.47	2.36	12.02	1.63	4.20	1.06	0.08
THA	1950-1955	0	0.01	3.77	9.75	3.71	30.62	0.88	4.44	0.29	0.02
	2020-2025	0	0.00	1.30	2.31	2.12	16.14	1.55	6.70	1.27	0.18
	2095-2100	0	0.00	1.18	1.14	1.44	4.22	1.83	2.71	1.81	0.36
TJK	1950-1955	0	0.00	3.19	7.24	3.62	26.36	1.31	5.57	0.59	0.03
	2020-2025	0	0.00	2.78	8.70	3.94	31.90	1.43	7.22	0.70	0.04
	2095-2100	0	0.00	2.26	3.66	2.05	8.69	1.84	3.89	2.12	0.47
TKL	1950-1955	0	0.05	3.93	11.66	4.10	35.63	1.09	5.48	0.39	0.02
	2020-2025	0	0.02	2.67	5.90	3.85	31.63	1.65	7.49	1.09	0.11
	2095-2100	0	0.00	1.79	2.24	1.94	8.05	1.83	3.87	1.62	0.19
TKM	1950-1955	0	0.00	3.17	7.17	3.50	25.15	1.13	4.90	0.41	0.02
	2020-2025	0	0.00	2.44	6.64	3.47	27.12	1.45	7.10	0.67	0.03
	2095-2100	0	0.00	2.32	3.65	2.02	8.90	1.74	3.55	1.44	0.16
TLS	1950-1955	0	0.01	2.82	5.27	2.52	14.83	0.70	2.62	0.15	0.00
	2020-2025	0	0.01	3.34	6.41	3.33	19.40	1.15	3.77	0.37	0.02
	2095-2100	0	0.00	2.00	2.71	1.92	7.71	1.79	3.57	1.59	0.18
TON	1950-1955	0	0.01	4.60	14.77	4.95	51.39	1.17	7.03	0.40	0.01
	2020-2025	0	0.00	3.00	8.29	4.30	43.09	1.42	8.39	0.65	0.04
	2095-2100	0	0.00	2.35	3.63	2.25	10.85	1.71	4.15	1.26	0.11

Table S3. Living kin for a female Focal aged 35-39. Country-level averages for selected years (values for 2095-2100 refer to the median of the 1,000 country-level projections). Relative codes: ggd = great-grandchildren, gd = grandchildren, d = children, n = niblings, s = siblings, c = cousins, m = parents, a = aunts/uncles, gm = grandparents, ggm = great-grandparents. ***(continued)***

ISO	Year	ggd	gd	d	n	s	c	m	a	gm	ggm
TTO	1950-1955	0	0.03	3.94	11.81	3.98	33.50	1.21	5.74	0.48	0.03
	2020-2025	0	0.00	1.43	2.57	2.28	16.32	1.58	6.32	1.26	0.18
	2095-2100	0	0.00	1.41	1.49	1.62	4.76	1.82	2.77	1.82	0.35
TUN	1950-1955	0	0.03	3.48	8.42	3.35	24.90	0.96	4.17	0.30	0.01
	2020-2025	0	0.00	1.71	4.40	3.43	25.14	1.67	7.64	1.01	0.08
	2095-2100	0	0.00	1.57	1.91	1.79	6.44	1.87	3.43	1.64	0.14
TUV	1950-1955	0	0.01	3.08	6.41	2.76	17.61	0.91	3.29	0.26	0.01
	2020-2025	0	0.00	2.72	5.34	3.19	20.40	1.28	4.48	0.43	0.01
	2095-2100	0	0.00	2.25	3.39	2.17	10.14	1.52	3.69	0.84	0.07
TWN	1950-1955	0	0.01	4.63	16.48	5.27	56.42	1.17	7.42	0.26	0.00
	2020-2025	0	0.00	1.17	1.59	2.11	17.12	1.73	7.25	1.51	0.16
	2095-2100	0	0.00	0.88	0.85	1.42	4.35	1.83	2.78	1.15	0.05
TZA	1950-1955	0	0.09	3.43	8.26	3.39	24.91	1.06	4.56	0.47	0.04
	2020-2025	0	0.04	3.71	10.74	4.14	32.25	1.21	5.74	0.63	0.07
	2095-2100	0	0.01	2.57	4.18	2.41	13.16	1.72	4.73	1.48	0.25
UGA	1950-1955	0	0.10	3.56	9.03	3.30	24.71	0.98	4.18	0.45	0.04
	2020-2025	0	0.06	4.19	11.77	4.36	35.90	1.09	5.46	0.49	0.04
	2095-2100	0	0.00	2.31	3.51	2.11	10.61	1.57	3.99	0.96	0.09
UKR	1950-1955	0	0.00	1.92	2.68	2.01	8.33	1.31	3.06	0.54	0.02
	2020-2025	0	0.00	1.30	1.59	1.51	5.24	1.43	2.74	0.85	0.07
	2095-2100	0	0.00	1.15	1.30	1.54	4.69	1.68	2.72	1.25	0.20
URY	1950-1955	0	0.01	2.11	3.46	2.27	10.43	1.46	3.65	0.81	0.07
	2020-2025	0	0.01	1.68	2.67	2.21	9.87	1.67	4.09	1.23	0.16
	2095-2100	0	0.00	1.27	1.29	1.53	4.89	1.85	2.99	1.77	0.33
USA	1950-1955	0	0.01	2.67	5.59	2.73	15.29	1.48	4.43	0.92	0.09
	2020-2025	0	0.00	1.80	2.11	1.85	9.69	1.73	4.49	1.73	0.32
	2095-2100	0	0.00	1.43	1.53	1.63	5.83	1.84	3.31	1.95	0.29
UZB	1950-1955	0	0.00	3.57	8.92	3.86	30.34	1.37	6.12	0.70	0.04
	2020-2025	0	0.00	2.22	6.30	3.36	28.36	1.51	7.71	0.74	0.04
	2095-2100	0	0.00	1.97	2.76	2.02	8.33	1.77	3.75	1.53	0.21
VCT	1950-1955	0	0.05	4.80	16.90	4.84	49.89	1.22	7.07	0.64	0.06
	2020-2025	0	0.01	1.70	3.49	2.77	24.71	1.62	8.31	1.57	0.32
	2095-2100	0	0.00	1.43	1.58	1.65	5.53	1.77	3.06	1.70	0.36
VEN	1950-1955	0	0.05	4.49	14.35	4.42	42.47	1.16	6.28	0.57	0.04
	2020-2025	0	0.03	2.01	4.83	2.95	25.62	1.58	7.65	1.24	0.12
	2095-2100	0	0.00	1.71	1.98	1.72	5.99	1.81	3.14	1.96	0.41
VGB	1950-1955	0	0.07	3.84	10.50	4.01	32.66	1.31	6.08	0.67	0.06
	2020-2025	0	0.00	1.04	1.75	2.00	13.37	1.73	6.32	1.30	0.17
	2095-2100	0	0.00	1.03	0.91	1.29	3.45	1.86	2.44	1.58	0.15

Table S3. Living kin for a female Focal aged 35-39. Country-level averages for selected years (values for 2095-2100 refer to the median of the 1,000 country-level projections). Relative codes: ggd = great-grandchildren, gd = grandchildren, d = children, n = niblings, s = siblings, c = cousins, m = parents, a = aunts/uncles, gm = grandparents, ggm = great-grandparents. ***(continued)***

ISO	Year	ggd	gd	d	n	s	c	m	a	gm	ggm
VIR	1950-1955	0	0.03	3.46	8.91	3.47	25.52	1.28	5.15	0.67	0.06
	2020-2025	0	0.01	2.04	4.21	2.80	19.57	1.61	6.20	1.12	0.11
	2095-2100	0	0.00	1.74	2.11	1.83	6.80	1.85	3.50	1.95	0.34
VNM	1950-1955	0	0.00	3.42	8.15	3.27	24.54	1.00	4.03	0.28	0.01
	2020-2025	0	0.00	1.71	4.01	2.78	21.56	1.57	7.02	0.74	0.03
	2095-2100	0	0.00	1.56	1.87	1.76	6.73	1.81	3.52	1.61	0.20
VUT	1950-1955	0	0.01	4.53	13.96	4.33	41.66	1.01	5.64	0.38	0.02
	2020-2025	0	0.02	3.20	10.16	4.42	40.83	1.52	8.18	0.80	0.05
	2095-2100	0	0.00	2.52	4.29	2.48	13.19	1.79	4.78	1.59	0.25
WSM	1950-1955	0	0.04	4.97	17.17	5.05	54.42	1.12	6.98	0.43	0.02
	2020-2025	0	0.00	3.43	10.79	4.79	51.60	1.57	9.85	0.89	0.06
	2095-2100	0	0.00	2.60	4.37	2.43	12.71	1.83	4.79	1.82	0.30
YEM	1950-1955	0	0.03	2.96	6.39	3.15	21.03	0.99	4.00	0.31	0.01
	2020-2025	0	0.03	3.50	11.94	5.24	37.24	1.43	6.52	0.64	0.04
	2095-2100	0	0.00	2.14	3.04	2.06	9.74	1.65	3.92	0.99	0.08
ZAF	1950-1955	0	0.03	3.54	9.31	3.65	28.47	1.09	4.89	0.45	0.03
	2020-2025	0	0.01	1.82	4.55	2.83	20.89	1.11	5.16	0.65	0.08
	2095-2100	0	0.00	1.79	2.14	1.70	5.87	1.49	2.63	1.07	0.20
ZMB	1950-1955	0	0.08	3.64	9.56	3.78	29.94	1.23	5.53	0.65	0.07
	2020-2025	0	0.05	3.61	10.25	4.13	35.63	1.07	5.96	0.58	0.06
	2095-2100	0	0.01	2.47	3.87	2.29	11.75	1.75	4.48	1.47	0.24
ZWE	1950-1955	0	0.15	4.15	12.86	4.43	40.95	1.18	6.30	0.58	0.06
	2020-2025	0	0.02	2.78	7.66	3.41	31.02	0.88	5.52	0.50	0.07
	2095-2100	0	0.00	2.12	2.95	1.99	8.73	1.46	3.35	0.90	0.10

Table S4. Living kin for a female Focal aged 65-69. Country-level averages for selected years (values for 2095-2100 refer to the median of the 1,000 country-level projections). Relative codes: ggd = great-grandchildren, gd = grandchildren, d = children, n = niblings, s = siblings, c = cousins, m = parents, a = aunts/uncles, gm = grandparents, ggm = great-grandparents.

ISO	Year	ggd	gd	d	n	s	c	m	a	gm	ggm
ABW	1950-1955	2.38	17.35	4.29	19.56	2.68	27.94	0.05	1.15	0.00	0
	2020-2025	0.30	3.49	2.09	8.24	2.92	24.12	0.18	2.28	0.00	0
	2095-2100	0.00	1.09	1.29	2.25	1.49	5.59	0.51	1.50	0.01	0
AFG	1950-1955	1.39	8.90	2.63	8.10	1.08	8.71	0.02	0.36	0.00	0
	2020-2025	3.33	18.53	4.69	16.20	2.12	16.91	0.07	0.89	0.00	0
	2095-2100	0.43	6.01	2.67	5.48	1.73	17.09	0.22	2.38	0.00	0
AGO	1950-1955	1.35	7.76	2.63	7.73	1.46	10.12	0.05	0.72	0.00	0
	2020-2025	3.47	15.39	4.04	13.69	1.98	14.73	0.08	0.88	0.00	0
	2095-2100	0.61	8.03	3.20	7.28	1.90	17.87	0.19	2.10	0.00	0
AIA	1950-1955	6.64	23.24	4.84	25.15	3.16	37.19	0.08	1.69	0.00	0
	2020-2025	0.48	4.22	2.93	13.48	4.18	39.97	0.25	3.19	0.00	0
	2095-2100	0.03	1.46	1.37	2.04	1.31	4.82	0.63	1.42	0.03	0
ALB	1950-1955	0.66	10.83	3.72	14.17	2.52	21.64	0.06	1.23	0.00	0
	2020-2025	0.40	5.38	3.16	11.22	3.34	25.46	0.23	2.44	0.00	0
	2095-2100	0.02	1.79	1.62	2.68	1.55	6.60	0.73	2.03	0.03	0
AND	1950-1955	0.07	3.36	2.01	4.13	1.47	6.53	0.09	0.67	0.00	0
	2020-2025	0.02	1.96	1.76	3.72	2.00	8.28	0.35	1.45	0.00	0
	2095-2100	0.00	1.03	1.27	1.86	1.30	4.12	0.65	1.39	0.01	0
ARE	1950-1955	3.60	13.42	3.44	13.34	2.04	18.57	0.07	1.16	0.00	0
	2020-2025	0.53	8.42	5.03	18.48	4.03	34.05	0.30	2.91	0.01	0
	2095-2100	0.00	1.33	1.46	2.42	1.54	10.61	0.53	2.05	0.01	0
ARG	1950-1955	0.73	5.98	2.53	6.68	1.71	10.02	0.06	0.76	0.00	0
	2020-2025	0.62	5.33	2.73	6.86	2.00	11.36	0.22	1.36	0.00	0
	2095-2100	0.04	2.17	1.77	3.14	1.67	7.91	0.63	2.13	0.03	0
ARM	1950-1955	1.14	12.09	3.74	14.56	2.69	23.54	0.11	1.67	0.00	0
	2020-2025	0.65	3.45	2.08	6.98	2.42	17.76	0.13	1.89	0.00	0
	2095-2100	0.02	1.84	1.63	2.76	1.54	5.58	0.52	1.51	0.02	0
ASM	1950-1955	3.81	22.20	4.89	25.21	3.36	39.02	0.11	1.98	0.00	0
	2020-2025	1.05	11.21	3.96	21.56	4.15	42.80	0.13	2.47	0.00	0
	2095-2100	0.05	2.50	1.94	3.76	1.76	12.41	0.34	2.13	0.00	0
ATG	1950-1955	2.94	12.67	3.59	13.76	2.32	20.35	0.09	1.28	0.00	0
	2020-2025	0.44	3.52	2.17	7.48	2.74	19.63	0.32	2.56	0.01	0
	2095-2100	0.04	1.95	1.59	2.52	1.49	5.48	0.77	1.82	0.05	0
AUS	1950-1955	1.00	7.58	2.81	8.15	2.01	12.61	0.11	0.97	0.00	0
	2020-2025	0.12	3.58	2.13	5.94	2.47	13.78	0.42	2.15	0.00	0
	2095-2100	0.01	1.81	1.61	2.80	1.63	6.60	0.71	2.12	0.01	0
AUT	1950-1955	0.26	2.92	1.75	3.17	1.27	4.98	0.11	0.66	0.00	0
	2020-2025	0.10	2.24	1.58	3.21	1.71	6.11	0.35	1.25	0.00	0
	2095-2100	0.00	1.42	1.51	2.52	1.57	4.99	0.64	1.60	0.01	0

Table S4. Living kin for a female Focal aged 65-69. Country-level averages for selected years (values for 2095-2100 refer to the median of the 1,000 country-level projections). Relative codes: ggd = great-grandchildren, gd = grandchildren, d = children, n = niblings, s = siblings, c = cousins, m = parents, a = aunts/uncles, gm = grandparents, ggm = great-grandparents. ***(continued)***

ISO	Year	ggd	gd	d	n	s	c	m	a	gm	ggm
AZE	1950-1955	0.92	11.34	3.54	13.36	2.25	19.87	0.06	1.18	0.00	0
	2020-2025	0.77	4.68	2.65	9.38	2.87	21.39	0.11	1.72	0.00	0
	2095-2100	0.07	2.73	1.79	2.83	1.40	5.34	0.57	1.55	0.02	0
BDI	1950-1955	1.31	11.90	3.63	14.00	2.07	18.76	0.05	0.95	0.00	0
	2020-2025	1.47	15.77	4.41	16.43	2.21	20.27	0.06	1.05	0.00	0
	2095-2100	0.26	6.70	2.99	6.54	1.84	18.29	0.19	2.24	0.00	0
BEL	1950-1955	0.26	3.85	2.05	4.33	1.49	6.83	0.11	0.76	0.00	0
	2020-2025	0.08	2.78	1.68	3.56	1.72	7.21	0.33	1.37	0.00	0
	2095-2100	0.01	1.90	1.62	2.81	1.62	5.92	0.72	1.94	0.01	0
BEN	1950-1955	1.11	7.34	2.64	7.60	1.44	9.81	0.05	0.66	0.00	0
	2020-2025	2.71	15.73	4.45	14.24	2.26	16.23	0.11	1.12	0.00	0
	2095-2100	0.56	7.93	3.06	7.11	1.90	17.33	0.18	1.97	0.00	0
BES	1950-1955	2.60	18.17	4.54	21.33	3.29	33.48	0.12	1.85	0.00	0
	2020-2025	0.31	3.74	2.35	9.31	3.20	27.46	0.21	2.59	0.00	0
	2095-2100	0.02	1.98	1.62	2.63	1.51	5.92	0.56	1.52	0.02	0
BFA	1950-1955	1.25	6.93	2.47	6.79	1.32	8.70	0.03	0.56	0.00	0
	2020-2025	2.82	15.65	4.36	13.84	2.10	14.95	0.07	0.94	0.00	0
	2095-2100	0.49	6.01	2.67	5.47	1.64	15.16	0.15	1.85	0.00	0
BGD	1950-1955	3.54	10.51	3.19	10.91	2.00	15.83	0.06	1.03	0.00	0
	2020-2025	2.33	8.24	3.79	13.09	3.09	22.94	0.21	1.95	0.00	0
	2095-2100	0.19	2.73	1.66	2.90	1.57	7.41	0.81	2.30	0.06	0
BGR	1950-1955	0.63	3.56	1.84	3.57	1.35	5.71	0.14	0.78	0.00	0
	2020-2025	0.35	2.41	1.69	2.89	1.24	5.25	0.21	0.93	0.00	0
	2095-2100	0.02	1.80	1.68	2.75	1.50	4.80	0.55	1.42	0.02	0
BHR	1950-1955	3.24	12.06	3.35	12.38	2.03	17.35	0.08	1.13	0.00	0
	2020-2025	0.62	7.81	4.17	17.32	4.19	32.46	0.33	2.87	0.01	0
	2095-2100	0.03	2.18	1.71	3.02	1.69	10.19	0.71	2.37	0.02	0
BHS	1950-1955	2.41	12.78	3.52	13.55	2.20	19.20	0.09	1.14	0.00	0
	2020-2025	0.46	4.28	2.52	9.08	2.64	20.00	0.24	1.99	0.01	0
	2095-2100	0.03	1.57	1.40	2.09	1.25	5.03	0.52	1.41	0.02	0
BIH	1950-1955	0.85	9.99	3.37	11.87	2.26	18.30	0.06	1.17	0.00	0
	2020-2025	0.19	2.46	1.81	5.02	2.00	13.44	0.19	1.83	0.00	0
	2095-2100	0.01	1.41	1.43	2.29	1.45	4.49	0.61	1.42	0.02	0
BLR	1950-1955	0.27	4.21	2.08	4.59	1.40	7.01	0.07	0.67	0.00	0
	2020-2025	0.29	2.61	1.72	3.56	1.39	6.44	0.12	0.80	0.00	0
	2095-2100	0.01	1.89	1.57	2.88	1.55	4.99	0.47	1.32	0.02	0
BLZ	1950-1955	6.00	19.74	4.30	20.32	2.65	28.86	0.08	1.36	0.00	0
	2020-2025	2.54	11.71	4.52	19.42	3.56	36.10	0.26	2.69	0.00	0
	2095-2100	0.13	2.56	1.69	2.98	1.55	9.16	0.63	2.38	0.03	0

Table S4. Living kin for a female Focal aged 65-69. Country-level averages for selected years (values for 2095-2100 refer to the median of the 1,000 country-level projections). Relative codes: ggd = great-grandchildren, gd = grandchildren, d = children, n = nibling, s = siblings, c = cousins, m = parents, a = aunts/uncles, gm = grandparents, ggm = great-grandparents. ***(continued)***

ISO	Year	ggd	gd	d	n	s	c	m	a	gm	ggm
BMU	1950-1955	1.18	7.02	2.42	6.69	1.39	8.85	0.05	0.58	0.00	0
	2020-2025	0.08	2.52	1.70	4.71	2.23	11.44	0.38	1.95	0.01	0
	2095-2100	0.01	1.23	1.42	2.23	1.42	5.15	0.60	1.64	0.01	0
BOL	1950-1955	1.27	10.09	3.12	10.69	1.86	14.68	0.06	0.93	0.00	0
	2020-2025	1.15	8.59	3.57	12.61	2.56	19.45	0.09	1.28	0.00	0
	2095-2100	0.16	3.36	2.00	3.57	1.53	9.49	0.30	1.68	0.01	0
BRA	1950-1955	2.16	13.84	3.67	14.70	2.33	21.23	0.09	1.30	0.00	0
	2020-2025	0.67	4.69	2.73	10.09	3.01	23.70	0.19	2.13	0.00	0
	2095-2100	0.04	1.90	1.62	2.62	1.51	5.97	0.63	1.74	0.03	0
BRB	1950-1955	2.13	9.29	3.08	9.97	2.06	14.96	0.11	1.15	0.00	0
	2020-2025	0.30	2.68	1.81	5.80	2.59	15.33	0.30	2.19	0.00	0
	2095-2100	0.01	1.88	1.59	2.74	1.58	5.14	0.68	1.59	0.03	0
BRN	1950-1955	4.07	20.88	4.66	23.05	3.08	34.21	0.08	1.60	0.00	0
	2020-2025	0.38	6.78	3.82	17.28	4.01	39.73	0.20	2.68	0.00	0
	2095-2100	0.01	1.96	1.66	2.84	1.55	8.04	0.44	1.62	0.01	0
BTN	1950-1955	1.24	8.44	2.65	8.06	1.14	9.02	0.02	0.41	0.00	0
	2020-2025	0.88	8.25	4.23	12.53	2.52	18.25	0.11	1.25	0.00	0
	2095-2100	0.02	1.67	1.41	2.31	1.41	7.73	0.53	1.96	0.01	0
BWA	1950-1955	2.59	12.71	3.66	14.35	2.07	19.65	0.06	1.12	0.00	0
	2020-2025	1.48	9.81	3.89	14.97	2.46	23.27	0.10	1.31	0.00	0
	2095-2100	0.10	3.48	2.06	3.68	1.40	8.48	0.18	1.19	0.00	0
CAF	1950-1955	2.07	7.77	2.45	7.03	1.22	8.54	0.04	0.57	0.00	0
	2020-2025	3.92	13.80	3.56	11.95	1.68	12.55	0.09	0.81	0.00	0
	2095-2100	0.70	7.59	3.05	6.06	1.53	14.18	0.15	1.66	0.00	0
CAN	1950-1955	1.23	9.15	3.15	10.21	2.33	16.24	0.16	1.35	0.00	0
	2020-2025	0.08	2.59	1.74	5.34	2.54	15.04	0.41	2.36	0.01	0
	2095-2100	0.00	1.40	1.49	2.44	1.55	5.34	0.70	1.79	0.01	0
CHE	1950-1955	0.16	3.88	2.08	4.46	1.56	7.14	0.11	0.74	0.00	0
	2020-2025	0.03	2.22	1.66	3.45	1.79	7.55	0.37	1.50	0.00	0
	2095-2100	0.00	1.58	1.55	2.48	1.53	5.15	0.68	1.73	0.01	0
CHL	1950-1955	1.33	10.01	3.40	12.12	2.17	17.83	0.05	1.05	0.00	0
	2020-2025	0.40	3.87	2.39	7.74	2.69	18.52	0.33	2.45	0.01	0
	2095-2100	0.02	1.67	1.53	2.52	1.54	6.16	0.75	1.98	0.05	0
CHN	1950-1955	2.29	15.08	3.93	16.62	2.01	20.90	0.02	0.69	0.00	0
	2020-2025	0.42	3.96	2.33	9.72	3.38	26.70	0.20	2.32	0.00	0
	2095-2100	0.01	1.32	1.32	2.28	1.54	5.53	0.68	1.88	0.03	0
CIV	1950-1955	2.12	9.86	3.01	9.97	1.66	13.19	0.05	0.84	0.00	0
	2020-2025	3.44	16.22	4.58	17.70	2.46	20.88	0.10	1.21	0.00	0
	2095-2100	0.53	6.65	2.81	6.21	1.70	15.27	0.17	1.69	0.00	0

Table S4. Living kin for a female Focal aged 65-69. Country-level averages for selected years (values for 2095-2100 refer to the median of the 1,000 country-level projections). Relative codes: ggd = great-grandchildren, gd = grandchildren, d = children, n = niblings, s = siblings, c = cousins, m = parents, a = aunts/uncles, gm = grandparents, ggm = great-grandparents. ***(continued)***

ISO	Year	ggd	gd	d	n	s	c	m	a	gm	ggm
CMR	1950-1955	1.34	6.04	2.26	5.77	1.29	7.70	0.07	0.70	0.00	0
	2020-2025	3.57	15.15	4.18	12.86	2.00	13.57	0.13	1.04	0.00	0
	2095-2100	0.48	6.54	2.78	6.20	1.75	15.43	0.20	1.89	0.00	0
COD	1950-1955	2.06	8.65	2.86	8.94	1.64	12.24	0.06	0.90	0.00	0
	2020-2025	3.00	16.38	4.13	14.53	2.19	17.15	0.11	1.28	0.00	0
	2095-2100	0.50	10.82	3.73	9.00	2.08	21.56	0.16	2.21	0.00	0
COG	1950-1955	1.81	10.15	3.01	10.20	1.62	13.09	0.06	0.80	0.00	0
	2020-2025	2.62	13.37	4.07	15.18	2.34	19.19	0.09	1.10	0.00	0
	2095-2100	0.42	6.19	2.75	6.14	1.76	14.46	0.18	1.71	0.00	0
COK	1950-1955	4.09	16.66	3.52	14.99	1.65	17.67	0.03	0.71	0.00	0
	2020-2025	1.59	8.86	3.76	17.34	3.70	32.52	0.18	2.24	0.00	0
	2095-2100	0.11	2.85	1.89	3.36	1.63	9.31	0.63	2.33	0.03	0
COL	1950-1955	3.49	16.51	4.01	17.61	2.50	25.23	0.09	1.44	0.00	0
	2020-2025	0.90	5.20	2.82	12.32	3.61	30.07	0.27	2.90	0.00	0
	2095-2100	0.08	2.33	1.70	2.63	1.48	6.18	0.75	1.95	0.05	0
COM	1950-1955	1.31	8.42	2.77	8.50	1.49	10.87	0.05	0.70	0.00	0
	2020-2025	1.76	14.68	4.91	17.30	2.61	20.10	0.08	1.07	0.00	0
	2095-2100	0.19	5.32	2.72	6.08	1.89	16.62	0.16	1.86	0.00	0
CPV	1950-1955	1.81	15.19	4.08	17.80	2.32	24.03	0.04	1.03	0.00	0
	2020-2025	1.39	9.59	4.60	18.99	3.62	34.33	0.17	2.21	0.00	0
	2095-2100	0.07	2.49	1.73	2.99	1.57	8.88	0.68	2.39	0.04	0
CRI	1950-1955	3.91	19.44	4.53	21.81	3.08	33.18	0.11	1.78	0.00	0
	2020-2025	0.81	5.35	3.01	13.33	3.93	35.06	0.38	3.72	0.01	0
	2095-2100	0.03	1.73	1.54	2.64	1.57	6.79	0.82	2.17	0.06	0
CUB	1950-1955	2.93	8.15	2.85	8.51	2.11	13.72	0.16	1.40	0.00	0
	2020-2025	0.64	2.88	1.98	5.59	2.48	14.18	0.41	2.22	0.02	0
	2095-2100	0.01	1.69	1.50	2.70	1.63	4.84	0.81	1.59	0.06	0
CYM	1950-1955	2.43	9.61	2.97	9.71	1.89	14.06	0.08	1.02	0.00	0
	2020-2025	0.24	3.34	2.21	6.67	2.66	16.61	0.23	1.92	0.00	0
	2095-2100	0.00	0.89	1.30	2.09	1.35	4.74	0.34	1.09	0.01	0
CYP	1950-1955	0.41	6.34	2.64	7.33	1.68	10.65	0.06	0.72	0.00	0
	2020-2025	0.11	3.22	2.17	6.07	2.24	12.50	0.29	1.78	0.00	0
	2095-2100	0.00	0.85	1.37	2.26	1.51	6.23	0.55	1.79	0.01	0
CZE	1950-1955	0.92	5.29	2.29	5.49	1.65	8.55	0.11	0.84	0.00	0
	2020-2025	0.22	2.88	1.83	3.60	1.53	7.50	0.25	1.26	0.00	0
	2095-2100	0.02	2.11	1.68	2.88	1.60	5.74	0.60	1.75	0.01	0
DEU	1950-1955	0.27	3.12	1.80	3.35	1.36	5.46	0.13	0.79	0.00	0
	2020-2025	0.06	2.04	1.52	2.90	1.61	5.89	0.37	1.35	0.00	0
	2095-2100	0.00	1.46	1.54	2.56	1.55	4.78	0.64	1.49	0.01	0

Table S4. Living kin for a female Focal aged 65-69. Country-level averages for selected years (values for 2095-2100 refer to the median of the 1,000 country-level projections). Relative codes: ggd = great-grandchildren, gd = grandchildren, d = children, n = niblings, s = siblings, c = cousins, m = parents, a = aunts/uncles, gm = grandparents, ggm = great-grandparents. ***(continued)***

ISO	Year	ggd	gd	d	n	s	c	m	a	gm	ggm
DJI	1950-1955	0.94	11.54	3.61	13.80	2.08	18.78	0.05	0.95	0.00	0
	2020-2025	0.40	9.27	4.36	16.25	2.77	24.63	0.09	1.40	0.00	0
	2095-2100	0.05	3.16	1.99	3.37	1.41	10.17	0.16	1.31	0.00	0
DMA	1950-1955	1.48	9.13	2.94	9.49	1.76	13.11	0.07	0.90	0.00	0
	2020-2025	0.57	4.71	2.75	9.95	2.81	18.48	0.16	1.61	0.00	0
	2095-2100	0.05	2.12	1.63	2.38	1.33	5.80	0.50	1.51	0.02	0
DNK	1950-1955	0.56	4.71	2.23	5.09	1.75	8.41	0.18	1.05	0.00	0
	2020-2025	0.06	2.78	1.70	3.86	1.78	8.07	0.33	1.46	0.00	0
	2095-2100	0.00	2.05	1.71	3.01	1.69	6.38	0.64	1.94	0.00	0
DOM	1950-1955	3.43	18.22	4.27	19.81	2.70	28.78	0.08	1.49	0.00	0
	2020-2025	1.72	6.86	3.06	13.53	3.56	32.00	0.27	2.84	0.01	0
	2095-2100	0.09	2.80	1.86	3.35	1.65	7.76	0.67	2.16	0.05	0
DZA	1950-1955	2.39	15.54	3.93	16.84	2.38	23.47	0.06	1.21	0.00	0
	2020-2025	0.47	9.85	4.92	18.03	3.61	34.06	0.18	2.36	0.00	0
	2095-2100	0.11	3.66	2.13	4.09	1.90	12.80	0.64	2.78	0.01	0
ECU	1950-1955	3.26	17.30	4.25	19.29	2.72	28.11	0.08	1.45	0.00	0
	2020-2025	1.23	7.46	3.48	15.14	3.81	34.01	0.30	2.99	0.01	0
	2095-2100	0.07	2.42	1.72	3.11	1.66	8.39	0.78	2.48	0.06	0
EGY	1950-1955	2.33	10.62	3.25	11.34	2.01	16.15	0.08	1.03	0.00	0
	2020-2025	1.88	10.85	3.99	14.06	2.75	21.77	0.12	1.48	0.00	0
	2095-2100	0.33	4.30	2.29	4.57	1.85	11.92	0.46	2.38	0.01	0
ERI	1950-1955	1.07	7.94	2.75	8.29	1.38	10.36	0.03	0.60	0.00	0
	2020-2025	1.50	12.43	4.20	13.57	2.31	17.12	0.11	1.21	0.00	0
	2095-2100	0.28	4.88	2.47	4.78	1.84	14.56	0.44	2.81	0.01	0
ESH	1950-1955	1.71	10.01	2.93	9.78	1.38	11.44	0.02	0.51	0.00	0
	2020-2025	0.77	7.76	3.81	11.74	2.26	17.46	0.09	1.07	0.00	0
	2095-2100	0.07	2.70	1.87	3.32	1.54	9.48	0.34	1.68	0.00	0
ESP	1950-1955	0.08	3.79	2.15	4.75	1.59	7.62	0.10	0.80	0.00	0
	2020-2025	0.03	2.25	1.97	4.12	2.01	9.05	0.32	1.52	0.00	0
	2095-2100	0.01	1.19	1.38	2.04	1.37	4.37	0.64	1.50	0.01	0
EST	1950-1955	0.24	3.81	1.99	4.18	1.38	6.45	0.09	0.71	0.00	0
	2020-2025	0.18	2.78	1.74	2.98	1.18	5.46	0.18	0.91	0.00	0
	2095-2100	0.01	1.91	1.64	2.81	1.53	5.52	0.53	1.56	0.03	0
ETH	1950-1955	1.12	10.26	3.38	12.02	1.81	15.64	0.04	0.81	0.00	0
	2020-2025	2.32	14.05	4.15	14.92	2.25	19.34	0.08	1.21	0.00	0
	2095-2100	0.28	5.67	2.66	5.43	1.85	16.07	0.33	2.64	0.01	0
FIN	1950-1955	0.53	6.33	2.65	7.32	1.78	10.91	0.07	0.81	0.00	0
	2020-2025	0.06	2.59	1.69	4.14	1.88	9.90	0.30	1.71	0.00	0
	2095-2100	0.01	1.55	1.47	2.39	1.49	5.47	0.65	1.81	0.01	0

Table S4. Living kin for a female Focal aged 65-69. Country-level averages for selected years (values for 2095-2100 refer to the median of the 1,000 country-level projections). Relative codes: ggd = great-grandchildren, gd = grandchildren, d = children, n = niblings, s = siblings, c = cousins, m = parents, a = aunts/uncles, gm = grandparents, ggm = great-grandparents. ***(continued)***

ISO	Year	ggd	gd	d	n	s	c	m	a	gm	ggm
FJI	1950-1955	6.73	21.98	4.67	23.54	2.42	31.11	0.01	0.77	0.00	0
	2020-2025	1.18	8.22	3.17	15.53	3.17	31.85	0.10	1.97	0.00	0
	2095-2100	0.09	3.19	2.03	3.80	1.52	9.23	0.20	1.36	0.00	0
FLK	1950-1955	0.49	4.48	1.94	4.25	1.18	5.88	0.07	0.53	0.00	0
	2020-2025	0.12	2.41	1.81	3.58	1.70	7.62	0.21	1.08	0.00	0
	2095-2100	0.02	1.84	1.58	2.49	1.51	4.58	0.70	1.48	0.02	0
FRA	1950-1955	0.59	5.64	2.42	6.11	1.74	9.58	0.11	0.92	0.00	0
	2020-2025	0.11	3.43	1.98	4.69	1.96	9.86	0.41	1.82	0.01	0
	2095-2100	0.02	2.22	1.74	3.09	1.67	6.69	0.76	2.10	0.02	0
FRO	1950-1955	0.61	8.30	3.05	9.66	2.01	14.17	0.07	0.87	0.00	0
	2020-2025	0.36	5.80	2.57	8.14	2.64	16.64	0.26	1.87	0.00	0
	2095-2100	0.02	4.39	2.37	4.80	2.09	10.88	0.71	2.71	0.01	0
FSM	1950-1955	2.78	17.73	4.27	19.69	2.54	27.30	0.06	1.21	0.00	0
	2020-2025	1.18	12.70	4.96	22.59	3.93	39.68	0.13	2.09	0.00	0
	2095-2100	0.06	3.27	2.08	3.83	1.68	12.10	0.35	2.20	0.01	0
GAB	1950-1955	0.83	4.24	1.89	4.04	1.11	5.37	0.08	0.57	0.00	0
	2020-2025	2.46	11.93	3.94	10.42	1.89	10.37	0.15	0.89	0.00	0
	2095-2100	0.30	4.66	2.36	4.73	1.62	12.21	0.24	1.80	0.01	0
GBR	1950-1955	0.30	3.71	1.99	4.06	1.43	6.29	0.10	0.65	0.00	0
	2020-2025	0.14	2.94	1.82	4.00	1.85	7.59	0.35	1.34	0.00	0
	2095-2100	0.01	1.78	1.59	2.80	1.64	6.01	0.72	1.95	0.02	0
GEO	1950-1955	0.33	4.71	2.16	5.05	1.41	7.47	0.07	0.70	0.00	0
	2020-2025	0.58	3.12	1.86	4.09	1.53	7.57	0.11	0.95	0.00	0
	2095-2100	0.03	2.44	1.97	3.74	1.72	6.54	0.43	1.49	0.01	0
GGY	1950-1955	0.32	3.91	2.04	4.26	1.53	6.67	0.13	0.68	0.00	0
	2020-2025	0.09	2.23	1.65	3.19	1.69	6.91	0.32	1.20	0.00	0
	2095-2100	0.01	1.54	1.52	2.46	1.54	4.90	0.75	1.69	0.03	0
GHA	1950-1955	2.17	12.83	3.68	14.49	2.19	20.41	0.07	1.18	0.00	0
	2020-2025	1.54	11.91	4.18	16.49	2.87	25.89	0.12	1.73	0.00	0
	2095-2100	0.23	4.80	2.42	4.76	1.64	12.56	0.21	1.73	0.00	0
GIN	1950-1955	1.46	7.19	2.58	7.31	1.43	9.60	0.05	0.70	0.00	0
	2020-2025	2.47	12.18	3.71	11.53	2.01	13.84	0.12	1.10	0.00	0
	2095-2100	0.40	5.40	2.52	4.97	1.59	13.05	0.19	1.81	0.00	0
GLP	1950-1955	1.69	14.66	3.84	16.15	1.91	19.83	0.03	0.66	0.00	0
	2020-2025	0.24	4.97	2.62	10.88	3.50	27.50	0.35	2.81	0.01	0
	2095-2100	0.02	2.35	1.85	3.33	1.74	7.91	0.72	2.27	0.03	0
GMB	1950-1955	2.18	7.15	2.40	6.62	1.33	8.76	0.05	0.70	0.00	0
	2020-2025	2.94	15.10	3.99	12.56	2.03	14.18	0.14	1.21	0.00	0
	2095-2100	0.15	5.65	2.81	5.96	1.89	17.62	0.22	2.39	0.00	0

Table S4. Living kin for a female Focal aged 65-69. Country-level averages for selected years (values for 2095-2100 refer to the median of the 1,000 country-level projections). Relative codes: ggd = great-grandchildren, gd = grandchildren, d = children, n = niblings, s = siblings, c = cousins, m = parents, a = aunts/uncles, gm = grandparents, ggm = great-grandparents. ***(continued)***

ISO	Year	ggd	gd	d	n	s	c	m	a	gm	ggm
GNB	1950-1955	0.94	7.14	2.59	7.34	1.42	9.36	0.05	0.61	0.00	0
	2020-2025	1.62	11.60	3.79	11.15	1.72	12.12	0.07	0.75	0.00	0
	2095-2100	0.27	4.79	2.38	4.69	1.54	12.59	0.17	1.68	0.00	0
GNQ	1950-1955	1.82	8.33	2.74	8.33	1.55	11.01	0.06	0.75	0.00	0
	2020-2025	3.03	12.18	3.72	11.72	1.94	14.28	0.09	1.01	0.00	0
	2095-2100	0.44	4.49	2.30	4.47	1.49	11.82	0.19	1.62	0.00	0
GRC	1950-1955	0.13	3.81	2.11	4.56	1.58	7.43	0.11	0.85	0.00	0
	2020-2025	0.06	2.51	1.89	3.60	1.67	7.44	0.34	1.50	0.00	0
	2095-2100	0.00	1.06	1.42	2.21	1.45	4.89	0.65	1.61	0.02	0
GRD	1950-1955	4.85	16.04	3.86	16.52	2.41	23.97	0.08	1.35	0.00	0
	2020-2025	0.73	6.24	3.19	14.17	3.59	29.24	0.29	2.85	0.01	0
	2095-2100	0.06	2.46	1.78	3.09	1.57	8.14	0.54	1.96	0.02	0
GRL	1950-1955	1.95	14.11	3.68	14.99	1.99	19.65	0.05	0.94	0.00	0
	2020-2025	0.66	4.23	2.00	8.63	2.88	21.52	0.14	1.99	0.00	0
	2095-2100	0.06	2.46	1.78	3.17	1.53	6.56	0.41	1.45	0.01	0
GTM	1950-1955	3.26	14.00	3.60	14.36	2.08	19.61	0.08	1.12	0.00	0
	2020-2025	2.20	11.99	4.39	17.34	2.96	26.96	0.26	2.15	0.01	0
	2095-2100	0.16	2.95	1.81	3.17	1.54	10.26	0.62	2.66	0.04	0
GUF	1950-1955	2.04	10.14	2.98	9.97	1.63	12.67	0.05	0.67	0.00	0
	2020-2025	1.79	9.92	3.23	12.01	2.81	19.26	0.19	1.61	0.00	0
	2095-2100	0.06	5.19	2.85	6.66	2.44	17.01	0.65	3.41	0.03	0
GUM	1950-1955	3.79	19.07	4.51	21.75	2.37	28.11	0.01	0.57	0.00	0
	2020-2025	1.23	7.98	2.98	13.44	3.46	31.94	0.34	3.06	0.01	0
	2095-2100	0.04	3.17	2.18	4.42	1.97	11.31	0.74	2.85	0.06	0
GUY	1950-1955	5.74	18.16	3.99	18.01	2.12	23.31	0.04	0.85	0.00	0
	2020-2025	1.53	7.00	2.90	13.15	3.05	27.25	0.16	1.84	0.00	0
	2095-2100	0.12	3.10	1.93	3.47	1.47	7.54	0.37	1.56	0.02	0
HKG	1950-1955	1.22	11.87	3.51	13.12	2.00	17.69	0.02	0.61	0.00	0
	2020-2025	0.02	2.06	2.03	7.07	3.46	23.66	0.41	2.86	0.01	0
	2095-2100	0.00	0.54	0.96	1.24	1.11	3.22	0.67	1.25	0.02	0
HND	1950-1955	3.63	17.15	3.99	17.65	2.39	24.48	0.07	1.24	0.00	0
	2020-2025	2.37	12.27	4.79	20.94	3.81	36.52	0.15	2.16	0.00	0
	2095-2100	0.20	3.00	1.84	3.21	1.56	9.86	0.52	2.29	0.02	0
HRV	1950-1955	0.49	4.48	2.11	4.73	1.45	7.24	0.08	0.73	0.00	0
	2020-2025	0.13	2.59	1.68	3.19	1.42	6.52	0.22	1.20	0.00	0
	2095-2100	0.00	1.20	1.47	2.51	1.56	5.37	0.55	1.55	0.01	0
HTI	1950-1955	0.81	8.45	3.13	10.22	1.76	13.71	0.05	0.80	0.00	0
	2020-2025	0.82	8.63	3.73	12.66	2.34	17.87	0.08	1.14	0.00	0
	2095-2100	0.12	3.35	2.01	3.51	1.41	9.38	0.17	1.27	0.00	0

Table S4. Living kin for a female Focal aged 65-69. Country-level averages for selected years (values for 2095-2100 refer to the median of the 1,000 country-level projections). Relative codes: ggd = great-grandchildren, gd = grandchildren, d = children, n = niblings, s = siblings, c = cousins, m = parents, a = aunts/uncles, gm = grandparents, ggm = great-grandparents. *(continued)*

ISO	Year	ggd	gd	d	n	s	c	m	a	gm	ggm
HUN	1950-1955	0.62	4.09	2.01	4.24	1.45	6.65	0.11	0.78	0.00	0
	2020-2025	0.18	2.42	1.66	2.96	1.17	5.38	0.19	0.90	0.00	0
	2095-2100	0.02	1.66	1.59	2.52	1.44	4.95	0.50	1.42	0.01	0
IDN	1950-1955	1.40	7.83	2.64	7.80	1.58	10.83	0.07	0.86	0.00	0
	2020-2025	0.86	6.52	3.17	9.67	2.52	16.33	0.14	1.41	0.00	0
	2095-2100	0.07	2.88	1.83	3.20	1.48	7.34	0.36	1.52	0.01	0
IMN	1950-1955	0.24	3.16	1.72	3.18	1.24	4.89	0.14	0.68	0.00	0
	2020-2025	0.12	2.84	1.85	3.56	1.66	6.42	0.34	1.17	0.00	0
	2095-2100	0.02	1.83	1.56	2.51	1.52	5.32	0.83	1.88	0.04	0
IND	1950-1955	2.41	10.79	3.16	11.03	1.88	15.18	0.07	0.95	0.00	0
	2020-2025	1.60	7.73	3.14	10.83	2.60	19.10	0.18	1.66	0.00	0
	2095-2100	0.04	2.68	1.84	3.38	1.66	8.81	0.59	2.25	0.03	0
IRL	1950-1955	0.12	7.05	3.14	10.00	2.30	15.76	0.08	0.97	0.00	0
	2020-2025	0.06	4.22	2.70	8.03	2.79	17.90	0.22	1.78	0.00	0
	2095-2100	0.01	1.80	1.70	2.90	1.66	7.41	0.66	2.19	0.01	0
IRN	1950-1955	1.97	13.54	3.73	14.94	2.24	20.87	0.06	1.07	0.00	0
	2020-2025	0.73	7.05	4.37	15.52	3.48	30.29	0.15	2.01	0.00	0
	2095-2100	0.02	1.98	1.64	2.84	1.60	7.89	0.64	1.89	0.03	0
IRQ	1950-1955	1.81	13.09	3.55	13.77	2.01	18.16	0.05	0.86	0.00	0
	2020-2025	2.09	16.26	5.24	20.38	3.06	28.19	0.10	1.36	0.00	0
	2095-2100	0.37	5.21	2.54	5.39	1.96	15.82	0.37	2.55	0.01	0
ISL	1950-1955	1.51	9.94	3.30	11.27	2.59	18.71	0.22	1.78	0.00	0
	2020-2025	0.19	4.15	2.29	7.49	2.87	18.44	0.44	2.73	0.01	0
	2095-2100	0.01	1.85	1.67	3.00	1.78	7.64	0.68	2.27	0.01	0
ISR	1950-1955	3.52	14.57	3.91	15.76	2.92	25.31	0.14	1.65	0.00	0
	2020-2025	0.43	8.03	3.16	11.95	3.27	25.92	0.45	3.11	0.01	0
	2095-2100	0.04	4.73	2.58	5.78	2.28	14.27	0.84	3.54	0.05	0
ITA	1950-1955	0.13	3.33	1.98	4.00	1.49	6.48	0.11	0.78	0.00	0
	2020-2025	0.03	2.02	1.70	3.24	1.75	7.07	0.34	1.43	0.00	0
	2095-2100	0.00	1.08	1.38	2.13	1.45	4.45	0.67	1.55	0.01	0
JAM	1950-1955	2.16	8.84	2.93	9.21	2.04	14.11	0.13	1.25	0.00	0
	2020-2025	0.91	5.56	3.14	11.19	3.13	20.11	0.22	1.79	0.00	0
	2095-2100	0.03	1.58	1.39	2.09	1.23	5.16	0.39	1.25	0.01	0
JEY	1950-1955	0.25	3.22	1.85	3.50	1.41	5.69	0.15	0.78	0.00	0
	2020-2025	0.09	2.15	1.51	2.99	1.65	6.20	0.33	1.23	0.00	0
	2095-2100	0.01	1.66	1.50	2.42	1.51	4.86	0.66	1.59	0.02	0
JOR	1950-1955	2.58	17.22	4.18	19.02	2.37	25.88	0.05	1.16	0.00	0
	2020-2025	1.41	16.42	5.75	27.78	4.76	47.90	0.19	2.82	0.00	0
	2095-2100	0.08	3.75	2.30	4.48	1.97	15.66	0.63	3.34	0.02	0

Table S4. Living kin for a female Focal aged 65-69. Country-level averages for selected years (values for 2095-2100 refer to the median of the 1,000 country-level projections). Relative codes: ggd = great-grandchildren, gd = grandchildren, d = children, n = niblings, s = siblings, c = cousins, m = parents, a = aunts/uncles, gm = grandparents, ggm = great-grandparents. ***(continued)***

ISO	Year	ggd	gd	d	n	s	c	m	a	gm	ggm
JPN	1950-1955	0.26	5.82	2.48	6.45	1.77	10.18	0.09	0.87	0.00	0
	2020-2025	0.03	2.41	1.90	3.60	1.75	9.23	0.42	2.06	0.00	0
	2095-2100	0.00	1.11	1.41	2.27	1.51	4.79	0.73	1.67	0.01	0
KAZ	1950-1955	1.65	10.66	3.12	10.86	1.96	15.61	0.07	0.99	0.00	0
	2020-2025	0.79	5.54	2.42	7.63	2.04	14.83	0.09	1.15	0.00	0
	2095-2100	0.18	4.95	2.62	5.57	1.90	9.83	0.35	1.73	0.01	0
KEN	1950-1955	3.01	13.05	3.48	13.38	1.94	18.02	0.07	1.06	0.00	0
	2020-2025	3.41	16.17	4.83	21.78	2.98	28.57	0.16	1.66	0.00	0
	2095-2100	0.28	4.72	2.31	4.35	1.49	11.99	0.25	1.75	0.01	0
KGZ	1950-1955	1.74	10.73	3.15	11.09	1.89	15.57	0.06	0.92	0.00	0
	2020-2025	1.56	8.77	3.33	12.03	2.63	20.09	0.10	1.37	0.00	0
	2095-2100	0.45	4.57	2.31	4.67	1.79	10.67	0.40	2.05	0.01	0
KHM	1950-1955	1.76	12.42	3.32	12.42	1.75	15.69	0.03	0.73	0.00	0
	2020-2025	0.92	7.88	3.70	11.04	2.16	17.69	0.10	1.22	0.00	0
	2095-2100	0.26	3.15	1.82	3.07	1.44	8.27	0.51	1.90	0.01	0
KIR	1950-1955	2.07	14.14	3.72	15.17	2.13	20.29	0.05	0.94	0.00	0
	2020-2025	0.95	10.59	3.93	16.04	3.01	26.45	0.11	1.56	0.00	0
	2095-2100	0.15	5.07	2.52	5.12	1.78	12.86	0.20	1.63	0.00	0
KNA	1950-1955	7.73	21.80	4.48	21.95	2.70	29.97	0.07	1.24	0.00	0
	2020-2025	0.63	4.49	2.67	12.97	3.33	30.62	0.14	1.91	0.00	0
	2095-2100	0.06	1.94	1.52	2.22	1.22	4.93	0.42	1.22	0.01	0
KOR	1950-1955	0.59	8.82	2.39	7.05	0.85	6.66	0.01	0.22	0.00	0
	2020-2025	0.03	2.91	2.15	8.25	3.17	18.31	0.23	1.76	0.00	0
	2095-2100	0.00	0.71	1.08	1.52	1.20	4.34	0.55	1.56	0.01	0
KWT	1950-1955	4.84	19.36	4.30	20.05	2.77	29.05	0.08	1.31	0.00	0
	2020-2025	0.73	9.91	4.81	21.22	4.64	44.99	0.29	2.97	0.00	0
	2095-2100	0.04	3.51	2.07	3.76	1.77	10.40	0.90	2.65	0.02	0
LAO	1950-1955	1.46	10.42	3.18	11.09	1.71	14.28	0.04	0.74	0.00	0
	2020-2025	1.63	10.12	4.21	14.02	2.50	20.29	0.09	1.21	0.00	0
	2095-2100	0.26	3.36	1.91	3.28	1.47	8.93	0.42	1.87	0.01	0
LBN	1950-1955	3.41	18.33	4.56	21.60	3.26	34.14	0.12	1.96	0.00	0
	2020-2025	0.52	6.16	3.24	13.35	3.46	32.26	0.23	2.68	0.00	0
	2095-2100	0.06	2.68	1.85	3.20	1.68	8.36	0.80	2.50	0.03	0
LBR	1950-1955	1.80	8.92	2.87	9.12	1.62	12.22	0.05	0.81	0.00	0
	2020-2025	2.49	12.06	3.82	12.89	2.17	16.53	0.10	1.14	0.00	0
	2095-2100	0.54	5.01	2.46	4.92	1.62	12.86	0.22	1.84	0.00	0
LBY	1950-1955	0.80	9.01	2.97	9.61	1.61	12.34	0.05	0.72	0.00	0
	2020-2025	0.29	10.69	5.79	19.90	3.65	28.66	0.14	1.56	0.00	0
	2095-2100	0.08	2.72	1.87	3.26	1.58	11.65	0.43	2.02	0.00	0

Table S4. Living kin for a female Focal aged 65-69. Country-level averages for selected years (values for 2095-2100 refer to the median of the 1,000 country-level projections). Relative codes: ggd = great-grandchildren, gd = grandchildren, d = children, n = niblings, s = siblings, c = cousins, m = parents, a = aunts/uncles, gm = grandparents, ggm = great-grandparents. ***(continued)***

ISO	Year	ggd	gd	d	n	s	c	m	a	gm	ggm
LCA	1950-1955	3.25	13.61	3.56	13.93	2.27	20.09	0.08	1.19	0.00	0
	2020-2025	0.82	5.86	3.64	15.08	3.64	29.13	0.18	2.05	0.00	0
	2095-2100	0.04	1.69	1.45	2.06	1.25	5.42	0.47	1.34	0.01	0
LIE	1950-1955	0.18	3.92	1.97	4.17	1.26	5.92	0.07	0.51	0.00	0
	2020-2025	0.03	2.26	1.70	3.44	1.79	7.47	0.30	1.31	0.00	0
	2095-2100	0.00	1.48	1.51	2.38	1.51	4.98	0.76	1.76	0.02	0
LKA	1950-1955	2.56	15.38	3.93	16.50	2.60	24.59	0.08	1.31	0.00	0
	2020-2025	0.26	5.32	2.81	10.94	3.05	25.11	0.15	1.91	0.00	0
	2095-2100	0.05	2.58	1.80	3.13	1.63	7.39	0.60	1.95	0.01	0
LSO	1950-1955	1.30	10.04	3.25	11.32	1.77	14.98	0.04	0.84	0.00	0
	2020-2025	1.35	8.51	3.22	11.04	1.46	13.67	0.06	0.73	0.00	0
	2095-2100	0.27	3.36	1.84	3.02	1.00	5.95	0.10	0.71	0.00	0
LTU	1950-1955	0.32	5.29	2.39	6.03	1.69	9.40	0.10	0.93	0.00	0
	2020-2025	0.19	2.74	1.76	3.73	1.43	7.50	0.17	1.05	0.00	0
	2095-2100	0.00	1.70	1.61	2.95	1.58	5.32	0.45	1.31	0.02	0
LUX	1950-1955	0.20	3.48	1.89	3.74	1.36	5.84	0.10	0.68	0.00	0
	2020-2025	0.04	2.29	1.57	3.08	1.57	6.21	0.33	1.31	0.00	0
	2095-2100	0.00	1.37	1.46	2.34	1.46	5.14	0.63	1.66	0.02	0
LVA	1950-1955	0.12	2.79	1.75	3.20	1.25	5.01	0.10	0.68	0.00	0
	2020-2025	0.18	2.37	1.61	2.58	1.02	4.23	0.14	0.66	0.00	0
	2095-2100	0.02	1.80	1.56	2.61	1.42	4.76	0.45	1.26	0.02	0
MAC	1950-1955	1.21	17.00	4.49	20.84	2.95	30.65	0.06	1.17	0.00	0
	2020-2025	0.02	1.38	1.57	6.57	3.53	27.19	0.36	3.33	0.00	0
	2095-2100	0.01	1.42	1.34	1.96	1.34	3.68	0.76	1.26	0.02	0
MAF	1950-1955	2.80	20.61	4.98	25.46	3.56	39.90	0.11	1.92	0.00	0
	2020-2025	0.52	7.96	3.64	17.01	4.31	42.58	0.24	3.02	0.00	0
	2095-2100	0.02	2.94	2.09	4.13	1.97	11.13	0.68	2.44	0.02	0
MAR	1950-1955	3.43	12.25	3.28	12.22	1.76	16.06	0.05	0.88	0.00	0
	2020-2025	0.63	8.03	4.23	14.93	3.39	27.51	0.16	2.03	0.00	0
	2095-2100	0.06	2.71	1.91	3.38	1.71	9.77	0.59	2.28	0.01	0
MCO	1950-1955	0.48	6.58	2.60	7.02	1.88	10.82	0.10	0.82	0.00	0
	2020-2025	0.07	3.54	1.96	5.17	2.35	12.36	0.40	1.90	0.00	0
	2095-2100	0.01	2.94	2.02	3.70	1.84	7.37	0.97	2.44	0.03	0
MDA	1950-1955	0.59	6.29	2.55	6.98	1.54	9.75	0.05	0.68	0.00	0
	2020-2025	0.43	3.26	1.96	4.68	1.48	8.50	0.08	0.85	0.00	0
	2095-2100	0.02	2.19	1.72	3.17	1.52	5.59	0.28	1.12	0.00	0
MDG	1950-1955	3.78	16.20	3.66	15.29	1.92	19.30	0.04	0.82	0.00	0
	2020-2025	3.43	14.66	4.45	18.92	3.08	28.43	0.13	1.69	0.00	0
	2095-2100	0.57	5.27	2.52	5.19	1.80	14.17	0.32	2.24	0.01	0

Table S4. Living kin for a female Focal aged 65-69. Country-level averages for selected years (values for 2095-2100 refer to the median of the 1,000 country-level projections). Relative codes: ggd = great-grandchildren, gd = grandchildren, d = children, n = niblings, s = siblings, c = cousins, m = parents, a = aunts/uncles, gm = grandparents, ggm = great-grandparents. ***(continued)***

ISO	Year	ggd	gd	d	n	s	c	m	a	gm	ggm
MDV	1950-1955	1.87	9.00	2.88	9.06	1.85	13.33	0.08	1.05	0.00	0
	2020-2025	1.22	10.80	5.41	16.31	3.33	25.12	0.29	2.24	0.01	0
	2095-2100	0.02	1.96	1.64	3.03	1.75	11.31	0.73	2.84	0.04	0
MEX	1950-1955	3.31	16.16	3.89	16.84	2.31	23.23	0.08	1.24	0.00	0
	2020-2025	1.26	7.46	3.58	15.20	3.69	32.32	0.27	2.61	0.01	0
	2095-2100	0.10	2.32	1.60	2.70	1.48	6.95	0.68	2.06	0.03	0
MHL	1950-1955	8.67	28.92	5.04	28.65	2.84	37.84	0.06	1.30	0.00	0
	2020-2025	4.24	19.13	5.33	29.79	3.78	47.96	0.09	1.82	0.00	0
	2095-2100	0.11	3.10	1.91	3.43	1.37	10.77	0.17	1.37	0.00	0
MKD	1950-1955	0.79	10.73	3.59	13.23	2.60	21.23	0.10	1.44	0.00	0
	2020-2025	0.28	3.33	2.00	5.83	2.17	15.50	0.15	1.83	0.00	0
	2095-2100	0.01	1.46	1.47	2.39	1.51	5.62	0.58	1.68	0.01	0
MLI	1950-1955	1.61	7.06	2.40	6.55	1.18	7.85	0.02	0.45	0.00	0
	2020-2025	3.64	16.32	4.17	12.93	1.96	13.48	0.07	0.89	0.00	0
	2095-2100	0.67	8.99	3.34	7.50	1.90	19.37	0.16	2.24	0.00	0
MLT	1950-1955	0.83	10.74	3.60	13.23	2.48	20.35	0.05	1.02	0.00	0
	2020-2025	0.06	2.55	1.97	6.06	2.65	17.67	0.33	2.58	0.00	0
	2095-2100	0.00	1.04	1.39	2.30	1.50	5.29	0.64	1.64	0.02	0
MMR	1950-1955	0.72	8.48	2.92	9.30	1.52	11.61	0.03	0.58	0.00	0
	2020-2025	0.45	5.66	3.14	10.20	2.39	16.83	0.07	1.02	0.00	0
	2095-2100	0.11	2.76	1.77	2.83	1.29	6.21	0.23	1.07	0.00	0
MNE	1950-1955	0.82	11.25	3.68	13.99	2.61	22.33	0.09	1.49	0.00	0
	2020-2025	0.21	3.48	2.05	5.87	2.22	16.23	0.19	2.11	0.00	0
	2095-2100	0.01	1.78	1.61	2.86	1.66	6.29	0.51	1.67	0.01	0
MNG	1950-1955	0.78	9.59	3.17	10.75	1.47	12.66	0.01	0.36	0.00	0
	2020-2025	1.02	8.84	3.92	14.44	2.92	22.54	0.08	1.06	0.00	0
	2095-2100	0.19	3.47	2.11	4.09	1.78	9.62	0.55	2.18	0.02	0
MNP	1950-1955	4.03	21.10	4.68	23.43	2.80	32.40	0.06	1.23	0.00	0
	2020-2025	1.05	7.83	3.63	15.74	4.00	39.61	0.22	2.67	0.00	0
	2095-2100	0.04	2.79	1.91	3.61	1.81	9.07	0.68	2.07	0.02	0
MOZ	1950-1955	1.56	8.46	2.87	8.91	1.61	11.66	0.05	0.76	0.00	0
	2020-2025	2.88	11.95	3.52	11.93	1.80	13.68	0.06	0.80	0.00	0
	2095-2100	0.73	5.57	2.63	5.42	1.65	13.43	0.18	1.81	0.00	0
MRT	1950-1955	1.37	9.49	3.12	10.48	1.73	13.93	0.06	0.87	0.00	0
	2020-2025	2.25	14.68	4.73	18.21	2.92	23.68	0.10	1.43	0.00	0
	2095-2100	0.43	6.58	2.93	6.53	2.01	18.03	0.26	2.35	0.00	0
MSR	1950-1955	3.39	10.78	2.98	10.21	1.73	13.78	0.07	0.89	0.00	0
	2020-2025	0.42	2.92	2.35	7.32	2.70	17.76	0.26	2.06	0.01	0
	2095-2100	0.06	2.18	1.66	2.35	1.32	4.22	0.64	1.27	0.03	0

Table S4. Living kin for a female Focal aged 65-69. Country-level averages for selected years (values for 2095-2100 refer to the median of the 1,000 country-level projections). Relative codes: ggd = great-grandchildren, gd = grandchildren, d = children, n = niblings, s = siblings, c = cousins, m = parents, a = aunts/uncles, gm = grandparents, ggm = great-grandparents. ***(continued)***

ISO	Year	ggd	gd	d	n	s	c	m	a	gm	ggm
	1950-1955	1.85	17.41	4.35	20.23	2.30	25.98	0.02	0.76	0.00	0
MTQ	2020-2025	0.20	3.88	2.34	10.25	3.71	31.13	0.36	3.29	0.01	0
	2095-2100	0.01	2.20	1.82	3.25	1.74	7.05	0.79	2.13	0.03	0
	1950-1955	3.80	17.25	4.14	18.59	1.92	22.56	0.01	0.48	0.00	0
MUS	2020-2025	0.32	3.76	2.28	9.73	3.17	26.38	0.20	2.24	0.00	0
	2095-2100	0.01	1.36	1.42	2.28	1.40	5.19	0.50	1.44	0.02	0
	1950-1955	1.38	7.95	2.75	8.24	1.59	11.23	0.05	0.83	0.00	0
MWI	2020-2025	2.76	13.10	3.80	12.57	1.81	14.33	0.09	0.93	0.00	0
	2095-2100	0.64	4.90	2.35	4.58	1.55	12.53	0.27	1.98	0.01	0
	1950-1955	3.05	18.18	4.23	19.49	2.18	24.84	0.02	0.73	0.00	0
MYS	2020-2025	0.26	6.93	3.71	16.69	3.77	34.71	0.22	2.57	0.00	0
	2095-2100	0.01	1.97	1.67	2.84	1.57	9.03	0.51	2.10	0.01	0
	1950-1955	3.42	16.00	4.12	18.05	2.41	24.79	0.06	1.20	0.00	0
MYT	2020-2025	4.02	20.91	5.82	28.54	4.35	43.00	0.24	2.68	0.00	0
	2095-2100	0.17	8.54	3.38	8.69	2.71	26.79	0.74	4.57	0.04	0
	1950-1955	1.18	9.29	3.10	10.34	1.81	14.01	0.06	0.90	0.00	0
NAM	2020-2025	1.55	10.76	3.94	14.18	2.06	17.39	0.09	0.95	0.00	0
	2095-2100	0.22	4.37	2.33	4.53	1.46	10.05	0.15	1.20	0.00	0
	1950-1955	1.80	11.57	3.34	12.18	1.88	16.10	0.05	0.82	0.00	0
NCL	2020-2025	0.62	6.35	3.16	11.75	3.07	22.70	0.30	2.23	0.01	0
	2095-2100	0.06	2.37	1.82	3.21	1.68	8.60	0.74	2.38	0.04	0
	1950-1955	3.73	12.99	3.39	12.84	1.82	16.86	0.03	0.86	0.00	0
NER	2020-2025	4.57	18.02	4.16	17.52	2.59	22.95	0.10	1.53	0.00	0
	2095-2100	1.45	13.83	4.21	11.38	2.50	28.44	0.23	3.17	0.00	0
	1950-1955	1.25	8.72	2.91	9.21	1.57	11.94	0.04	0.71	0.00	0
NGA	2020-2025	2.53	13.56	3.75	12.51	1.93	15.14	0.07	0.98	0.00	0
	2095-2100	0.50	6.71	2.79	5.40	1.52	13.44	0.14	1.59	0.00	0
	1950-1955	4.24	16.09	3.71	15.76	2.06	20.87	0.05	1.00	0.00	0
NIC	2020-2025	2.18	9.44	4.09	15.89	3.25	29.57	0.17	2.13	0.00	0
	2095-2100	0.18	2.99	1.87	3.28	1.59	8.47	0.65	2.30	0.03	0
	1950-1955	5.31	21.40	4.62	22.98	3.12	35.08	0.09	1.81	0.00	0
NIU	2020-2025	0.60	10.11	4.52	21.00	3.99	41.81	0.14	2.38	0.00	0
	2095-2100	0.06	2.91	1.97	3.52	1.59	10.22	0.30	1.60	0.00	0
	1950-1955	0.17	6.25	2.88	8.38	2.31	14.16	0.14	1.27	0.00	0
NLD	2020-2025	0.03	2.63	1.77	4.61	2.21	12.12	0.26	1.77	0.00	0
	2095-2100	0.00	1.85	1.63	2.75	1.62	5.80	0.65	1.81	0.00	0
	1950-1955	0.33	4.71	2.32	5.52	1.84	9.11	0.19	1.08	0.00	0
NOR	2020-2025	0.10	3.18	1.88	4.48	1.97	9.27	0.31	1.47	0.00	0
	2095-2100	0.01	1.73	1.56	2.67	1.63	6.36	0.71	2.11	0.01	0

Table S4. Living kin for a female Focal aged 65-69. Country-level averages for selected years (values for 2095-2100 refer to the median of the 1,000 country-level projections). Relative codes: ggd = great-grandchildren, gd = grandchildren, d = children, n = niblings, s = siblings, c = cousins, m = parents, a = aunts/uncles, gm = grandparents, ggm = great-grandparents. ***(continued)***

ISO	Year	ggd	gd	d	n	s	c	m	a	gm	ggm
NPL	1950-1955	1.77	9.39	2.90	9.41	1.63	12.48	0.05	0.77	0.00	0
	2020-2025	1.73	8.25	3.55	11.35	2.38	17.39	0.11	1.27	0.00	0
	2095-2100	0.28	2.84	1.64	2.78	1.50	7.70	0.61	2.07	0.02	0
NRU	1950-1955	0.54	11.78	3.81	15.16	2.33	21.36	0.05	0.90	0.00	0
	2020-2025	1.13	8.91	2.98	12.04	2.15	18.83	0.04	0.79	0.00	0
	2095-2100	0.55	5.42	2.47	5.12	1.49	10.11	0.16	1.23	0.00	0
NZL	1950-1955	1.31	9.95	3.24	10.81	2.42	17.32	0.15	1.32	0.00	0
	2020-2025	0.23	4.08	2.25	7.27	2.79	17.73	0.39	2.40	0.01	0
	2095-2100	0.01	1.99	1.64	2.91	1.70	7.09	0.77	2.26	0.04	0
OMN	1950-1955	2.09	7.18	2.49	6.94	1.42	9.35	0.08	0.85	0.00	0
	2020-2025	1.80	14.07	5.70	18.67	3.31	23.27	0.25	1.96	0.01	0
	2095-2100	0.04	2.76	2.03	4.14	2.04	17.65	0.60	3.31	0.02	0
PAK	1950-1955	2.05	11.11	3.38	12.23	2.04	17.19	0.07	1.06	0.00	0
	2020-2025	2.05	15.25	4.86	19.74	3.29	27.92	0.14	1.74	0.00	0
	2095-2100	0.29	5.10	2.41	4.66	1.66	13.56	0.22	1.84	0.00	0
PAN	1950-1955	4.23	16.05	3.99	17.10	2.71	26.05	0.15	1.74	0.00	0
	2020-2025	1.51	6.94	3.09	12.66	3.53	29.36	0.41	3.23	0.02	0
	2095-2100	0.13	3.04	1.92	3.46	1.71	8.37	0.84	2.55	0.08	0
PER	1950-1955	2.77	15.26	4.07	17.46	2.80	26.79	0.10	1.65	0.00	0
	2020-2025	0.94	7.35	3.63	14.70	3.63	31.37	0.24	2.67	0.00	0
	2095-2100	0.10	2.59	1.79	3.09	1.62	8.40	0.70	2.34	0.03	0
PHL	1950-1955	2.51	23.70	5.32	29.60	3.40	43.21	0.07	1.68	0.00	0
	2020-2025	1.06	10.02	4.01	21.05	4.15	46.13	0.13	2.54	0.00	0
	2095-2100	0.17	3.68	2.10	3.87	1.68	10.92	0.40	2.13	0.01	0
PLW	1950-1955	3.82	21.31	4.50	22.42	2.37	28.41	0.04	0.99	0.00	0
	2020-2025	0.50	4.97	2.89	12.40	2.75	27.90	0.07	1.37	0.00	0
	2095-2100	0.08	3.15	1.88	3.16	1.24	5.27	0.17	0.70	0.00	0
PNG	1950-1955	1.31	9.57	2.91	9.57	1.46	11.79	0.04	0.63	0.00	0
	2020-2025	1.58	11.61	4.32	15.70	2.68	21.08	0.08	1.15	0.00	0
	2095-2100	0.22	4.15	2.24	4.28	1.51	11.19	0.16	1.35	0.00	0
POL	1950-1955	0.82	7.87	2.87	8.68	1.98	13.29	0.08	0.99	0.00	0
	2020-2025	0.18	2.71	1.94	4.81	1.82	11.00	0.22	1.54	0.00	0
	2095-2100	0.01	1.65	1.53	2.46	1.45	4.92	0.56	1.42	0.02	0
PRI	1950-1955	3.48	14.78	3.89	16.14	2.72	24.99	0.17	1.80	0.00	0
	2020-2025	0.50	3.65	2.25	7.85	2.65	20.93	0.40	2.84	0.02	0
	2095-2100	0.04	1.83	1.46	2.17	1.33	4.65	0.83	1.73	0.09	0
PRK	1950-1955	0.15	1.52	0.68	0.79	0.21	0.67	0.01	0.06	0.00	0
	2020-2025	0.12	4.33	2.43	5.70	2.15	5.72	0.13	0.69	0.00	0
	2095-2100	0.02	2.32	1.67	2.81	1.51	5.85	0.48	1.46	0.00	0

Table S4. Living kin for a female Focal aged 65-69. Country-level averages for selected years (values for 2095-2100 refer to the median of the 1,000 country-level projections). Relative codes: ggd = great-grandchildren, gd = grandchildren, d = children, n = niblings, s = siblings, c = cousins, m = parents, a = aunts/uncles, gm = grandparents, ggm = great-grandparents. ***(continued)***

ISO	Year	ggd	gd	d	n	s	c	m	a	gm	ggm
PRT	1950-1955	0.27	5.08	2.47	6.31	1.77	9.86	0.10	0.94	0.00	0
	2020-2025	0.07	2.52	1.89	4.51	2.03	10.22	0.29	1.64	0.00	0
	2095-2100	0.00	1.04	1.42	2.33	1.51	4.94	0.60	1.56	0.01	0
PRY	1950-1955	3.79	20.70	4.94	25.38	3.21	38.02	0.07	1.66	0.00	0
	2020-2025	1.30	8.97	3.96	18.57	3.85	39.88	0.23	2.93	0.00	0
	2095-2100	0.14	3.13	1.94	3.43	1.60	9.41	0.51	2.11	0.02	0
PSE	1950-1955	3.65	21.91	4.58	23.30	2.63	31.70	0.05	1.29	0.00	0
	2020-2025	3.13	20.73	5.90	29.34	4.32	48.44	0.16	2.67	0.00	0
	2095-2100	0.41	5.10	2.48	5.04	2.06	18.55	0.69	4.19	0.02	0
PYF	1950-1955	3.00	15.78	3.98	17.07	2.59	25.25	0.09	1.44	0.00	0
	2020-2025	0.78	6.71	3.52	14.72	3.97	32.96	0.38	3.39	0.01	0
	2095-2100	0.03	1.97	1.63	2.74	1.62	8.24	0.85	2.53	0.06	0
QAT	1950-1955	6.44	18.31	4.27	19.53	2.92	29.94	0.11	1.80	0.00	0
	2020-2025	1.04	10.44	4.82	21.26	4.47	42.24	0.37	3.67	0.01	0
	2095-2100	0.02	2.14	1.70	2.94	1.67	12.05	0.69	2.70	0.02	0
ROU	1950-1955	0.86	6.01	2.51	6.57	1.79	10.37	0.10	0.97	0.00	0
	2020-2025	0.32	2.73	1.92	4.02	1.55	8.74	0.21	1.27	0.00	0
	2095-2100	0.02	2.07	1.68	3.03	1.63	5.23	0.59	1.50	0.02	0
RUS	1950-1955	0.36	5.17	2.24	5.53	1.31	7.61	0.05	0.57	0.00	0
	2020-2025	0.25	2.40	1.55	3.14	1.18	6.13	0.11	0.79	0.00	0
	2095-2100	0.01	1.99	1.61	2.98	1.51	4.86	0.45	1.24	0.02	0
RWA	1950-1955	2.58	17.28	4.58	21.60	2.92	32.12	0.08	1.72	0.00	0
	2020-2025	1.17	14.95	4.95	22.52	3.09	34.92	0.09	1.72	0.00	0
	2095-2100	0.14	5.14	2.61	5.42	1.83	15.91	0.24	2.17	0.00	0
SAU	1950-1955	4.85	14.35	3.77	15.42	2.30	21.88	0.09	1.37	0.00	0
	2020-2025	1.23	12.77	5.69	23.17	4.12	37.91	0.26	2.82	0.01	0
	2095-2100	0.04	2.87	1.99	3.75	1.84	15.08	0.68	3.16	0.02	0
SDN	1950-1955	3.80	15.70	4.04	17.43	2.66	26.10	0.11	1.65	0.00	0
	2020-2025	2.52	15.26	4.59	17.22	2.59	26.53	0.12	1.67	0.00	0
	2095-2100	0.33	6.71	2.90	6.43	1.84	15.79	0.18	1.90	0.00	0
SEN	1950-1955	2.90	10.89	3.16	11.03	1.83	15.02	0.05	0.90	0.00	0
	2020-2025	2.17	15.60	4.76	17.82	2.82	23.44	0.11	1.52	0.00	0
	2095-2100	0.34	7.37	3.14	7.72	2.43	21.76	0.47	3.62	0.01	0
SGP	1950-1955	4.47	22.11	4.77	24.18	2.39	30.34	0.01	0.61	0.00	0
	2020-2025	0.02	2.15	1.89	9.16	4.00	34.89	0.37	3.63	0.01	0
	2095-2100	0.00	0.76	1.17	1.66	1.27	4.09	0.66	1.44	0.01	0
SHN	1950-1955	4.35	22.04	4.79	23.88	3.55	38.41	0.14	2.08	0.00	0
	2020-2025	0.23	4.21	2.74	11.10	3.53	33.24	0.22	2.70	0.00	0
	2095-2100	0.01	1.85	1.58	2.58	1.50	5.82	0.50	1.49	0.01	0

Table S4. **Living kin for a female Focal aged 65-69. Country-level averages for selected years (values for 2095-2100 refer to the median of the 1,000 country-level projections). Relative codes: ggd = great-grandchildren, gd = grandchildren, d = children, n = niblings, s = siblings, c = cousins, m = parents, a = aunts/uncles, gm = grandparents, ggm = great-grandparents.** ***(continued)***

ISO	Year	ggd	gd	d	n	s	c	m	a	gm	ggm
SLB	1950-1955	1.28	14.26	3.83	15.94	1.87	19.49	0.03	0.69	0.00	0
	2020-2025	2.37	16.34	4.93	22.55	3.57	33.92	0.10	1.68	0.00	0
	2095-2100	0.45	6.60	2.88	6.61	2.08	18.12	0.30	2.52	0.00	0
SLE	1950-1955	1.63	5.28	2.03	4.78	1.15	6.32	0.08	0.67	0.00	0
	2020-2025	2.82	11.97	3.87	10.35	1.73	10.90	0.09	0.85	0.00	0
	2095-2100	0.33	4.39	2.24	4.19	1.48	11.44	0.17	1.59	0.00	0
SLV	1950-1955	3.57	14.19	3.39	13.39	1.90	17.74	0.06	0.96	0.00	0
	2020-2025	1.42	7.12	3.10	12.21	2.77	22.60	0.26	2.12	0.01	0
	2095-2100	0.11	2.19	1.57	2.53	1.36	6.54	0.58	1.85	0.04	0
SMR	1950-1955	0.69	6.94	2.72	7.71	1.93	11.96	0.11	1.01	0.00	0
	2020-2025	0.02	1.98	1.68	3.97	2.14	11.44	0.40	2.14	0.00	0
	2095-2100	0.00	1.03	1.28	1.81	1.29	4.10	0.71	1.58	0.01	0
SOM	1950-1955	1.54	13.79	3.95	16.37	2.19	21.56	0.04	0.96	0.00	0
	2020-2025	2.58	16.14	4.18	18.26	2.58	24.80	0.07	1.24	0.00	0
	2095-2100	0.84	9.86	3.38	7.45	1.77	18.04	0.15	1.98	0.00	0
SPM	1950-1955	1.00	10.11	3.01	10.25	1.51	12.39	0.03	0.52	0.00	0
	2020-2025	0.29	3.33	2.04	7.59	2.75	17.55	0.16	1.65	0.00	0
	2095-2100	0.02	1.93	1.63	2.55	1.47	5.59	0.66	1.61	0.02	0
SRB	1950-1955	0.62	4.83	2.23	5.21	1.62	8.35	0.11	0.94	0.00	0
	2020-2025	0.16	2.23	1.55	2.76	1.26	6.19	0.19	1.15	0.00	0
	2095-2100	0.01	1.53	1.54	2.62	1.54	4.95	0.51	1.39	0.01	0
SSD	1950-1955	1.35	7.32	2.54	7.18	1.21	8.67	0.02	0.52	0.00	0
	2020-2025	1.71	9.41	3.34	8.02	1.08	8.48	0.04	0.54	0.00	0
	2095-2100	0.41	4.86	2.40	4.32	1.43	11.58	0.16	1.54	0.00	0
STP	1950-1955	2.83	11.62	3.13	11.02	1.84	14.47	0.06	0.75	0.00	0
	2020-2025	2.82	15.62	4.52	18.74	2.82	22.73	0.08	1.00	0.00	0
	2095-2100	0.41	5.49	2.62	5.59	1.78	15.41	0.24	2.15	0.00	0
SUR	1950-1955	5.81	22.76	4.60	23.50	2.38	30.57	0.01	0.76	0.00	0
	2020-2025	1.42	7.68	3.15	14.89	3.36	33.93	0.16	2.27	0.00	0
	2095-2100	0.08	2.81	1.92	3.50	1.61	8.48	0.46	1.89	0.02	0
SVK	1950-1955	1.19	8.04	2.90	8.75	2.15	14.03	0.13	1.25	0.00	0
	2020-2025	0.28	2.96	2.03	5.06	1.85	11.31	0.19	1.42	0.00	0
	2095-2100	0.03	1.99	1.68	2.60	1.45	5.05	0.59	1.52	0.02	0
SVN	1950-1955	0.25	4.45	2.21	5.11	1.47	7.68	0.07	0.70	0.00	0
	2020-2025	0.12	2.55	1.71	3.18	1.50	7.13	0.25	1.37	0.00	0
	2095-2100	0.01	1.85	1.66	2.86	1.64	5.57	0.71	1.77	0.03	0
SWE	1950-1955	0.37	3.68	1.99	4.06	1.55	6.60	0.17	0.87	0.00	0
	2020-2025	0.08	2.93	1.84	3.68	1.69	6.90	0.37	1.37	0.00	0
	2095-2100	0.01	2.01	1.67	2.88	1.66	6.48	0.74	2.14	0.01	0

Table S4. Living kin for a female Focal aged 65-69. Country-level averages for selected years (values for 2095-2100 refer to the median of the 1,000 country-level projections). Relative codes: ggd = great-grandchildren, gd = grandchildren, d = children, n = niblings, s = siblings, c = cousins, m = parents, a = aunts/uncles, gm = grandparents, ggm = great-grandparents. ***(continued)***

ISO	Year	ggd	gd	d	n	s	c	m	a	gm	ggm
SWZ	1950-1955	3.43	13.50	3.61	14.29	2.01	19.11	0.06	1.04	0.00	0
	2020-2025	2.06	10.35	3.58	14.16	1.76	18.10	0.10	1.02	0.00	0
	2095-2100	0.23	3.56	1.95	3.31	1.20	7.40	0.17	1.06	0.00	0
SXM	1950-1955	1.54	14.43	4.10	17.42	2.97	27.43	0.10	1.56	0.00	0
	2020-2025	0.29	3.33	2.18	7.63	2.84	22.38	0.17	2.15	0.00	0
	2095-2100	0.02	1.80	1.56	2.54	1.50	5.39	0.55	1.38	0.01	0
SYC	1950-1955	2.06	12.48	3.44	13.16	1.57	15.35	0.01	0.46	0.00	0
	2020-2025	0.94	5.58	2.85	10.77	2.81	22.26	0.11	1.41	0.00	0
	2095-2100	0.12	3.12	1.93	3.55	1.70	7.54	0.59	1.93	0.02	0
SYR	1950-1955	2.35	17.47	4.46	20.98	2.72	29.85	0.06	1.34	0.00	0
	2020-2025	1.50	14.46	5.27	24.41	4.03	42.31	0.14	2.25	0.00	0
	2095-2100	0.11	3.42	2.05	3.66	1.64	11.92	0.43	2.40	0.01	0
TCA	1950-1955	2.28	13.15	3.59	14.00	2.25	19.92	0.07	1.17	0.00	0
	2020-2025	0.42	5.99	3.87	14.04	3.42	27.69	0.22	2.30	0.00	0
	2095-2100	0.02	1.73	1.57	2.58	1.48	8.04	0.42	1.52	0.01	0
TCD	1950-1955	2.08	8.58	2.65	8.06	1.45	10.40	0.05	0.68	0.00	0
	2020-2025	4.69	16.74	3.77	13.05	1.79	13.67	0.08	0.87	0.00	0
	2095-2100	0.70	10.53	3.51	8.18	1.74	18.69	0.12	1.80	0.00	0
TGO	1950-1955	2.62	12.27	3.40	12.72	1.99	17.40	0.04	0.94	0.00	0
	2020-2025	2.21	13.81	4.31	17.52	2.86	24.42	0.08	1.37	0.00	0
	2095-2100	0.64	6.90	2.78	6.03	1.80	14.98	0.17	1.78	0.00	0
THA	1950-1955	1.53	13.94	3.68	15.01	1.79	18.31	0.04	0.76	0.00	0
	2020-2025	0.35	3.30	2.30	9.17	3.23	24.75	0.31	2.57	0.01	0
	2095-2100	0.02	1.39	1.39	2.13	1.35	4.48	0.70	1.48	0.06	0
TJK	1950-1955	1.01	10.79	3.60	13.46	2.45	20.83	0.08	1.39	0.00	0
	2020-2025	2.35	12.31	4.24	17.66	3.25	27.25	0.11	1.66	0.00	0
	2095-2100	1.30	4.91	2.34	4.55	1.74	11.61	0.46	2.31	0.01	0
TKL	1950-1955	2.77	15.81	4.08	17.75	2.29	23.68	0.05	0.99	0.00	0
	2020-2025	1.01	9.73	3.85	16.97	3.46	31.19	0.19	2.25	0.00	0
	2095-2100	0.05	3.26	2.11	3.95	1.79	12.28	0.52	2.70	0.02	0
TKM	1950-1955	0.93	10.55	3.47	12.72	2.09	18.02	0.05	1.00	0.00	0
	2020-2025	1.13	9.46	3.79	15.18	3.20	25.34	0.10	1.53	0.00	0
	2095-2100	0.67	5.05	2.11	4.32	1.57	9.34	0.27	1.54	0.00	0
TLS	1950-1955	0.66	7.28	2.53	7.15	1.02	7.67	0.01	0.32	0.00	0
	2020-2025	0.71	9.06	3.43	8.88	1.43	10.20	0.05	0.58	0.00	0
	2095-2100	0.19	3.93	2.09	3.77	1.61	12.25	0.40	2.61	0.01	0
TON	1950-1955	1.86	21.26	4.92	25.71	3.02	36.76	0.05	1.36	0.00	0
	2020-2025	0.64	12.56	4.59	23.22	3.82	42.66	0.11	2.07	0.00	0
	2095-2100	0.16	5.53	2.57	5.35	1.86	14.76	0.33	2.31	0.00	0

Table S4. **Living kin for a female Focal aged 65-69. Country-level averages for selected years (values for 2095-2100 refer to the median of the 1,000 country-level projections). Relative codes: ggd = great-grandchildren, gd = grandchildren, d = children, n = niblings, s = siblings, c = cousins, m = parents, a = aunts/uncles, gm = grandparents, ggm = great-grandparents.** ***(continued)***

ISO	Year	ggd	gd	d	n	s	c	m	a	gm	ggm
TTO	1950-1955	5.23	16.18	3.87	16.43	2.28	22.54	0.05	0.96	0.00	0
	2020-2025	0.44	4.02	2.62	9.87	2.95	23.92	0.24	2.22	0.00	0
	2095-2100	0.06	2.11	1.60	2.40	1.33	4.94	0.57	1.44	0.03	0
TUN	1950-1955	1.53	11.65	3.32	12.20	1.72	15.24	0.04	0.69	0.00	0
	2020-2025	0.23	6.78	3.99	14.58	3.60	28.00	0.20	2.06	0.00	0
	2095-2100	0.03	2.57	1.87	3.23	1.66	8.84	0.56	2.05	0.01	0
TUV	1950-1955	0.83	8.73	2.70	8.38	1.33	9.95	0.03	0.47	0.00	0
	2020-2025	0.78	8.20	3.01	10.10	1.88	13.40	0.06	0.78	0.00	0
	2095-2100	0.32	5.15	2.46	5.09	1.57	10.77	0.16	1.18	0.00	0
TWN	1950-1955	2.45	22.53	5.27	28.58	3.15	40.59	0.02	1.07	0.00	0
	2020-2025	0.08	3.20	2.34	10.38	3.53	34.88	0.29	3.38	0.00	0
	2095-2100	0.00	0.66	1.32	2.20	1.46	5.21	0.53	1.43	0.01	0
TZA	1950-1955	2.75	11.77	3.35	12.35	1.89	16.66	0.06	1.01	0.00	0
	2020-2025	3.20	15.49	4.31	16.61	2.46	22.09	0.11	1.39	0.00	0
	2095-2100	0.63	6.89	2.99	6.80	2.08	18.24	0.36	2.87	0.01	0
UGA	1950-1955	3.36	12.37	3.23	11.94	1.69	15.02	0.07	0.87	0.00	0
	2020-2025	4.24	17.48	4.20	17.35	2.10	19.56	0.06	0.92	0.00	0
	2095-2100	0.47	5.45	2.57	5.35	1.65	15.98	0.16	2.01	0.00	0
UKR	1950-1955	0.29	3.83	1.97	4.14	1.29	6.12	0.07	0.60	0.00	0
	2020-2025	0.29	2.21	1.57	2.85	1.13	5.11	0.12	0.72	0.00	0
	2095-2100	0.02	1.41	1.46	2.43	1.42	4.27	0.52	1.25	0.03	0
URY	1950-1955	0.65	4.75	2.24	5.23	1.58	8.11	0.13	0.89	0.00	0
	2020-2025	0.44	3.96	2.31	5.20	1.86	9.19	0.26	1.33	0.01	0
	2095-2100	0.03	1.65	1.50	2.45	1.47	5.78	0.63	1.76	0.04	0
USA	1950-1955	1.70	7.36	2.69	7.57	1.90	11.63	0.16	1.10	0.00	0
	2020-2025	0.25	3.14	1.73	4.98	2.20	11.61	0.41	1.95	0.01	0
	2095-2100	0.01	2.00	1.62	2.90	1.62	6.23	0.74	2.01	0.04	0
UZB	1950-1955	1.73	13.32	3.80	15.26	2.64	23.86	0.10	1.52	0.00	0
	2020-2025	1.71	9.12	3.73	15.84	3.53	29.61	0.12	1.84	0.00	0
	2095-2100	0.26	4.07	2.20	4.19	1.69	9.33	0.35	1.70	0.00	0
VCT	1950-1955	8.22	23.94	4.69	24.53	2.93	34.86	0.09	1.60	0.00	0
	2020-2025	1.05	6.00	3.13	15.23	3.92	38.66	0.32	3.29	0.01	0
	2095-2100	0.07	2.15	1.66	2.80	1.47	6.59	0.56	1.74	0.03	0
VEN	1950-1955	5.21	20.40	4.32	20.77	2.58	28.92	0.08	1.36	0.00	0
	2020-2025	1.48	7.19	3.33	14.54	3.65	33.80	0.23	2.80	0.00	0
	2095-2100	0.20	2.81	1.78	2.98	1.47	6.95	0.59	1.88	0.02	0
VGB	1950-1955	3.47	14.91	3.96	16.43	2.60	24.51	0.09	1.49	0.00	0
	2020-2025	0.20	2.76	2.33	7.74	3.04	23.57	0.24	2.62	0.00	0
	2095-2100	0.01	1.04	1.17	1.74	1.25	3.48	0.59	1.08	0.02	0

Table S4. Living kin for a female Focal aged 65-69. Country-level averages for selected years (values for 2095-2100 refer to the median of the 1,000 country-level projections). Relative codes: ggd = great-grandchildren, gd = grandchildren, d = children, n = niblings, s = siblings, c = cousins, m = parents, a = aunts/uncles, gm = grandparents, ggm = great-grandparents. ***(continued)***

ISO	Year	ggd	gd	d	n	s	c	m	a	gm	ggm
	1950-1955	3.37	12.35	3.38	12.56	2.16	18.03	0.09	1.17	0.00	0
VIR	2020-2025	1.30	6.42	2.91	10.66	2.78	20.85	0.16	1.70	0.00	0
	2095-2100	0.08	3.03	1.90	3.43	1.66	7.96	0.59	1.98	0.02	0
	1950-1955	0.47	10.88	3.30	12.00	1.79	15.02	0.04	0.71	0.00	0
VNM	2020-2025	0.53	5.79	3.30	12.45	3.34	24.99	0.13	1.53	0.00	0
	2095-2100	0.02	2.40	1.78	3.40	1.74	7.09	0.58	1.72	0.02	0
	1950-1955	3.29	19.87	4.23	20.19	2.27	26.14	0.04	1.00	0.00	0
VUT	2020-2025	2.43	14.59	4.68	21.93	3.64	37.74	0.12	1.99	0.00	0
	2095-2100	0.58	6.67	2.87	6.68	2.15	17.31	0.39	2.67	0.01	0
	1950-1955	4.11	24.52	4.99	26.92	2.91	37.13	0.05	1.36	0.00	0
WSM	2020-2025	1.91	17.32	5.29	27.85	4.42	51.54	0.14	2.53	0.00	0
	2095-2100	0.59	6.88	2.90	6.51	2.16	18.54	0.49	3.22	0.01	0
	1950-1955	0.90	8.71	3.19	10.63	1.72	13.98	0.04	0.77	0.00	0
YEM	2020-2025	2.69	18.05	5.62	20.44	2.88	24.35	0.10	1.37	0.00	0
	2095-2100	0.18	4.74	2.46	4.85	1.69	16.19	0.22	2.19	0.00	0
	1950-1955	1.96	12.70	3.64	14.18	2.09	19.12	0.08	1.06	0.00	0
ZAF	2020-2025	0.90	6.22	3.08	11.65	2.27	19.66	0.14	1.38	0.00	0
	2095-2100	0.20	3.01	1.76	2.87	1.13	5.47	0.27	0.99	0.02	0
	1950-1955	3.25	13.57	3.75	15.05	2.38	21.95	0.10	1.46	0.00	0
ZMB	2020-2025	3.59	15.78	4.24	18.60	2.45	24.24	0.09	1.29	0.00	0
	2095-2100	0.60	6.20	2.77	6.02	1.98	16.70	0.34	2.72	0.01	0
	1950-1955	4.78	18.11	4.41	20.69	2.71	29.61	0.09	1.60	0.00	0
ZWE	2020-2025	2.39	11.73	3.82	16.89	2.21	25.28	0.09	1.28	0.00	0
	2095-2100	0.28	4.60	2.31	4.37	1.41	9.58	0.18	1.35	0.00	0

References

1. H Caswell, The formal demography of kinship: A matrix formulation. *Demogr. Res.* **41**, 679–712 (2019).
2. H Caswell, The formal demography of kinship II: Multistate models, parity, and sibship. *Demogr. Res.* **42**, 1097–1146 (2020).
3. H Caswell, X Song, The formal demography of kinship III: Kinship dynamics with time-varying demographic rates. *Demogr. Res.* **45**, 517–546 (2021).
4. H Caswell, The formal demography of kinship IV: Two-sex models and their approximations. *Demogr. Res.* **47**, 359–396 (2022).
5. H Caswell, R Margolis, AM Verdery, The formal demography of kinship V: Kin loss, bereavement, and causes of death, (SocArXiv), preprint (2023).
6. I Williams, D Alburez-Gutierrez, H Caswell, X Song, DemoKin: 1.0.3 (https://CRAN.R-project.org/package=DemoKin) (2023).
7. UN DESA, World Population Prospects 2022: Summary of Results, (United Nations Department of Economic and Social Affairs, Population Division), Technical Report UN DESA/POP/2022/TR/NO. 3 (2022).
8. N Keyfitz, *Applied mathematical demography.* (Springer, New York), (1985) OCLC: 610135904.
9. SH Preston, P Heuveline, M Guillot, *Demography: measuring and modeling population processes.* (Blackwell Publishers, Malden, MA), (2001).
10. LA Goodman, N Keyfitz, TW Pullum, Family formation and the frequency of various kinship relationships. *Theor. Popul. Biol.* **5**, 1–27 (1974).
11. M Kolk, L Andersson, E Pettersson, S Drefahl, The Swedish Kinship Universe: A Demographic Account of the Number of Children, Parents, Siblings, Grandchildren, Grandparents, Aunts/Uncles, Nieces/Nephews, and Cousins Using National Population Registers. *Demography* p. 10955240 (2023).
12. D Alburez-Gutierrez, C Mason, E Zagheni, The "Sandwich Generation" Revisited: Global Demographic Drivers of Care Time Demands. *Popul. Dev. Rev.* **47**, 997–1023 (2021).
13. M Snyder, D Alburez-Gutierrez, I Williams, E Zagheni, Estimates from 31 countries show the significant impact of COVID-19 excess mortality on the incidence of family bereavement. *Proc. Natl. Acad. Sci.* **119**, e2202686119 (2022).
14. D Alburez-Gutierrez, I Williams, H Caswell, Replication Data for "Projections of human kinship for all countries" (2023) Harvard Dataverse V.1. https://doi.org/10.7910/DVN/FKCRHW.
